# Novel Tri-Segmented Rhabdoviruses: A Data Mining Expedition Unveils the Cryptic Diversity of Cytorhabdoviruses

**DOI:** 10.3390/v15122402

**Published:** 2023-12-10

**Authors:** Nicolas Bejerman, Ralf Dietzgen, Humberto Debat

**Affiliations:** 1 Instituto de Patología Vegetal—Centro de Investigaciones Agropecuarias—Instituto Nacional de Tecnología Agropecuaria (IPAVE—CIAP—INTA), Camino 60 Cuadras Km 5,5, Córdoba X5020ICA, Argentina; 2 Unidad de Fitopatología y Modelización Agrícola, Consejo Nacional de Investigaciones Científicas y Técnicas, Camino 60 Cuadras Km 5,5, Córdoba X5020ICA, Argentina; 3 Queensland Alliance for Agriculture and Food Innovation, The University of Queensland, St. Lucia, QLD 4072, Australia

**Keywords:** tri-segmented viruses, cytorhabdoviruses, virus taxonomy, metatranscriptomics, virus discovery, genetic diversity

## Abstract

Cytorhabdoviruses (genus *Cytorhabdovirus*, family *Rhabdoviridae*) are plant-infecting viruses with enveloped, bacilliform virions. Established members of the genus *Cytorhabdovirus* have unsegmented single-stranded negative-sense RNA genomes (ca. 10–16 kb) which encode four to ten proteins. Here, by exploring large publicly available metatranscriptomics datasets, we report the identification and genomic characterization of 93 novel viruses with genetic and evolutionary cues of cytorhabdoviruses. Strikingly, five unprecedented viruses with tri-segmented genomes were also identified. This finding represents the first tri-segmented viruses in the family *Rhabdoviridae*, and they should be classified in a novel genus within this family for which we suggest the name “*Trirhavirus*”. Interestingly, the nucleocapsid and polymerase were the only typical rhabdoviral proteins encoded by those tri-segmented viruses, whereas in three of them, a protein similar to the emaravirus (family *Fimoviridae*) silencing suppressor was found, while the other predicted proteins had no matches in any sequence databases. Genetic distance and evolutionary insights suggest that all these novel viruses may represent members of novel species. Phylogenetic analyses, of both novel and previously classified plant rhabdoviruses, provide compelling support for the division of the genus *Cytorhabdovirus* into three distinct genera. This proposed reclassification not only enhances our understanding of the evolutionary dynamics within this group of plant rhabdoviruses but also illuminates the remarkable genomic diversity they encompass. This study not only represents a significant expansion of the genomics of cytorhabdoviruses that will enable future research on the evolutionary peculiarity of this genus but also shows the plasticity in the rhabdovirus genome organization with the discovery of tri-segmented members with a unique evolutionary trajectory.

## 1. Introduction

In the current metagenomics era, the rapid discovery of novel viruses has unveiled a rich and diverse evolutionary landscape of replicating entities, that present intricate challenges in their systematic classification [1]. To address this phenomenon, diverse strategies have emerged, culminating in a comprehensive proposal for establishing a megataxonomy of the virus world [2]. However, despite extensive efforts to characterize the viral component of the biosphere, it is evident that only a minuscule fraction, likely encompassing less than one percent of the entire virosphere, has been comprehensively characterized to date [3,4]. Consequently, our understanding of the vast global virome remains limited, with its remarkable diversity and its interactions with various host organisms [5,6,7,8]. To fill this knowledge gap, researchers have used the mining of publicly available transcriptome datasets obtained through High-Throughput Sequencing (HTS) as an efficient and inexpensive strategy [6,9,10,11]. This data-driven approach to virus discovery has become increasingly valuable, given the wealth of freely available datasets within the Sequence Read Archive (SRA) maintained by the National Center for Biotechnology Information (NCBI), which is continually expanding at an extraordinary rate. These data represents a substantial, albeit still somewhat limited and potentially biased, portion of the organisms inhabiting our world, thus making the NCBI-SRA database a cost-effective and efficient resource for the identification of novel viruses [12]. *Serratus* [6] has become an invaluable and exciting tool that facilitates comprehensive data mining, thus accelerating virus sequence discovery at a pace never witnessed before. In terms of virus taxonomy, a consensus statement has emphasized the importance of incorporating viruses known solely based on metagenomic data into the official classification scheme of the International Committee on Taxonomy of Viruses (ICTV) [13]. This recognition underscores the significance of metagenomic approaches in expanding our understanding of the global virome and adapting taxonomic frameworks to accommodate the ever-expanding diversity of viruses [14].

The family *Rhabdoviridae* is composed of members with negative-sense single-stranded RNA genomes that infect a broad range of hosts including plants, amphibians, fish, mammals, reptiles, insects, and other arthropods, and they include many pathogens of significance to public health, agriculture, and fisheries [15,16]. Almost all rhabdovirus genomes are unsegmented, but interestingly, plant-associated rhabdoviruses with bi-segmented genomes and a shared evolutionary history of rhabdoviruses have been included in the family in both genera *Dichorhavirus* and *Varicosavirus* [15,16]. *Cytorhabdovirus* is one of the genera that include plant-infecting viruses (family *Rhabdoviridae*, subfamily *Betarhabdovirinae*) [16]. Most cytorhabdoviruses exhibit a genome organization characterized by the presence of six conserved canonical genes encoded in the order 3′– nucleocapsid protein (N) – phosphoprotein (P) – movement protein (P3) – matrix protein (M) – glycoprotein (G) – large polymerase (L) –5′, and up to four additional accessory genes with unknown functions, leading to diverse genome organizations [17]. With some exceptions, the presence and synteny of the canonical genes are strictly conserved, nevertheless, some cytorhabdoviruses lack the G gene [9]. The viral genes are separated by conserved gene junction sequences, and the whole coding region is flanked by 3′ leader and 5′ trailer sequences that possess partially complementary ends, which could form a panhandle structure during viral replication [15]. 

In this study, through mining of publicly available sequence data, we identified 93 novel cytorhabdoviruses including five viruses with an unprecedented tri-segmented genome, which represent the first tri-segmented genomes among rhabdoviruses. Our findings will significantly advance the taxonomical classification of cytorhabdoviruses, allowing us to split this genus into three genera and shed new light on the evolutionary landscape of this group of plant rhabdoviruses. 

## 2. Material and Methods

### 2.1. Identification of Cytorhabdovirus-Like Sequences from Public Plant RNA-Seq Datasets

We analyzed the Serratus database using the Serratus Explorer tool v1 [6] and queried the predicted RNA-dependent RNA polymerase protein (RdRP) of cytorhabdoviruses available at the NCBI-refseq database. The SRA libraries that matched the query sequences (alignment identity > 45%; score > 10) were further explored in detail.

### 2.2. Sequence Assembly and Virus Identification

Virus discovery was implemented as described elsewhere [10,11]. In brief, the raw nucleotide sequence reads from each SRA experiment that matched the query sequences in the Serratus platform were downloaded from their associated NCBI BioProjects. The datasets were pre-processed by trimming and filtering with the Trimmomatic v0.40 tool as implemented in http://www.usadellab.org/cms/?page=trimmomatic (accessed on 6 October 2023) with standard parameters. The resulting reads were assembled de novo with rnaSPAdes using standard parameters on the Galaxy server (https://usegalaxy.org/, accessed on 6 October 2023). The transcripts obtained from de novo transcriptome assembly were subjected to bulk local BLASTX searches (*E*-value < 1 × 10^−5^) against cytorhabdovirus refseq protein sequences available at https://www.ncbi.nlm.nih.gov/protein?term=txid11305[Organism], accessed on 6 October 2023. The resulting viral sequence hits of each dataset were explored in detail. Tentative virus-like contigs were curated (extended and/or confirmed) by iterative mapping of each SRA library’s filtered reads. This strategy was used to extract a subset of reads related to the query contig, used the retrieved reads from each mapping to extend the contig and then repeat the process iteratively using as query the extended sequence [10]. The extended and polished transcripts were reassembled using Geneious v8.1.9 (Biomatters Ltd., Auckland, New Zealand) alignment tool with high sensitivity parameters. 

### 2.3. Bioinformatics Tools and Analyses

#### 2.3.1. Sequence Analyses

ORFs were predicted with ORFfinder (minimal ORF length 120 nt, genetic code 1, https://www.ncbi.nlm.nih.gov/orffinder/, accessed on 6 October 2023), functional domains and architecture of translated gene products were determined using InterPro (https://www.ebi.ac.uk/interpro/search/sequence-search, accessed on 6 October 2023) and the NCBI Conserved domain database—CDD v3.20 (https://www.ncbi.nlm.nih.gov/Structure/cdd/wrpsb.cgi, accessed on 6 October 2023) with *E*-value = 0.1. Further, HHPred and HHBlits as implemented in https://toolkit.tuebingen.mpg.de/#/tools/, accessed on 6 October 2023, were used to complement annotation of divergent predicted proteins by hidden Markov models. Transmembrane domains were predicted using the TMHMM version 2.0 tool (http://www.cbs.dtu.dk/services/TMHMM/, accessed on 6 October 2023) and signal peptides were predicted using the SignalP version 6.0 tool (https://services.healthtech.dtu.dk/services/SignalP-6.0/, accessed on 6 October 2023). The presence of gene junction sequences flanking ORFs was also included as a criterion to determine the potential coding sequences. The predicted proteins were then subjected to NCBI-BLASTP searches against the non-redundant protein sequences (nr) database.

#### 2.3.2. Pairwise Sequence Identity

Percentage amino acid (aa) sequence identities of the predicted L protein of all viruses identified in this study, as well as those available in the NCBI database were calculated using SDTv1.2 [18] based on MAFFT 7.505 (https://mafft.cbrc.jp/alignment/software, accessed on 6 October 2023), alignments with standard parameters. Virus names and abbreviations of cytorhabdoviruses already reported are shown in Supplementary Table S1.

#### 2.3.3. Phylogenetic Analysis

Phylogenetic analysis based on the predicted L and N proteins of all plant cytorhabdoviruses, listed in Table S1, was conducted using MAFFT 7.505 with multiple aa sequence alignments using FFT-NS-i. The aligned aa sequences were used as the input in MEGA11 software [19] to generate phylogenetic trees by the maximum-likelihood method (best-fit model = WAG + G + F). Local support values were computed using bootstraps with 1000 replicates. L and N proteins of selected varicosaviruses and alphanucleorhabdoviruses were used as outgroups. 

## 3. Results

### 3.1. Summary of Discovered Viral Sequences

**Figure 1:**
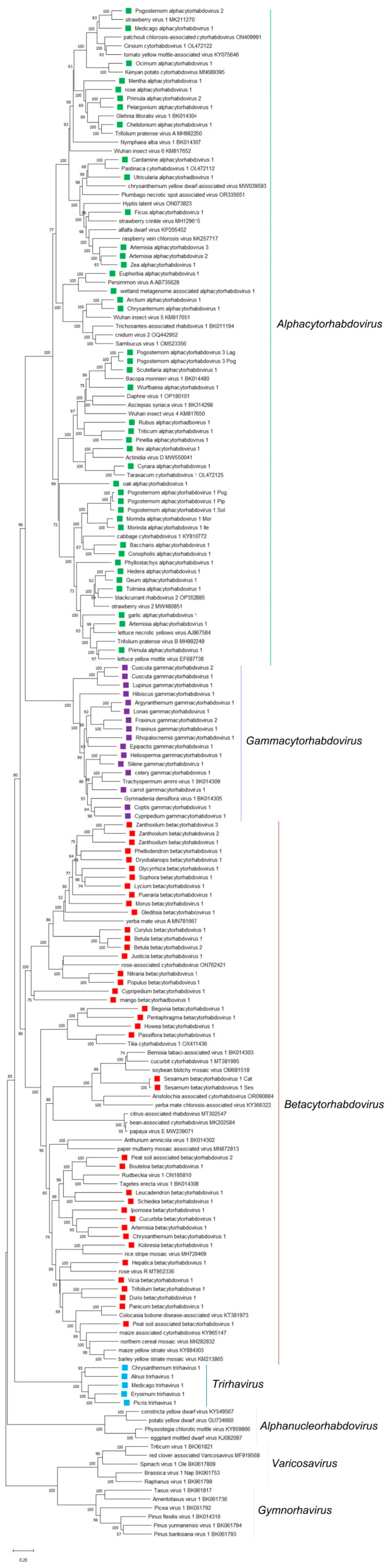
Maximum-likelihood phylogenetic tree based on amino acid sequence alignments of the complete L gene of all tri-segmented rhabdoviruses and cytorhabdoviruses reported so far and in this study constructed with the WAG + G + F model. The scale bar indicates the number of substitutions per site. Bootstrap values following 1000 replicates are given at the nodes, but only the values above 50% are shown. The viruses identified in this study are noted with green, red, violet, and blue rectangles according to proposed genus membership. *Alphanucleorhabdoviruses*, *gymnorhaviruses* and *varicosaviruses* were used as outgroups.

In this study, through identification, assembly, and curation of raw NCBI-SRA reads of publicly available transcriptomic data we obtained the coding complete viral genomic sequences of 93 novel viruses with genetic and evolutionary links to cytorhabdoviruses. The phylogenetic relationships of the now significantly expanded number of known cytorhabdoviruses provide support for splitting the genus *Cytorhabdovirus* to establish three genera (Figure 1) that represent distinct evolutionary lineages, which we propose to name *Alphacytorhabdovirus* (Table 1), *Betacytorhabdovirus* (Table 2) and *Gammacytorhabdovirus* (Table 3). Strikingly, five unprecedented viruses with a tri-segmented genome were also identified and their full-length viral genomic sequences were assembled (Table 4), including the corrected full-length coding genome segments of the previously reported Picris cytorhabdovirus 1 (PiCRV1) [20], which had one RNA segment missing, as well as its RNA2 partially annotated.

### 3.2. Genus Alphacytorhabdovirus

The full-length coding regions of 38 novel putative alphacytorhabdoviruses were assembled in this study, including three variants of the same virus associated with different plant hosts, and two host variants of two other viruses (Table 1). The newly identified viruses were associated with 39 plant host species and a wetland metagenome study (Table 1). Most of the apparent host plants are herbaceous dicots (27/39), while 6 hosts are monocots and another 6 are woody dicots (Table 1).

**Figure 2:**
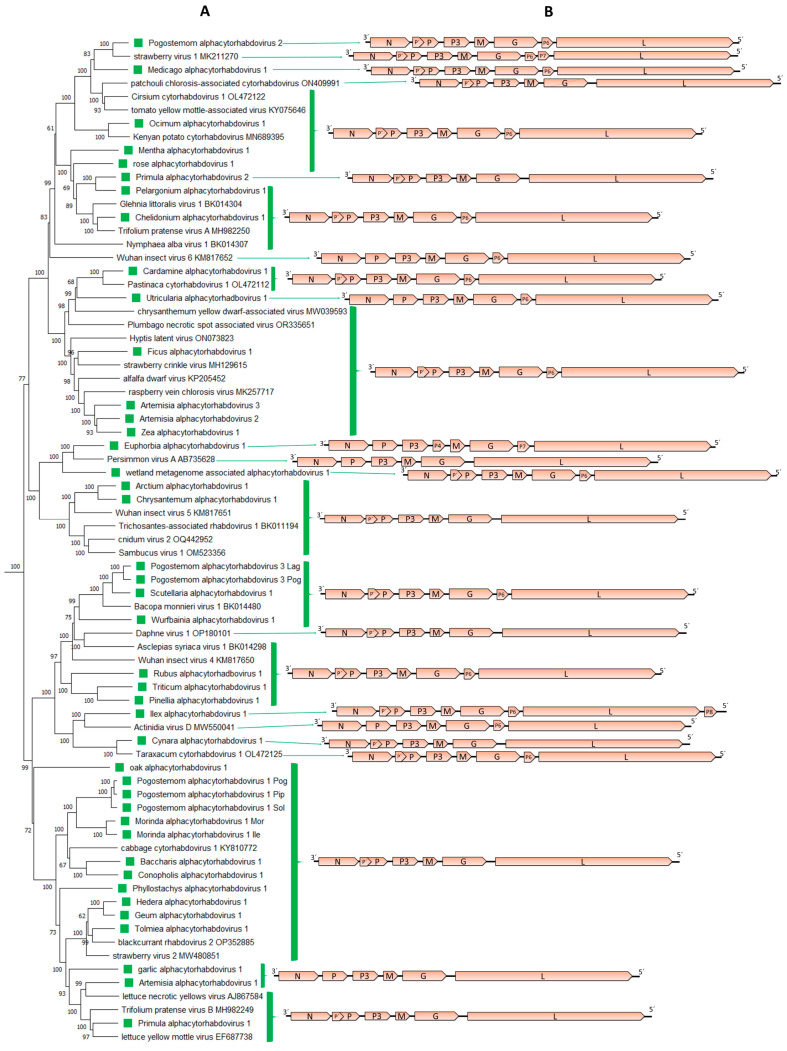
(**A**): An inset of the maximum-likelihood phylogenetic tree shown in Figure 1 was cropped to show those viruses included in the proposed genus *Alphacytorhabdovirus*. The viruses identified in this study are noted with green squares. (**B**): genomic organization of the viral sequences used in the phylogeny.

The genomic organization of the 38 novel alphacytorhabdoviruses was quite similar, with few exceptions, with six distinct genomic organizations observed (Table 1, Figure 2B). Two virus genomes have no additional accessory genes and have the genome organization 3′-N-P–P3-M-G-L-5′ (Table 1, Figure 2B), while 14 viruses had an overlapping ORF within the P-encoding ORF, named P′, one virus had an accessory ORF between the G and L genes displaying a 3′-N-P–P3-M-G-P6-L-5′ genomic organization and 20 viruses had both those accessory ORFs (Table 1, Figure 2B). Another virus also had two accessory ORFs, one located between the P3 and the M genes, and the other between the G and L genes, displaying a 3′-N-P–P3-P4-M-G-P7-L-5′ genomic organization (Table 1, Figure 2B). Another newly identified virus also had two accessory ORFs, one between the G and L genes, and the other following the L gene, showing a 3′-N-P–P3-M-G-P6-L-P8-5′ genomic organization (Table 1, Figure 2B). P4 and P8 proteins yielded no hits when BlastP searches were carried out, and no conserved domains were identified in these proteins. On the other hand, transmembrane domains were identified in each P′ protein, as well as in each protein encoded by the accessory ORF located between the G and L genes.

The consensus gene junction sequences of the novel alphacytorhabdoviruses identified in our study were highly similar to those of previously reported phylogenetically related cytorhabdoviruses (Table 5). 

Pairwise aa sequence identity values between each of the L proteins of the 38 novel viruses and those from known alphacytorhabdoviruses varied significantly, ranging from 36.16% to 85.65% (Table S2), while sequence identity for variants of the same virus ranged from 89.01% to 96.28% (Table S2). On the other hand, the highest L protein aa sequence identity with those cytorhabdoviruses proposed to be classified as betacytorhaboviruses and gammacytorhabdoviruses was 33.84% (Table S2).

A phylogenetic analysis based on the L protein aa sequence showed that the 38 novel viruses grouped with 33 known cytorhabdoviruses in a distinct major cluster (Figure 1). Within this cluster of 71 viruses, several clades could be distinguished (Figure 2A). One major clade and other minor ones were composed of viruses that do not have accessory ORFs between the G and L genes (Figure 2), while another clade grouped together viruses with an accessory ORF between the G and L genes (Figure 2). Other clusters grouped together viruses with distinct genomic organizations (Figure 2). A similar topology was observed in the phylogenetic tree based on the N protein aa sequences (Figure S1).

### 3.3. Genus Betacytorhabdovirus

The full-length coding regions of 39 novel putative betacytorhabdoviruses were assembled in this study (Table 2), including two distinct variants of the same virus. Based on the database information, the identified viruses were associated with 36 plant host species and two peat soil metagenomes (Table 2). Interestingly, 18/36 hosts are woody dicots, while 13/36 hosts are herbaceous dicots, and the other five hosts are monocots (Table 2).

**Figure 3:**
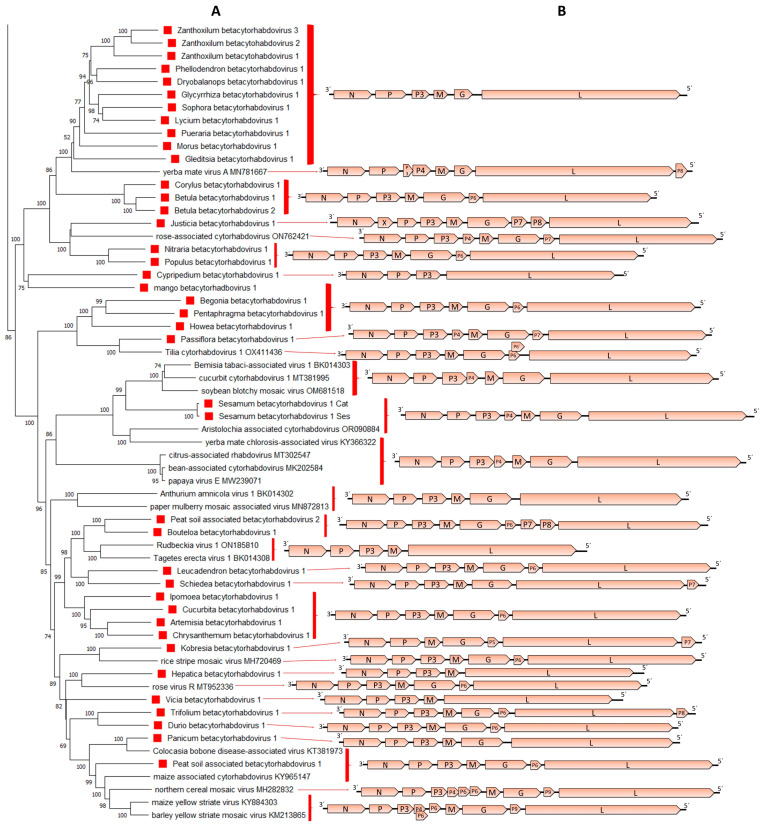
(**A**): An inset of the maximum-likelihood phylogenetic tree shown in Figure 1 was cropped to show those viruses included in the proposed genus *Betacytorhabdovirus*. The viruses identified in this study are noted with red squares. (**B**): genomic organization of the viral sequences used in the phylogeny.

The genomic organization of the 39 novel betacytorhabdoviruses was quite diverse, with 12 distinct genomic organizations observed (Table 2, Figure 3B). Several (12/39) viruses lack additional accessory genes and have the conserved basic genome organization 3′-N-P–P3-M-G-L-5′, but 11 of those genomes have a significantly shorter G gene (Table 2, Figure 3B). Other viruses (16/39) had an accessory ORF between the G and L genes displaying a 3′-N-P–P3-M-G-P6-L-5′ genomic organization. One virus had an accessory ORF, located after the L gene, thus displaying a 3′-N-P–P3-M-G-L-P7-5′ genomic organization, and one virus had an accessory ORF between the P3 and M genes showing a 3′-N-P–P3-P4-M-G-L-5′ genome organization (Table 2, Figure 3B). One virus had two accessory ORFs, one between the G and L genes, and the other after the L gene showing a 3′-N-P–P3-M-G-P6-L-P8-5′ genome organization (Table 2, Figure 3B). Yet another virus had two accessory ORFs in the same position; however, this virus lacked a discernable P3 gene, thus displaying a 3′-N-P–M-G-P5-L-P7-5′ genome organization (Table 2, Figure 3B). Four viruses had three accessory ORFs each in their genome, located either between the G and L genes displaying a 3′-N-P–P3-M-G-P6-P7-P8-L-5′ genome organization, or two accessory ORFs between the G and L genes and another between the N and P genes showing a 3′-N-X-P–P3-M-G-P7-P8-L-5′ genome organization or two accessory ORFs located between the P3 and the M genes, and another one after the L gene, displaying a 3′-N-P–P3-P4-M-G-L-P7-5′ genomic organization (Table 2, Figure 3B). On the other hand, one virus appeared to only have four genes in the order 3′-N-P-P3-L-5′, while the genome of two other viruses had five genes in the order 3′-N-P–P3-M-L-5′ but lacking the G gene (Table 2, Figure 3B). The P4 protein encoded by the virus named Passiflora betacytorhabdovirus 1 showed no hits when BlastP searches were carried out, and no known conserved domains were identified, whereas the P4 protein encoded by Sesamum virus 1 had no hits against the database, but a transmembrane domain and a Signal peptide were predicted. No hits against the database, nor conserved domains were found in those proteins encoded by the accessory ORF located after the L gene, or by the ORFs located after the accessory ORF which encodes the P6 protein in those viruses that have more than one accessory ORF between the G and L genes. Transmembrane domains were identified in the P6 protein which is encoded by an accessory ORF located between the G and L genes in those viruses, named P5 in Kobresia betacytorhabdovirus 1 and P7 in Justicia betacytorhabdovirus 1 and Passiflora betacytorhabdovirus 1.

The consensus gene junction sequences among the novel betacytorhabdoviruses identified in this study and those already known, showed some variability, mainly in the length of the intergenic spacer (Table 5).

Pairwise aa sequence identity values between each of the L proteins of the 39 novel viruses and those from known betacytorhabdoviruses varied significantly, ranging between 27% and 80.06% (Table S2), while the L protein identity for variants of the same virus ranged between 93.47% and 99.29% (Table S2). The highest L protein aa sequence identity with those cytorhabdoviruses proposed to be classified as betacytorhaboviruses and gammacytorhabdoviruses was 33.84% (Table S2).

Phylogenetic analysis based on L protein aa sequences showed that the 39 novel viruses grouped with 20 known cytorhabdoviruses in a distinctive major group that we named betacytorhabdoviruses (Figure 1). Within this distinct group of 59 viruses, several evolutionary clades could be distinguished (Figure 3A). One clade grouped together all viruses that have a short G gene and share a similar genomic organization with no additional accessory ORFs in their genomes except for Yerba mate virus A, which clustered basal to this clade (Figure 3). Another clade grouped together viruses with two accessory genes located between the P and M genes, exemplified by Sesamum betacytorhabdovirus 1 (Figure 3). Several other clades that grouped together viruses with a similar genomic organization were also observed (Figure 3). However, other clusters grouped together viruses with diverse genomic organizations (Figure 3). A similar topology was observed in the phylogenetic tree based on the N protein aa sequences (Figure S1).

### 3.4. Genus Gammacytorhabdovirus

**Figure 4:**
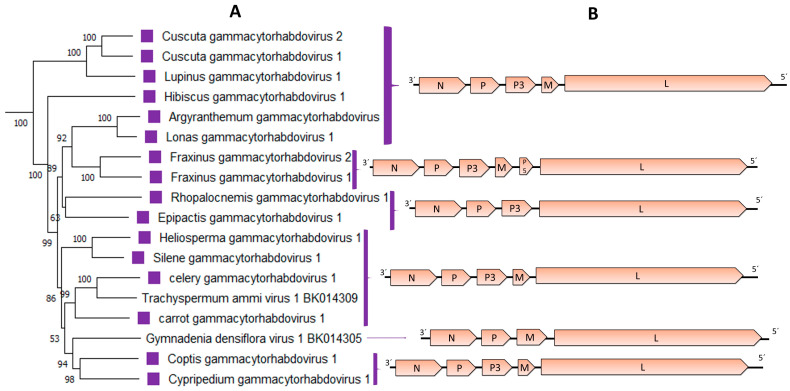
(**A**): An inset of the maximum-likelihood phylogenetic tree shown in Figure 1 was cropped to show those viruses included in the proposed genus *Gammacytorhabdovirus*. The viruses identified in this study are noted with violet squares. (**B**): genomic organization of the viral sequences used in the phylogeny.

The full-length coding regions of 16 novel putative gammacytorhabdoviruses were assembled in this study (Table 3), bringing the number of potential members of this proposed genus to 18, by the inclusion of two previously reported cytorhabdoviruses (Figure 4A). The newly identified viruses were tentatively associated with 15 plant host species and the fungus *Hymenoscyphus fraxineus* (Table 3). Most of the host plants (12/15) were herbaceous dicots, while two were orchids, and one was a dicot tree (Table 3).

The genomic organization of the 16 gammacytorhabdoviruses is quite similar with only a few exceptions. A common feature of all newly identified gammacytorhabdoviruses is the absence of the G gene. Twelve viruses had five genes in the order 3′-N-P–P3-M-L-5′; while two viruses had four genes in the order 3′-N-P–P3-L-5′ and lacked the M gene. Interestingly, the two viruses associated with Fraxinus display a genomic organization 3′-N-P–P3-M-P5-L-5′, with six genes including a small ORF located between the M and L genes (Table 3, Figure 4B). The predicted P5 protein showed no hits when BlastP searches were carried out, but one transmembrane domain was identified. 

The consensus gene junction sequences of the novel gammacytorhabdoviruses identified in this study were highly similar and resembled those of the two previously reported phylogenetically related cytorhabdoviruses (Table 5). 

Pairwise aa sequence identity values between each L protein of the 18 proposed gammacytorhabdoviruses varied significantly, ranging between 49% and 84% (Table S2). However, the highest sequence identity with those cytorhabdoviruses proposed to be classified as alphacytorhaboviruses and betacytorhabdoviruses was 33.4% (Table S2). 

The phylogenetic analysis based on the L protein aa sequence showed that the 16 novel viruses grouped with two known cytorhabdoviruses in a distinct group (Figure 1). Within this distinct group of 18 viruses, most of the clusters grouped together viruses with the same genome organization and/or type of hosts, such as both Fraxinus-associated viruses, the cluster that grouped the carrot, celery, and Trachyspermum-associated viruses, the cluster composed of the Heliosperma and Silene-associated viruses, or the cluster composed of the Argyranthemum and Lonas-associated viruses (Figure 4). Interestingly, the orchid-associated viruses (Cypripedium, Epipactis and Gymnadenia) did not share a similar genomic organization and did not cluster together (Figure 4). A similar topology was observed in the phylogenetic tree based on the N protein aa sequences (Figure S1).

### 3.5. Tri-Segmented Rhabdoviruses

Unexpectedly, the full-length coding regions of four novel viruses that consisted of three genome segments were also assembled (Table 4). The best hits of the L, N, P2, P3, and P4 proteins encoded by those four tri-segmented viruses were the cognate proteins encoded by Picris cytorhabdovirus 1 (PiCRV1) [20]. Two genome segments of this virus had previously been assembled, annotated, and deposited in GenBank (Accession # OL472127 and OL472128), but the assembled PiCRV1 N protein gene was significantly shorter than the N gene assembled for the four novel tri-segmented viruses. Consequently, we re-analyzed the SRA deposited by [20] and we were able to extend the sequence of the N gene, but also to assemble a previously unrecognized third segment. Thus, five tri-segmented rhabdo-like viruses, subsequently referred to as trirhaviruses, were identified from the SRA data analysis. We propose to rename the Picris-associated virus as Picris trirhavirus 1. 

**Figure 5:**
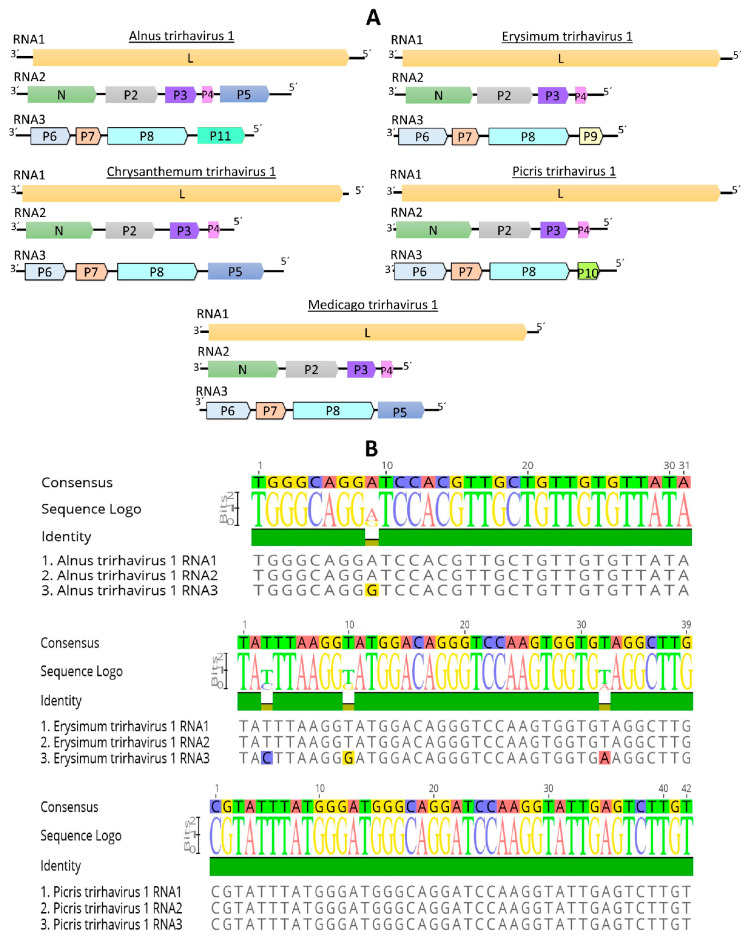
(**A**): genomic organization of all tri-segmented rhabdoviruses identified in this study (**B**): Alignment of the 5′ trailer sequence ends of the three RNA segments of Alnus-, Erysimum- and Picris-associated viruses. The predicted coding sequences are shown in arrowed rectangles. Colors indicate protein homologies.

RNA1 of all the tri-segmented viruses had one gene that encodes the L protein (Table 4, Figure 5A). RNA2 of four of these viruses had four genes in the order 3′-N-P2-P3-P4-5′ while one virus has five genes in its RNA2 in the order 3′-N-P2-P3-P4-P5-5′ (Table 4, Figure 5A). RNA3 of all tri-segmented viruses had 4 genes, where the first three encoded proteins, named as P6, P7 and P8, are homologous and syntenic to each other. The protein encoded at the 5′ end of segment 3 in the Chrysantheum and Medicago tri-segmented viruses is homologous to the P5 protein identified in the Alnus tri-segmented virus genome, while the proteins encoded in this position in the Erysimum, Picris and Alnus tri-segmented viruses are not homologous neither to P5 nor to each other, thus named as P9, P10 and P11, respectively (Table 4, Figure 5A).

One interesting feature discovered when the RNA segment ends of the Alnus, Erysmum and Picris tri-segmented viruses were analyzed, is that the 30 to 40 nucleotides located at the end of the 5′trailer of each one of the segments are 99% to 100% identical (Figure 5B). BlastP searches of each encoded protein showed that the L protein sequence of all tri-segmented rhabdoviruses was more similar to the L protein of cytorhabdoviruses than to the L protein of any other rhabdovirus. On the other hand, for every tri-segmented virus, the N protein best hits were the N proteins encoded by varicosaviruses or nucleorhabdoviruses. No similarity hits were found for P2, P3, P4, P6, P7, P8, P9, P10 or P11 in databases even with relaxed parameters. Strikingly, the P5 proteins showed hits against the putative silencing suppressor protein encoded by emaraviruses (family *Fimoviridae*) (Table 4). A signal P was predicted in each P2 and P5 proteins, while transmembrane domains were predicted in each P4 and P8 proteins. However, no conserved domains were predicted in any of the other viral proteins.

The consensus gene junction sequences of the tri-segmented rhabdoviruses are highly similar and like those previously reported for cytorhabdoviruses proposed to be classified as alphacytorhabdoviruses (Table 5).

Pairwise aa sequence identity values between each of the L proteins of the five tri-segmented viruses did not vary significantly, ranging between 55% and 66% (Table S2). On the other hand, the highest sequence identity with those cytorhabdoviruses proposed to be classified as alphacytorhaboviruses, betacytorhabdoviruses or gammacytorhabdoviruses was only 32% (Table S2). The highest sequence identity of trirhaviruses with the alpha-, beta- and gammanucleorhabdoviruses was 28.5%, and with varicosaviruses and gymnorhaviruses, the highest sequence identity was 28.6% and 27.5%, respectively.

The phylogenetic analysis based on deduced L protein aa sequences placed all tri-segmented rhabdoviruses into a distinct clade which is basal to all cytorhabdoviruses (Figure 1). The Alnus and Chrysantheum tri-segmented viruses grouped together (Figure 1), and these viruses are also the most similar in pairwise sequence identity values of their L proteins, but their RNA2 genomic organization is different (Figure 5A). The second cluster included the Erysmum, Medicago and Picris tri-segmented viruses (Figure 1), which have a similar genomic organization (Figure 5A). On the other hand, the phylogenetic tree based on deduced N protein aa sequences placed all tri-segmented rhabdoviruses into a distinct clade which is basal to all plant rhabdoviruses (Figure S1).

## 4. Discussion

### 4.1. Discovery of Novel Cytorhabdo-Like Viruses Expands Their Diversity and Evolutionary History

In the last few years, several novel cytorhabdoviruses that do not induce visible disease symptoms have been reported in HTS studies [91,92,93,94,95,96,97,98,99,100,101]. Moreover, many novel cytorhabdoviruses were identified when metatranscriptomic data publicly available at the Transcriptome Shotgun Assembly (TSA) sequence databases was mined [9]. On the other hand, the NCBI-SRA database, where many cytorhabdo-like virus sequences are likely hidden, remains significantly underexplored. This is because, traditionally, viruses were not expected to be present in sequence libraries of non-symptomatic plants. Nevertheless, the development of the Serratus tool [6] has greatly facilitated the exploration of the SRA database, which otherwise would be tedious and time-consuming, allowing us to carry out the most extensive search to date for cytorhabdovirus-like sequences. This substantial in silico directed search resulted in the identification and assembly of the full coding regions of 93 novel putative cytorhabdovirus members, representing a 1.7-fold increase in the known cytorhabdoviruses. The phylogenetic relationships, as well as the genomic features of the now expanded number of known cytorhabdoviruses, provide strong support for splitting the genus *Cytorhabdovirus* to establish three genera that we propose to name as *Alphacytorhabdovirus*, *Betacytorhabdovirus* and *Gammacytorhabdovirus*. However, the major highlight of our data mining efforts was the first-ever identification of rhabdoviruses with a tri-segmented genome. Thus, our findings clearly highlight the significance of data-driven virus discovery to increase our understanding of the genomic diversity, evolutionary trajectory, and singularity of the rhabdoviruses.

### 4.2. Proposed New Genus Alphacytorhabdovirus

The full-length coding regions of 38 novel alphacytorhabdoviruses were assembled in this study. Most of the associated host plants were herbaceous dicots (69% of the assigned hosts), in line with previous findings as 90% of the previously identified alphacytorhabdoviruses were also associated with herbaceous dicots. Thus, these viruses likely have a host adaptation trajectory leading to preferentially infecting herbaceous dicots during their evolution. The assigned hosts of six of the newly identified alphacytorhabdoviruses were monocots, and represent the first alphacytorhabdoviruses associated with monocots hosts. No apparent concordant evolutionary history with their plant hosts was observed for the monocot-infecting viruses, like what was previously reported for invertebrate and vertebrate rhabdoviruses [102]. Furthermore, one newly identified virus was associated with a wetland metagenome study, but even after extensive assessment of the corresponding libraries we were not able to clearly assign a host to this virus.

All but one alphacytorhabdovirus identified so far had at least the six basic plant rhabdovirus genes N, P, P3, M, G and L reported for cytorhabdoviruses [17]. The exception was one virus associated with the host Pogostemon, known as “patchouly chlorosis-associated cytorhabdovirus”, which was found to have a truncated G gene. It was speculated that this truncation may be linked to the fact that patchouli plants are primarily propagated vegetatively and may not require a functional G protein [94]. One distinctive feature of alphacytorhabdoviruses is the presence of an overlapping ORF within the P-encoding ORF, named P’ in most of their proposed members (65/71). At least one transmembrane domain was identified in each P′ protein predicted in the genomes of the alphacytorhabdoviruses assembled in this study. This is consistent with what has been previously reported for cytorhabdoviruses, where at least one transmembrane domain was identified in every P′ protein [9]. Hence, it could be speculated that this protein serves a membrane-associated function. Nevertheless, additional research should be directed toward the functional characterization of this intriguing protein. Moreover, 42/71 alphacytorhabdoviruses have an accessory ORF between the G and L genes. The encoded small protein contains transmembrane domains, and it was speculated that it may have membrane-associated functions similar to viroporins of vertebrate rhabdoviruses [9]. Other accessory ORFs were also detected in only two alphacytorhabdoviruses identified in this study. One of them, named P4, was located between the P3 and M genes in one virus and another one, dubbed P8, was found between the L gene and the 5′ trailer. No significant hits were found for P4 or P8 when BlastP searches were carried out, and no conserved domains were identified in these proteins. Another small accessory ORF, named P7, was previously reported to be located between the P6 and L genes of strawberry virus 1 [103]. Neither prediction of functional domains nor BLASTP searches against nonredundant GenBank database returned any significant hits [103]. Thus, further studies should be focused on the functional characterization of the P4, P7 and P8 proteins to gain knowledge about their potential roles. 

The consensus gene junction sequences among the alphacytorhabdoviruses are highly similar, likely indicating a common evolutionary history for this group of viruses. The nt sequence identity between the genomes of alphacytorhabdoviruses varied significantly ranging from 36% to 86%. This suggests that there may be still an unknown amount of “virus dark matter” within some clusters of the alphacytorhabdoviruses space worth exploring, which may contain some yet-to-be-discovered alphacytorhabdoviruses. Moreover, the highest sequence identity with those viruses not classified as potential alphacytorhabdoviruses is very low, which is common among plant rhabdoviruses, which are characterized by a high level of diversity in both genome sequence and organization [15]. On the other hand, when we analyzed the diversity between variants of viruses which are likely members of the same species, the sequence identity ranged from 89% to 96%.

Among all plant rhabdoviruses studied so far, there is a strong correlation between phylogenetic relationships and vector types [17]. Many members grouped within the alphacytorhabdoviruses have been shown to be aphid-transmitted [17,99], except for patchouly chlorosis-associated cytorhabdovirus, which was speculated to be vertically transmitted [94]. We, therefore, predict that the novel alphacytorhabdoviruses described here are likely aphid-transmitted. In support of this, the Triticum-associated virus was also found in a sequencing library of the aphid *Sitobium avenae*, thus providing some evidence for aphids as potential vectors of the newly identified alphacytorhabdoviruses. 

The observed phylogenetic relationships suggest a common evolutionary history for alphacytorhabdoviruses, with four major clades observed. All viruses in the clade including the well-studied lettuce necrotic yellows virus do not have an accessory ORF between the G and L genes; thus, these viruses may represent the ancestral clade within the alphacytorhabdoviruses. In another clade, all members but two had an accessory ORF between the G and L genes; therefore, these viruses may have evolved from an ancestor that already had that ORF, which is absent in the patchouly chlorosis-associated cytorhabdovirus and primula alphacytohabdovirus 2 genomes, while strawberry virus 1 acquired another accessory ORF during its evolution. In another clade, there are two major clusters, one that includes viruses that likely evolved from the ancestral ancestor, while the other cluster showed a more complex evolutionary history including members with distinct numbers of genes within their genomes. The fourth clade also showed a more complex evolutionary history because it included members with distinct genomic organizations, where many viruses acquired accessory ORFs during their evolution, mostly in the position between the G and L genes.

We propose to classify this group of evolutionary-related viruses into a novel genus within the family *Rhabdoviridae*, subfamily *Betarhabdovirinae* for which we suggest the name “*Alphacytorhabdovirus*”. Based on the phylogenetic insights and the observed genetic distance of the newly identified viruses we tentatively propose 86% aa sequence identity of the L protein as the threshold for species demarcation in this newly proposed genus which will include 72 members, for which the complete coding-sequence is available.

### 4.3. Proposed New Genus Betacytorhabdovirus

The full-length coding regions of 39 novel betacytorhabdoviruses were assembled in this study. Interestingly, half of the associated hosts were woody dicots, while 35% (7/20) of the previously identified cytorhabdoviruses of this group are also associated with woody dicots. Thus, many betacytorhabdoviruses likely infect woody dicots, which may be a distinctive feature of this group of viruses. Most of the monocot-infecting betacytorhabdoviruses grouped together suggesting a shared co-divergence for these viruses. Two newly identified betacytorhabdoviruses which clustered with monocot-infecting viruses were associated with a peat soil metagenome study. Therefore, it is tempting to speculate that monocots could be associated with these viruses. 

The genomic organization of the betacytorhabdoviruses is quite diverse, with 16 distinct genomic organizations discernable among its 59 putative members. Almost a quarter (14/59) of betacytorhabdoviruses lacked additional accessory genes and had at least the six basic genes N, P, P3, M, G and L reported for cytorhabdoviruses [17]. Nevertheless, 12 betacytorhabdoviruses had a shorter G gene. Four viruses lacked the G gene altogether, while one also lacked the M gene. The G protein was found to be essential for virus acquisition by arthropod vectors [15], but it is not essential for replication and systemic movement [104]. Some isolates of the betacytorhabdovirus citrus-associated rhabdovirus were recently shown to have a defective G gene, thus it was speculated that the lack of a functional G gene could provide an evolutionary advantage in fruit trees that are propagated artificially by asexual modes, such as cutting and grafting [105]. Moreover, the recently identified Rudbeckia virus 1, which was identified in Rudbeckia seeds, lacked the G gene [96]. Thus, it was predicted to be vertically transmitted by seeds without the help of a vector which may have favored the loss of the G gene during its evolution [96]. Hence, one might be inclined to speculate that viruses lacking the G gene or having a shorter G gene could potentially undergo vertical transmission. Furthermore, infections with viruses that lack the M gene have been reported to be asymptomatic [106], which is additional evidence supporting vertical transmission of the virus. Moreover, it has been shown, using a nucleorhabdovirus as a model, that cooperative M-G interactions are needed for some of the functions that involve the M protein [107]. Thus, perhaps in those viruses that lack the G gene, the M gene could become dispensable and may be prone to be lost during evolution like in the Cypripedium-associated virus, which lacks both genes. Further studies should experimentally assess these conjectures. Moreover, many betacytorhabdoviruses (27/59) have an accessory ORF between the G and L genes. This small protein has transmembrane domains, and it was speculated that it may have membrane-associated functions similar to viroporins in vertebrate rhabdoviruses [9]. Several other accessory ORFs were also identified in betacytorhabdoviruses reported in this study suggesting a complex evolutionary history where many members acquired additional ORFs during adaptation to their hosts. Four betacytorhabdoviruses had an accessory ORF between the L gene and the 5′trailer. Ten betacytorhabdoviruses had two ORFs between the P and M genes, while three others had four ORFs between these genes [17]. One of these ORFs encodes the putative cell-to-cell movement protein, which is named P3 in all but the Yerba mate virus A, where this protein is named P4 [91]. The other accessory proteins are named P4, P5 and P6. The P4 protein encoded by the newly identified Sesamum virus 1, and the one encoded by the known Bemisia tabaci associated virus, Cucurbit cytorhabdovirus 1, Yerba mate chlorosis associated virus, soybean blotchy mosaic virus, papaya virus E and Aristolochia-associated cytorhabdovirus; as well as the P5 protein encoded by barley yellow striate mosaic virus (BYSMV) and maize yellow striate virus, are small proteins (70–80aa) with a predicted transmembrane domain, suggesting a membrane association function. Indeed, the BYSMV P5 was shown to be targeted to the endoplasmic reticulum and it was suggested that the features of this protein are reminiscent of the small hydrophobic proteins of tupaia rhabdovirus [108]. Two viruses had two additional accessory ORFs between the viroporin-like protein gene and the L gene, while another virus had one accessory ORF in that position. An overlapping ORF within the one encoding the viroporin-like protein was found in a cytorhabdovirus associated with the Linden tree *Tillia cordata* [95]. In the Justicia-associated virus, an accessory ORF was found between the N and P genes. An accessory ORF located in this position has been described for some alphanucleorhabdoviruses [17], but Justicia-associated virus appears to be the first cytorhabdovirus with an ORF in this position. For the above accessory ORFs, except for the viroporin-like proteins and the P4/P5 proteins, BlastP results were orphans, no known signals, or domains present, and no clues towards their putative (conserved?) function were found. Thus, further studies should be focused on the functional characterization of these proteins to gain essential knowledge regarding the proteome of the accessory ORFs of betacytorhabdoviruses.

The consensus gene junction sequences of the novel and previously reported betacytorhabdoviruses showed some variability, but there appears to be a correlation with the phylogenetic relationships thus supporting a common evolutionary history for these viruses.

When we analyzed the diversity between variants of viruses that likely belong to the same species, nt sequence identity ranged from 93.5% to 99%. On the other hand, the pairwise aa sequence identity among betacytorhabdoviruses L protein showed a great variation ranging between 27% and 80% which suggests that there may be many more betacytorhabdoviruses yet to be discovered. Moreover, the sequence identity with those viruses not classified as potential betacytorhabdoviruses is very low (<33.9%), which is a common feature among plant rhabdoviruses, that are characterized by a high level of diversity in both genome sequence and organization [15]. Furthermore, this high sequence diversity coupled with the distinct genomic architecture displayed by betacytorhabdoviruses, and the complex evolutionary history as shown in the phylogenetic analyses may set the foundation to further split this proposed genus in the future once additional members can be identified. 

Among all plant rhabdoviruses studied so far, there is a strong correlation between phylogenetic relationships and vector types [17]. Some betacytorhabdoviruses have been shown to be transmitted by planthoppers, others by leafhoppers and others by whiteflies [17]. We, therefore, predict that the potential vectors of the novel betacytorhabdoviruses may be whiteflies, planthoppers, leafhoppers and likely non-aphid arthropods, like psyllids. Those betacytorhabdoviruses that lack the G gene or with a shorter G gene are likely vertically transmitted.

The phylogenetic analysis of betacytorhabdoviruses revealed several major clades suggesting a complex evolutionary history. One clade grouped together all viruses with a short G gene and without accessory genes, except for Yerba mate virus A. These viruses, with one exception, are associated with woody dicots; therefore, it is tempting to speculate that the ancestor virus was adapted to infect woody plants and that Yerba mate virus A acquired an additional ORF during its host adaptation. Another clade includes most of the viruses with two genes between the P and M genes but no accessory ORFs in other positions in their genomes likely indicating that they share the same ancestor. Certain clusters encompassed viruses that share common genomic organization, while other clusters featured viruses with unique genomic structures. An example of this diversity can be seen in the cluster that includes Cypripedium- and Mango-infecting viruses. This highlights the intricate evolutionary history of most betacytorhabdoviruses, with many of them acquiring additional genes during their evolution. Notably, these gene acquisitions were primarily concentrated between the P and M genes or between the G and L genes. 

Based on the phylogenetic insights and the observed genetic distances of the newly identified viruses we tentatively propose an aa sequence identity of 82% in the L protein as the threshold for species demarcation in this newly proposed genus which will include 59 members, for which the complete coding-sequence is available.

### 4.4. Proposed New Genus Gammacytorhabdovirus

The full-length coding regions of 16 novel gammacytorhabdoviruses were assembled in this study, and most of the associated host plants were herbaceous dicots. Three viruses were linked to orchids, while two were associated with the woody tree Fraxinus but, interestingly, one of them was identified in a library of the fungal pathogen (*Hymenoscyphus fraxineus*) sampled from this woody tree.

The common feature of all 18 gammacytorhabdoviruses identified so far (16 in this study and two in [9]) is the lack of a G gene in their genome. The G gene was shown, using as a model an infectious clone of the Sonchus yellow net virus, to be not essential for replication and systemic movement [104]. Those two viruses associated with Fraxinus, have an additional ORF between the M and L genes, which we named P5. Interestingly, transmembrane domains were predicted for P5 suggesting a membrane-associated function for this protein that has a similar size to cytorhabdovirus viroporin-like proteins, which also have transmembrane domains [9]. Nevertheless, no distant hits with viroporin-like proteins were found when we used HHblits on the predicted P5 protein. One previously identified gammacytorhabdovirus, associated with the orchid Gymandenia, lacks not only the G gene but also the P3 gene. Thus, how GymDenV1 moves from cell to cell remains to be unraveled, but no cell-to-cell movement protein has either been identified in the fungi-transmitted varicosaviruses [9]. Strikingly, two gammacytorhabdoviruses identified in this study, one associated with the orchid *Epipactis* and the other with the parasitic plant *Rhopalocnemis*, do not have M and G genes. Previous studies have assumed that the nucleocapsid core (NC) proteins N, P and L are essential for virus replication and transcription and that the M protein is required for condensation of the core during virion assembly [15]. M protein appears to be required for the long-distance movement of the virus within the plant [104], and an infectious clone of a plant rhabdovirus lacking the M gene displayed reduced infectivity, a vasculature-confined tissue tropism and no visible symptoms [106]. Moreover, it has been shown, using a nucleorhabdovirus as a model, that cooperative M-G interactions are needed for some of the functions that involve the M protein [107]. Thus, it is tempting to speculate that in those viruses that lack the G gene, the M gene could be dispensable, and may have been lost during the evolution of the *Epipactis-* and *Rhopalocnemis*-associated viruses. 

It has been suggested that the fungi-transmitted varicosaviruses, which do not encode a G protein [10], may have originated through trans-kingdom horizontal gene transfer events between fungi and plants, adapting specifically to a plant-based lifestyle [5]. The absence of the G gene, coupled with the detection of one of the recently identified gammacytorhabdoviruses in a fungal library, raises the possibility that these viruses might be transmitted by a fungal vector rather than by arthropods, as is commonly observed in viruses classified as alpha- and betacytorhabdoviruses. This serves as another distinguishing characteristic of the gammacytorhabdoviruses. Thus, further studies should focus on the potential vector and the mode of transmission of gammacytorhabdoviruses. 

Another distinctive feature of gammacytorhabdoviruses is that the intergenic spacer of their gene junctions starts with an A instead of a typical G, like all other plant rhabdoviruses [9,10] suggesting a unique evolutionary history of these viruses. 

The nt sequence identity among gammacytorhabdoviruses showed a high variation ranging between 49% and 84%. Moreover, the pairwise aa sequence identity with the L protein of those viruses not classified as potential gammacytorhabdoviruses is very low (<33.5%), suggesting unknown gammacytorhabdovirus diversity is yet to be discovered. 

Interestingly, the three orchid-associated viruses (*Cypripedium*, *Gymnadenia*, and *Epipactis*) have different genomic organization, where one virus lacks the G gene, another does not encode the G and M proteins, while the third does not have the P3 and G genes. Moreover, they are not grouped together in the phylogenetic tree, thus they likely did not share a common evolutionary history. On the other hand, most of the viruses infecting herbaceous dicot hosts, as well as those associated with woody trees, clustered together according to the host family, suggesting a shared host-virus co-divergence in those clades. 

We propose to classify this group of evolutionary-related viruses sharing the lack of the G gene in their genomes as a distinctive feature, into a novel genus within the family *Rhabdoviridae*, subfamily *Betarhabdovirinae* for which we suggest the name “*Gammacytorhabdovirus*”. Based on the phylogenetic insights and the observed genetic distance of the newly identified viruses we tentatively propose an aa sequence identity of 85% in the L protein as the threshold for species demarcation in this newly proposed genus which will include 18 members, for which the complete coding-sequences are available.

### 4.5. Tri-Segmented Rhabdoviruses

All rhabdoviruses identified to date have unsegmented genomes, except for the dichorhaviruses and most varicosaviruses which have bi-segmented genomes [10,15]. Unexpectedly, five novel viruses with tri-segmented genomes were identified in this study, including the corrected full-length coding genome segments of the previously reported PiCRV1 [20]. 

RNA1 of all these tri-segmented viruses had only one gene that encodes the L protein, which is similar to the bi-segmented rhabdoviruses where the L protein is the only gene product present in the varicosaviruses RNA1, and in the dichorhaviruses RNA2 [10,17]. RNA2 of four of the viruses has four genes, while the Alnus tri-segmented virus has five genes. Five genes are present in RNA1 of dichorhaviruses [17], while three to five genes are present in RNA2 of varicosaviruses [10], with the N gene being the only orthologous gene between them. RNA3 of all tri-segmented viruses has four genes, where the first three encoded proteins are homologous. The protein encoded at the end of this segment in the Chrysanthemum and Medicago tri-segmented viruses is homologous to P5 on RNA2 of the Alnus tri-segmented virus genome, while the proteins located in this position in the Erysimum, Picris and Alnus tri-segmented viruses are unique. This genomic organization is unique among rhabdoviruses [15,16] and represents the first known tri-segmented rhabdovirus genomes. Other segmented negative-sense RNA viruses (NSR), belonging to the order *Bunyavirales*, have one or two genes on each RNA segment. Thus, the genomic organization of the tri-segmented rhabdoviruses identified in this study is likely distinctive among NSR viruses.

The ends of the 5′ trailer region of all genome segments are conserved in the tri-segmented viruses identified in our study. A similar feature is observed in the other segmented rhabdoviruses and NSR viruses, which may be linked to RNA-dependent RNA polymerase-mediated recognition for replication [15].

BlastP searches of the L protein encoded on RNA1 of all identified tri-segmented viruses showed that this protein is most closely related to the L protein encoded by cytorhabdoviruses, while the best hits for the N protein were the N proteins coded by varicosaviruses or nucleorhabdoviruses. This suggests that these two proteins, which are located on different RNA segments, have distinct evolutionary histories. On the other hand, no hits were found for P2, P3, P4, P6, P7, P8, P9, P10 or P11. Strikingly, P5 showed hits against the putative RNA silencing suppressor protein encoded by emaraviruses (family *Fimoviridae*), plant viruses with segmented, linear, single-stranded, negative-sense genomes [109] in the order *Bunyavirales* [110], while rhabdoviruses are classified in the order *Mononegavirales* [16]. Viral RNA silencing suppressors are required for systemic infection of the plant host and the presence of these proteins suggests that the tri-segmented viruses detected here are plant-associated [5]. 

A signal peptide was predicted in each P5 protein, which may be associated with its RNA silencing suppressor function, and in each P2 protein. Interestingly, a signal peptide is present in the movement protein (MP) encoded by emaraviruses [110], but none were identified in the MP encoded by plant rhabdoviruses [9], and no distant hits with any MP were found when using HHblits on the P2. Transmembrane domains were predicted in each P4 and P8 proteins suggesting a membrane-associated function for these proteins. P4 size is similar to that reported for cytorhabdovirus viroporin-like proteins, which also have transmembrane domains [9] while P8 size is similar to that reported for the glycoprotein encoded by plant rhabdoviruses [9], but no distant hit with any viroporin-like protein or glycoprotein was found using HHblits. No conserved domains were found in the other coded proteins. Thus, further studies should be focused on the functional characterization of the P2, P3, P4, P6, P7, P8, P9, P10 and P11 proteins to gain fundamental insights about the proteome of the tri-segmented viruses beyond the N, L and P5 proteins.

The novel tri-segmented viruses also resemble rhabdoviruses in possessing similar conserved gene junctions that are also highly similar to those present in the alphacytorhabdoviruses.

The pairwise aa sequence identities between the L proteins of all the tri-segmented viruses were not low at all, ranging between 55% and 66%, which may suggest that tri-segmented rhabdoviruses are evolutionarily younger than unsegmented ones. 

The phylogenetic analysis based on deduced L protein aa sequences placed all tri-segmented viruses into a distinct clade within the plant rhabdoviruses that is grouped with the cytorhabdoviruses rather than with varicosaviruses or nucleorhabdoviruses, whereas the phylogenetic tree based on the N protein placed the tri-segmented viruses in a clade which is basal to all plant rhabdoviruses. The complex evolutionary history of this divergent group of viruses suggests that they share a unique evolutionary history among rhabdoviruses. It is tempting to speculate that the RNA segment encoding the L protein evolved from a cytorhabdovirus ancestor, while the RNA segment encoding the N protein may have evolved from a rhabdovirus ancestor of all tri-segmented viruses, except for the Alnus-associated virus. The presence of an emaravirus-related protein in its RNA2 segment, as well as in the RNA3 segment of the Chrysanthemum- and Medicago-associated tri-segmented viruses leads us to speculate that these segments may have emerged from the recombination of a negative-sense rhabdovirus ancestor and an emaravirus. On the other hand, the RNA3 segment of the viruses from Alnus, Erysimum and Picris may have evolved from a segmented negative-sense rhabdovirus ancestor.

Taken together, these tri-segmented viruses may be taxonomically classified in a novel genus within the family *Rhabdoviridae*, subfamily *Betarhabdovirinae* for which we suggest the name “*Trirhavirus*”. Based on the phylogenetic insights and the observed genetic distance of the newly identified viruses we tentatively propose an aa sequence identity of 80% in the L protein as threshold for species demarcation in this proposed genus. 

### 4.6. Strengths and Limitations of Sequence Discovery through Data Mining

As demonstrated previously by Bejerman and colleagues [10] and in this study with the Picris-associated virus, the independent validation through re-analyzes of the NCBI-SRA raw data of viruses assembled with unexpected genomes is important to enhance our comprehension and confidence in the genomic architecture of RNA viruses assembled via HTS data. However, the inability to revisit the original biological material for replication of results and verification of the assembled viral genome sequences is a significant weakness of the data mining approach in virus discovery. Moreover, potential issues such as contamination, low sequencing quality, spill-over, and other technical artifacts pose a risk of yielding false-positive detections, chimeric assemblies, or difficulties in accurately assigning host organisms. Therefore, researchers should be cautious when scrutinizing publicly available SRA data for virus detection. To bolster and complement such results, the acquisition of new RNAseq datasets from the predicted plant hosts is strongly recommended. Furthermore, the absence of a directed strategy for verifying genomic segment termini, such as the use of Rapid Amplification of cDNA Ends (RACE), presents challenges in determining bona fide RNA virus ends, especially considering the conserved functional and structural cues observed in rhabdoviruses [15]. Despite these limitations, certain aspects of our virus discovery strategy can help mitigate some of these challenges and provide additional evidence for identification. For example, when the same putative virus is consistently identified in multiple independent libraries originating from the same plant host, when there is substantial coverage of virus-related reads when multiple RNA segments of the virus are detected within a single library, or when different viral strains are identified in plants that are closely related in terms of their evolutionary history. Nonetheless, it is essential to acknowledge that associations and detections provided in this work and other data-driven studies should be viewed as preliminary and should be complemented through subsequent studies.

## 5. Conclusions

In conclusion, this study underlines the significance of analyzing SRA public data as a valuable tool, not only for expediting the discovery of novel viruses but also for gaining insights into their evolutionary history and enhancing virus classification. Through this approach, we conducted a search for hidden cytorhabdovirus-like sequences, which significantly expanded the number of putative cytorhabdoviruses. It also allowed us to unequivocally split this group of viruses into three genera resulting in the most comprehensive cytorhabdoviruses phylogeny to date, highlighting their diversity and complex evolutionary dynamics. The major finding of our study was the first-ever identification of tri-segmented rhabdoviruses, which shows the extensive plasticity inherent to the rhabdovirus genome organization including members with unique and intriguing evolutionary trajectories. Thus, future studies should explore various unresolved aspects of these viruses, such as potential symptoms, vertical transmission, and possible vectors.

## Figures and Tables

**Table 1:** Summary of novel alphacytorhabdoviruses identified from plant RNA-seq data available on NCBI.

Plant Host	Taxa/ Family	Virus Name/ Abbreviation	Bioproject ID/ Data Citation	Length (nt)/Coverage	Accession Number	Protein ID	Length (aa)	Highest Scoring Virus-Protein/*E*-Value/Query Coverage %/Identity % (Blast P)
Greater burdock (*Arctium lappa*)	Dicot/*Asteraceae*	Arctium alphacytorhabdovirus 1/ ArcACRV1	PRJNA598011/ [21]	12768/310.2X	BK064262	N	450	WhIV5-N/0.0/91/63.07
						P	300	SaV1-P/1e-100/100/50.67
						P′	115	no hits
						P3	225	TrARV1-P3/6e-93/97/59.91
						M	182	CnV2-M/2e-54/98/47.49
						G	552	TrARV1-G/0.0/98/55.09
						L	2097	WhIV5-L/0.0/99/64.42
Silvery wormwood (*Artemisia argyi*)	Dicot/*Asteraceae*	Artemisia alphacytorhabdovirus 1/ ArtACRV1	PRJNA397671/ [22]	12978//55.94X	BK064263	N	454	LNYV-N/0.0/88/60.30
						P	315	LNYV-P/9e-91/93/50.68
						P3	307	LNYV-P3/2e-119/96/54.52
						M	184	LNYV-M/2e-48/96/45.76
						G	552	LNYV-G/0.0/99/51.18
						L	2074	LNYV-L/0.0/99/68.25
Common wormwood (*Artemisia montana*)	Dicot/*Asteraceae*	Artemisia alphacytorhabdovirus 2/ ArtACRV2	PRJDB8414/ Kyoto University, Japan, unpublished	14344/82.73X	BK064264	N	479	RVCV-N/1e-175/98/51.95
						P	343	RCVC-P/1e-84/92/44.55
						P′	88	RVCV-P′/2e-10/64/49.12
						P3	241	RVCV-P3/2e-74/78/56.08
						M	182	RVCV-M/1e-49/83/50.66
						G	569	RVCV-G/0.0/92/55.51
						P6	64	RVCV-P6/3e-11/90/44.83
						L	2086	RVCV-L/0.0/99/64.72
Sievers wormwood (*Artemisia sieversiana*)	Dicot/*Asteraceae*	Artemisia alphacytorhabdovirus 3/ ArtACRV3	PRJNA834888/ [23]	14339/57.85X	BK064265	N	476	RVCV-N/2e-171/98/50.62
						P	342	RCVC-P/6e-82/93/43.69
						P′	103	RVCV-P′/3e-13/66/54.93
						P3	240	RVCV-P3/3e-75/82/52.40
						M	185	RVCV-M/1e-52/82/51.97
						G	571	RVCV-G/0.0/91/56.76
						P6	64	RVCV-P6/7e-14/100/48.44
						L	2089	RVCV-L/0.0/99/63.43
Desert broom (*Baccharis sarothroides*)	Dicot/*Asteraceae*	Baccharis alphacytorhabdovirus 1/ BacACRV1	PRJNA716650/ Romero, M., UNAM, Mexico, unpublished	13581/25.63X	BK064266	N	470	CCyV1-N/1e-153/87/52.68
						P	297	CCyV1-P/2e-56/98/40.74
						P′	85	no hits
						P3	337	StrV2-P3/4e-120/93/56.78
						M	168	CCyV1-M/5e-34/95/38.65
						G	550	CCyV1-G/1e-160/92/43.14
						L	2074	CCyV1-L/0.0/99/59.24
Large bittercress (*Cardamine amara*)	Dicot/*Brassicaceae*	Cardamine alphacytorhabdovirus 1/ CarACRV1	PRJDB4989/ [24]	13209/83.24X	BK064267	N	457	PaCRV1-N/0.0/99/72.35
						P	316	PaCRV1-P/5e-134/63.26
						P′	109	PaCRV1-P′/1e-21/48.11
						P3	224	PaCRV1-P3/8e-122/98/75.57
						M	164	PaCRV1-M/1e-89/100/73.78
						G	569	PaCRV1-G/0.0/95/75.64
						P6	70	PaCRV1-P6/5e-26/97/63.24
						L	2092	PaCRV1-L/0.0/100/78.30
Greater celandine (*Chelidonium majus*)	Dicot/*Papaveraceae*	Chelidonium alphacytorhabdovirus 1/ CheACRV1	PRJNA376854/ Zhao, L., Jinlin, China, unpublished	12148/68.31X	BK064268	N	415	TpVA-N/0.0/100/70.19
						P	325	TpVA-P/2e-146/100/63.38
						P′	71	no hits
						P3	200	TpVA-P3/8e-117/98/80.71
						M	169	TpVA-M/2e-73/92/69.43
						G	552	TpVA-G/0.0/98/71.72
						P6	66	GlLV1-P6/2e-14/100/57.58
						L	2072	TpVA-L/0.0/100/80.41
Indian chrysanthemum (*Chrysanthemum indicum*)	Dicot/*Asteraceae*	Chrysanthemum alphacytorhabdovirus 1/ ChrACRV1	PRJNA361213/ [25]	12715/71.27X	BK064269	N	448	WhIV5-N/0.0/99/60.22
						P	301	SaV1-P/4e-100/99/51
						P′	140	no hits
						P3	225	TrARV1-P3/1e-91/97/57.92
						M	19	CnV2-M/6e-50/91/45.60
						G	549	SaV1-G/0.0/100/53.42
						L	2097	WhIV5-L/0.0/99/63.89
Bear corn (*Conopholis americana*)	Dicot/*Orobanchaceae*	Conopholis alphacytorhabdovirus 1/ ConACRV1	PRJEB21674/ 1000 Plant (1KP) Transcriptomes Initiative, Unpublished	13083/178.6X	BK064270	N	467	CCyV1-N/1e-157/94/49.1
						P	299	CCyV1-P/4e-70/100/42.35
						P′	87	no hits
						P3	328	StrV2-P3/3e-119/83/60.58
						M	166	BCRV2-M/7e-32/93/38.06
						G	546	CCyV1-G/8e-151/99/40.26
						L	2076	CCyV1-L/0.0/98/58.75
Cardoon (*Cynara cardunculus*)	Dicot/*Asteraceae*	Cynara alphacytorhabdovirus 1/ CynACRV1	PRJNA590905/ [26]	13726/33.22X	BK064271	N	472	TCRV1/0.0/100/80.08
						P	311	TCRV1-P/3e-153/100/69.97
						P′	132	TCRV1-P′/1e-23/75/53.54
						P3	350	TCRV1-P3/0.0/99/80
						M	179	TCRV1-M/3e-100/100/78.77
						G	562	TCRV1-G/0.0/98/70.40
						L	2144	TCRV1-L/0.0/98/83.25
Fischer´s spurge (*Euphorbia fischeriana*)	Dicot/*Euphorbiaceae*	Euphorbia alphacytorhabdovirus 1/ EupACRV1	PRJNA693977/ [27]	13713/109.3X	BK064272	N	451	PeVA-N/0.0/95/68.41
						P	330	PeVA-P/2e-100/99/53.62
						P3	223	PeVA-P3/1e-84/100/55.61
						P4	130	no hits
						M	184	PeVA-M/5e-57/96/48.88
						G	559	PeVA-G/0.0/95/68.35
						P7	41	no hits
						L	2089	PeVA-L/0.0/99/69.3
Tikoua fig (*Ficus tikoua*)	Dicot/*Moraceae*	Ficus alphacytorhabdovirus 1/ FicACRV1	PRJNA432314/ Bai, Y., Guiyang University, China, unpublished	13839/18.65X	BK064274	N	458	SCV-N/1e-140/97/48.80
						P	316	SCV-P/3e-52/88/36.51
						P′	84	no hits
						P3	228	SCV-P3/2e-75/100/50.43
						M	182	SCV-M/7e-43/82/48.67
						G	563	SCV-G/0.0/94/50.46
						P6	69	ADV-P6/3e-09/98/42.03
						L	2097	SCV-L/0.0/99/60.22
Garlic (*Allium sativum*)	Monocot/*Amaryllidaceae*	Garlic alphacytorhabdovirus 1/ GarACRV1	PRJNA772184/ Liu, T., IBFC, China, unpublished	13400/76.43X	BK064275	N	468	LNYV-N/3e-160/93/53.17
						P	298	TpVB-P/4e-53/94/34.80
						P3	329	StrV2-P3/5e-126/98/58.28
						M	174	LNYV-M/3e-35/97/40.94
						G	554	TpVB-G/5e-175/98/44.97
						L	2075	LYMV-L/0.0/99/58.43
Herb bennet (*Geum urbanum*)	Dicot/*Rosaceae*	Geum alphacytorhabdovirus 1/ GeuACRV1	PRJEB23354/ [28]	12756/12.97X	BK064276	N	459	StrV2-N/0.0/98/73.57
						P	295	StrV2-P/7e-128/100/60.34
						P′	98	StrV2-P′/3e-22/98/55.67
						P3	319	StrV2-P3/1e-171/100/74.22
						M	177	BCRV2-M/7e-78/92/68.29
						G	546	BCRV2-G/0.0/96/69.57
						L	2093	BCRV2-L/0.0/99/72.89
English ivy (*Hedera helix*)	Dicot/*Araliaceae*	Hedera alphacytorhabdovirus 1/ HedACRV1	PRJEB21674/ 1000 Plant (1KP) Transcriptomes Initiative, Unpublished	12588/11.88X	BK064277	N	459	StrV2-N/0.0/97/71.27
						P	295	StrV2-P/3e-128/100/61.02
						P′	98	StrV2-P′/2e-22/98/53.61
						P3	319	StrV2-P3/2e-171/100/74.30
						M	172	BCRV2-M/2e-78/99/65.50
						G	546	BCRV2-G/0.0/99/68.19
						L	2100	BCRV2-L/0.0/98/72.95
Plum-leaved holly (*Ilex asprella*)	Dicot/*Aquifoliaceae*	Ilex alphacytorhabdovirus 1/ IleACRV1	PRJNA736810/ [29]	14540/166.3X	BK064278	N	452	AcCV-N/0.0/99/56.87
						P	333	AcCV-P/1e-67/96/39.76
						P′	93	no hits
						P3	370	AcCV-P3/1e-130/99/52.49
						M	184	AcCV-1e-33/99/37.30
						G	553	AcCV-G/0.0/96/49.06
						P6	54	no hits
						L	2137	AcCV-L/0.0/98/60.77
						P8	134	no hits
Lucerne (*Medicago sativa*)	Dicot/*Fabaceae*	Medicago alphacytorhabdovirus 1/ MedACRV1	PRJNA644634/ [30]	13586/9.75X	BK064279	N	431	StrV1-N/0.0/97/60.48
						P	360	StrV1-1e-73/98/39.12
						P′	64	StrV1-P′/2e-13/100/54.69
						P3	224	StrV1-P3/7e-81/99/52.47
						M	191	StrV1-M/5e-43/83/44.65
						G	547	StrV1-G/0.0/99/60.44
						P6	80	StrV1-P6/6e-11/76/45.9
						L	2085	StrV1-L/0.0/99/68.21
Horse mint (*Mentha longifolia*)	Dicot/*Lamiaceae*	Mentha alphacytorhabdovirus 1/ MenACRV1	PRJNA779119/ Wang, B., Shaoguan University, unpublished	12387/56.72X	BK064280	N	422	TpVA-N/9e-163/100/54.14
						P	316	TpVA-/7e-45/96/32.18
						P′	101	no hits
						P3	195	TpVA-P3/6e-68/97/55.38
						M	163	GlLV1-M/6e38/95/45.16
						G	551	TpVA-G0.0/97/55.84
						P6	67	GlLV1-P6/4e-07/100/50.75
						L	2072	TpVA-L/0.0/99/62.64
Indian mulberry (*Morinda officinalis*)	Dicot/*Rubiaceae*	Morinda alphacytorhabdovirus 1_Mor/MorACRV1_Mor	PRJNA717096/ [31]	13023/25.34X	BK064281	N	463	CCyV1-N/0.0/99/56.87
						P	300	CCyV1-P/7e-68/98/42.71
						P′	81	no hits
						P3	346	BCRV2-P3/1e-115/76/59.7
						M	172	CCyV1-M/7e-29/92/37.58
						G	548	CCyV1-G/6e-171/93/45.14
						L	2075	CCyV1-L/0.0/99/59.16
Chinese holly (*Ilex cornuta*)	Dicot/*Aquifoliaceae*	Morinda alphacytorhabdovirus 1_Ile/MorACRV1_Ile	PRJNA399054/ [32]	12876/20.54X	BK064282	N	463	CCyV1-N/0.0/99/56.29
						P	300	CCyV1-P/1e-66/98/43.39
						P′	85	no hits
						P3	346	BCRV2-P3/2e-116/76/60.08
						M	184	CCyV1-M/2e-27/88/36.31
						G	549	CCyV1-G/7e-167/98/43.57
						L	2075	CCyV1-L/0.0/99/59.16
Oak (*Quercus robur*)	Dicot/*Fagaceae*	Oak alphacytorhabdovirus 1/ OakACRV1	PRJNA322128/ [33]	12817/21.81X	BK064283	N	453	LYMV-N/2e-109/93/40.47
						P	304	LYMV-P/1e-20/88/29.58
						P′	104	no hits
						P3	376	AscSyV1-P3/2e-46/66/35.97
						M	176	CCyV1-M/3e-06/59/28.57
						G	543	TrARV1-G/3e-7/98/30.16
						L	2078	BCRV2-L/0.0/99/45.57
Holy basil (*Ocimum tenuiflorum*)	Dicot/*Lamiaceae*	Ocimum alphacytorhabdovirus 1/ OciACRV1	PRJNA251328/ [34]	12478/24.87X	BK064284	N	425	KePCyV-N/0.0/99/76.65
						P	327	KePCyV-P/1e-116/99/54.41
						P′	93	no hits
						P3	205	KePCyV-P3/1e-105/99/70.53
						M	155	KePCyV-M/5e-58/98/56.21
						G	563	KePCyV-G/0.0/96/70.46
						P6	60	StrV1-P6/2e-08/83/50
						L	2078	KePCyV-L/0.0/99/77.36
Scented pelargonium (*Pelargonium* X *hybrid*)	Dicot/*Geraniaceae*	Pelargonium alphacytorhabdovirus 1/ PelACRV1	PRJNA883637/ Saint-Marcoux, D., Lyon University, France, unpublished	12332/91.55X	BK064285	N	413	TpVA-N/1e-164/98/56.23
						P	328	TpVA-P/2e-53/97/36.25
						P′	84	no hits
						P3	203	TpVA-P3/2e-91/96/68.02
						M	170	TpVA.M/5e-37/90/44.81
						G	552	TpVA-G/0.0/95/60.71
						P6	58	no hits
						L	2073	TpVA-L/0.0/99/63.7
Moso bamboo (*Phyllostachys edulis*)	Monocot/*Poaceae*	Phyllostachys alphacytorhabdovirus 1/ PhyACRV1	PRJNA350353/ [35]	12947/94.47X	BK064286	N	455	LNYV-N/2e-138/97/46.55
						P	296	LNYV-P/2e-44/93/37.28
						P′	83	no hits
						P3	328	StrV2-P3/7e-140/96/59.81
						M	193	LNYV-M/6e-28/90/34.27
						G	544	StrV2-G/8e-144/90/40.93
						L	2070	BCRV2-L/0.0/99/57.21
Peltate green dragon (*Pinellia peltata*)	Monocot/*Araceae*	Pinellia alphacytorhabdovirus 1/ PinACRV1	PRJNA623739/ [36]	13438/126.5X	BK064287	N	479	AscSyV1-N/4e-131/90/44.24
						P	305	DV1-P/8e-47/98/36.75
						P′	87	no hits
						P3	373	AscSyV1-P3/9e-105/89/47.51
						M	170	TCRV1-M/1e-05/88/31.21
						G	568	WhIV4/2e-134/95/36.73
						P6	61	no hits
						L	2106	WhIV4/0.0/99/48.13
Patchouli (*Pogostemom cablin*)	Dicot/*Lamiaceae*	Pogostemom alphacytorhabdovirus 1_Pog/ PogACRV1_Pog	PRJNA660501/ [37]	13171/250.8X	BK064288	N	462	CCyV1-N/0.0/99/55.17
						P	300	CCyV1-P/1e-69/96/40.79
						P′	81	no hits
						P3	347	StrV2-P3/3e-116/77/61.94
						M	179	CCyV1-M/3e-38/92/36.14
						G	557	CCyV1-G/1e-160/93/40.92
						L	2070	CCyV1-L/0.0/58.03
Black pepper (*Piper nigrum*)	Dicot/*Piperaceae*	Pogostemom alphacytorhabdovirus 1_ Pip/ PogACRV1_Pip	PRJNA580359/ [38]	13063/22.35X	BK064289	N	462	CCyV1-N/0.0/99/54.96
						P	300	CCyV1-P/3e-67/99/40.79
						P′	81	no hits
						P3	347	StrV2-P3/3e-116/77/62.31
						M	179	CCyV1-M/5e-39/87/39.74
						G	557	CCyV1-G/7e-164/91/42.88
						L	2070	CCyV1-L/0.0/58.75
Tropical soda apple (*Solanum viarum*)	Dicot/*Solanaceae*	Pogostemom alphacytorhabdovirus 1_Sol/ PogACRV1_Sol	PRJNA666394/ [39]	13138/26.34X	BK064290	N	462	CCyV1-N/3e-179/99/54.84
						P	300	CCyV1-P/5e-63/99/39.79
						P′	116	no hits
						P3	351	StrV2-P3/4e-115/76/61.57
						M	179	CCyV1-M/4e-40/87/40.38
						G	558	CCyV1-G/2e-157/95/40.98
						L	2070	CCyV1-L/0.0/58.31
Patchouli (*Pogostemom cablin*)	Dicot/*Lamiaceae*	Pogostemom alphacytorhabdovirus 2/ PogACRV2	PRJNA660501/ [37]	13209/218.9X	BK064291	N	421	StrV1-N/0.0/98/78.71
						P	359	StrV1-P/8e-164/100/66.12
						P′	64	StrV1-P′/9e-25/100/73.44
						P3	224	StrV1-P3/4e-134/100/81.7
						M	179	StrV1-M/2e-82/99/65.36
						G	549	StrV1-G/0.0/100/74.05
						P6	69	StrV1-P6/2e-31/100/72.46
						L	2083	StrV1-L/0.0/99/82.4
Patchouli (*Pogostemom cablin*)	Dicot/*Lamiaceae*	Pogostemom alphacytorhabdovirus 3_Pog/ PogACRV3_Pog	PRJNA511937/ [40]	13252/202.3X	BK064292	N	449	BmV1/0.0/99/71.05
						P	293	BmV1-P/1e-127/100/64.85
						P′	86	BmV1-P′/5e-20/100/52.33
						P3	353	BmV1-P3/0.0/96/72.14
						M	165	BmV1-M/1e-53/93/51.3
						G	544	BmV1-G/0.0/95/71.43
						P6	71	no hits
						L	2110	BmV1-L/0.0/99/72.52
Crepe myrtle (*Lagerstroemia indica*)	Dicot/*Lythraceae*	Pogostemom alphacytorhabdovirus 3_Lag/ PogACRV3_Lag	PRJNA32094/ [41]	13149/11.36X	BK064293	N	449	BmV1/0.0/99/72.20
						P	294	BmV1-P/1e-128/100/63.61
						P′	86	BmV1-P′/3e-23/100/55.81
						P3	353	BmV1-P3/0.0/96/72.14
						M	180	BmV1-M/4e-55/83/55.63
						G	544	BmV1-G/0.0/95/70.10
						P6	71	no hits
						L	2108	BmV1-L/0.0/99/72.04
Candelabra primrose (*Primula chungensis*)	Dicot/*Primulaceae*	Primula alphacytorhabdovrus1/ PriACRV1	PRJNA616180/ Wang, X., BI, Kunming, China, unpublished	12953/237.5X	BK064294	N	450	LYMV-N/0.0/99/72.1
						P	307	LYMV-P/2e-127/98/61.26
						P′	103	no hits
						P3	311	LYMV-P3/2e-161/100/70.74
						M	174	LYMV-M/2e-68/98/56.4
						G	549	LYMV-G/0.0/98/60.19
						L	2066	LYMV-L/0.0/100/73.91
Glory primrose (*Primula oreodoxa*)	Dicot/*Primulaceae*	Primula alphacytorhabdovirus 2/ PriACRV2	PRJNA544868/ [42]	12146/21.43X	BK064295	N	414	TpVA-N/1e-160/98/54.57
						P	327	GlLV1-P/2e-51/99/33.63
						P′	82	no hits
						P3	201	TpVA-P3/4e-86/98/64.5
						M	167	GlLV1-M/5e-42/91/43.79
						G	559	GlLV1-G/0.0/94/58.87
						L	2072	TpVA-L0.0/99/64.58
Beach rose (*Rosa rugosa*)	Dicot/*Rosaceae*	Rose alphacytorhabdovirus 1/ RosACRV1	PRJNA498442/ [43]	12601/230.8X	BK064296	N	425	TpVA-N/7e-152/98/51.78
						P	313	TpVA-P/3e-56/95/37.38
						P′	80	no hits
						P3	167	GlLV1-P3/4e-67/97/57.83
						M	172	TpVA-M/5e-27/100/34.48
						G	593	GlLV1-G/0.0/94/50.45
						P6	67	GlLV1-P6/1e-04/100/41.79
						L	2068	TpVA-L/0.0/99/64.85
Korean bramble (*Rubus coreanus*)	Dicot/*Rosaceae*	Rubus alphacytorhabdovirus 1/ RubACRV1	PRJNA401210/ [44]	14682/33.28X	BK064297	N	474	DV1-N/91/3e-108/40.14
						P	297	DV1-P/2e-38/98/33.22
						P′	93	DV1-P′/0.029/88/32.56
						P3	366	BmV1-P3/2e-106/93/45.45
						M	186	DV1-M/5e-10/81/28.1
						G	573	WhIV4-G/6e-154/93/41.2
						P6	64	no hits
						L	2109	WhIV4-L/0.0/99/47.44
Barbed skullcap (*Scutellaria barbata*)	*Dicot/Lamiaceae*	Scutellaria alphacytorhabdovirus 1/ ScuACRV1	PRJNA653305/ [45]	13187/56.32X	BK064298	N	447	BmV1-N/0.0/99/68.6
						P	295	BmV1-P/4e-121/100/61.36
						P′	86	BmV1-P′/3e-24/100/53.49
						P3	350	BmV1-P3/0.0/98/72.46
						M	165	BmV1-M/9e-52/93/50.65
						G	547	BmV1-G/0.0/95/70.86
						P6	75	no hits
						L	2103	BmV1-0.0/99/71.44
Piggyback plant (*Tolmiea menziesii*)	Dicot/*Saxifragaceae*	Tolmiea alphacytorhabdovirus 1/ TolACRV1	PRJNA507776/ [46]	12746/19.26X	BK064299	N	460	StrV2-N/0.0/97/67.11
						P	295	BCRV2-P/4e-130/100/62.03
						P′	102	StrV2-P′/3e-19/96/43.88
						P3	333	BCRV2-P3/7e-166/94/72.01
						M	182	BCRV2-M/3e-78/92/67.86
						G	545	BCRV2-G/0.0/96/73.14
						L	2091	BCRV2-L/0.0/99/77.05
Wheat (*Triticum aestivum*)	Monocot/*Poaceae*	Triticum alphacytorhabdovirus 1/ TriACRV1	PRJNA577739/ Li, Y., Hebei, China, unpublished	13955/87.91X	BK064300	N	474	AscSyV1-N/1e-134/93/44.02
						P	315	DV1-P/2e-47/97/36.81
						P′	87	DV1-P′/0.021/97/34.12
						P3	345	AscSyV1-P3/5e-109/90/48.96
						M	190	BmV1-M/3e-07/98/27.15
						G	561	DV1-G/6e-130/86/39.64
						P6	55	no hits
						L	2106	WhIV4-L/0.0/98/48.4
Long-leaved bladderwort (*Utricularia longifolia*)	Dicot/*Lentibulariaceae*	Utricularia alphacytorhabdovirus 1/ UtrACRV1	PRJNA354080/ Tang, C., Nanjing University, China, unpublished	13017/25.91X	BK064301	N	454	PaCRV1-N/4e-157/98/49.89
						P	324	PaCRV1-P/6e-57/100/35.17
						P3	217	PaCRV1-P3/2e73/98/52.47
						M	203	PaCRV1-M/1e-46/80/41.1
						G	571	PaCRV1-G/0.0/98/48.23
						P6	63	no hits
						L	2089	PaCRV1-L/0.0/98/59.1
Wetland metagenome	-	Wetland metagenome associated alphacytorhabdovirus 1/ WMaACRV1	PRJNA338276/ [47]	12726/43.4X	BK064302	N	445	PeVA-N/4e-174/89/57.39
						P	301	PeVA-P/1e-71/99/43.93
						P′	99	no hits
						P3	219	PeVA-P3/1e-60/98/44.39
						M	172	PeVA-M/2e-45/99/43.93
						G	551	PeVA-G/0.0/95/53.86
						P6	52	no hits
						L	2093	PeVA-L/0.0/99/62.45
Malabar cardamon (*Wurfbainia villosa*)	Monocot/*Zingiberaceae*	Wurfbainia alphacytorhabdovirus 1/ WurACRV1	PRJNA471573/ Wang, H., Guangzhou, China, unpublished	13348/59.28X	BK064303	N	465	BmV1-N/0.0/98/60.87
						P	297	BmV1-P/6e-81/99/46.49
						P′	93	BmV1-P′/1e-09/89/43.37
						P3	356	BmV1-P3/8e-143/97/56.32
						M	187	BmV1-M/1e-19/81/32.68
						G	546	BmV1-G/0.0/96/57.47
						P6	85	no hits
						L	2116	BmV1-L/0.0/99/56.5
Maize (*Zea mays*)	Monocot/*Poaceae*	Zea alphacytorhabdovirus 1/ ZeaACRV1	PRJNA543910/ Wang, J., Anhui, China, unpublished	14358/38.58X	BK064304	N	477	RVCV-N/0.0/97/53.45
						P	329	RVCV-P/3e-90/96/46.5
						P′	93	RVCV-P′/1e-05/50/48.94
						P3	242	RVCV-P36e-78/82/54.5
						M	181	RVCV-M/6e-48/96/48.28
						G	573	RVCV-G/0.0/95/55.21
						P6	64	RVCV-P6/5e-11/100/42.19
						L	2085	RVCV-L/0.0/99/65.42

**Table 2:** Summary of novel betacytorhabdoviruses identified in plant RNA-seq data available on NCBI.

Plant Host	Taxa/ Family	Virus Name/ Abbreviation	Bioproject ID/ Data Citation	Length (nt)/Coverage	Accession Number	Protein ID	Length (aa)	Highest Scoring Virus-Protein/*E*-Value/Query Coverage %/Identity % (Blast P)
Rock wormwood (*Artemisia rupestris*)	Dicot/*Asteraceae*	Artemisia betacytorhabdovirus 1/ ArtBCRV1	PRJNA730219/ [48]	13426/527.8X	BK064305	N	484	NCMV-N/2e-41/94/28.1
						P	340	no hits
						P3	201	RudV1-P3/3e-09/62/26.72
						M	202	no hits
						G	524	PpVE-G/7e-24/89/22.67
						P6	84	no hits
						L	2076	RudV1-L/0.0/99/42.91
Zip begonia (*Begonia conchifolia*)	Dicot/*Begoniaceae*	Begonia betacytorhabdovirus 1/ BegBCRV1	PRJEB26711/ [49]	13838/71.85X	BK064306	N	447	TiCRV1-N/5e-52/62/34.21
						P	299	TiCRV1-P/2e-19/97/28.04
						P3	181	no hits
						M	206	TiCRV1-M/7e-10/83/23.12
						G	572	TiCRV1-G/2e-70/85/27.93
						P6	68	no hits
						L	2161	TiCRV1-L/0.0/99/41.72
White birch (*Betula pendula*)	Dicot/*Betulaceae*	Betula betacytorhabdovirus 1/ BetBCRV1	PRJEB29260/ [50]	14744/26.93X	BK064307	N	449	RaCV-N/8e-69/90/34.24
						P	485	no hits
						P3	195	RaCV-P4/6e-19/91/23.46
						M	196	RaCV-M/1e-04/83/23.78
						G	556	RaCV-G/3e-59/92/27.08
						P6	138	no hits
						L	2242	RaCV-L/0.0/92/40.01
Himalayan birch (*Betula utilis*)	Dicot/*Betulaceae*	Betula betacytorhabdovirus 2/ BetBCRV2	PRJNA638802/ Kumar, N., CSIR, India, unpublished	15147/15.49X	BK064308	N	443	RaCV-N/2e-78/92/36.01
						P	470	no hits
						P3	196	RaCV-P4/3e-22/89/28.41
						M	193	RaCV-M/4e-08/86/25.68
						G	551	RaCV-G/2e-53/87/27.44
						P6	138	no hits
						L	2246	RaCV-L/0.0/94/39.58
Buffalo grass (*Bouteloa dactyloides*)	Monocot/*Poaceae*	Bouteloa betacytorhabdovirus 1/ BouBCRV1	PRJNA297834/ [51]	14127/373.6X	BK064309	N	452	RudV1-N/2e-65/95/32.07
						P	381	RVR-P/1e-07/17/33.33
						P3	195	RudV1-P3/9e-36/89/39.13
						M	199	RudV1-M/5e-28/84/34.32
						G	508	NCMV-G/5e-21/94/23.43
						P6	72	no hits
						P7	255	no hits
						P8	190	no hits
						L	2070	RudV1-L/0.0/99/49.04
Hardy garden mum (*Chrysanthemum morifolium*)	Dicot/*Asteraceae*	Chrysanthemum betacytorhabdovirus 1/ ChrBCRV1	PRJNA397042/ [52]	13309/99.94X	BK064310	N	450	MaCyV-N/1e-45/92/30.07
						P	333	RVR-P/0.007/26/29.55
						P3	198	TaEV1-P3/3e-11/67/29.93
						M	206	no hits
						G	511	PpVe-G/7e-22/69/24.66
						P6	86	no hits
						L	2075	RudV1-0.0/99/42.21
Siberian hazelnut (*Corylus heterophylla*)	Dicot/*Betulaceae*	Corylus betacytorhabdovirus 1/ CorBCRV1	PRJNA899668/ Sun, J, Lianoning, China, unpublished	15228/21.34X	BK064311	N	461	RaCV/7e-64/93/31.96
						P	479	no hits
						P3	197	YmVA-P4/4e-14/71/28.97
						M	201	RaCV-M/4e-04/87/21.59
						G	555	PpVE-G/3e-73/94/27.58
						P6	141	no hits
						L	2257	RaCV-L/0.0/89/40.21
Buffalo gourd (*Cucurbita foetidissima*)	Dicot/*Cucurbitaceae*	Cucurbita betacytorhabdovirus 1/ CucBCRV1	PRJNA473174/ Sun, University of California, USA, unpublished	12969/336.9X	BK064312	N	450	MaCyV-N/1e-47/94/29.88
						P	302	no hits
						P3	195	RudV1-P3/3e-08/59/31.03
						M	209	RudV1-M/2e-06/71/27.81
						G	514	RSMV-G/5e-22/93/23.35
						P6	79	no hits
						L	2079	RudV1-L/0-0/99/41.7
Slipper orchid (*Cypripedium flavum*)	Monocot/*Orchidaceae*	Cypripedium betacytorhabdovirus 1/ CypBCRV1	PRJNA479379/ [53]	9958/85.93X	BK064313	N	430	MYSV-N/1e-38/56/36
						P	280	no hits
						P3	198	no hits
						L	2114	RaCV-L/0.0/91/33.81
Keladan (*Dryobalanops oblongifolia*)	Dicot/*Dipterocarpaceae*	Dryobalanops betacytorhabdovirus 1/ DryBCRV1	PRJDB8182/ [54]	14393/134.5X	BK064314	N	495	YmVA-N/1e-95/98/35.8
						P	598	YmVA-P/6e-07/23/32
						P3	234	YmVa-P4/1e-29/68/38.04
						M	273	no hits
						G	261	no hits
						L	2259	YmVA-L/0.0/98/42.34
Durian (*Durio zibethinus*)	Dicot/*Malvaceae*	Durio betacytorhabdovirus 1/ DurBCRV1	PRJNA400310/ [55]	12791/52.47X	BK064315	N	434	NCMV-N/6e-52/97/33.03
						P	318	no hits
						P3	193	PMuMaV-P3/8e-13/68/28.79
						M	174	no hits
						G	539	RSMV-G/6e-34/84/24.74
						P6	62	no hits
						L	2057	MYSV-L/0.0/98/45.16
Littleleaf honey locust (*Gleditsia microphylla*)	Dicot/*Fabaceae*	Gleditsia betacytorhabdovirus 1/ GleBCRV1	PRJNA848854/ [56]	13339/29.81X	BK064316	N	483	YmVA-N/9e86/99/33.61
						P	454	no hits
						P3	240	YmVA-P4/6e-37/69/37.35
						M	251	no hits
						G	162	no hits
						L	2240	YmVA-L/0.0/99/40.15
Chinese licorice (*Glycyrrhiza inflata*)	Dicot/*Fabaceae*	Glycyrrhiza betacytorhabdovirus 1/ GlyBCRV1	PRJNA574093/ [57]	14755/67.34X	BK064317	N	493	YmVA-N/8e-95/96/37.89
						P	500	YmVA-P/3e-25/19/57.14
						P3	238	YmVA-P4/2e-39/76/37.57
						M	280	no hits
						G	169	no hits
						L	2264	YmVA-L/0.0/98/42.05
Pennywort (*Hepatica nobilis*)	Dicot/*Ranunculaceae*	Hepatica betacytorhabdovirus 1/ HepBCRV1	PRJDB6630/ Nodai Genome Research Center, Japan, unpublished	10440/20.81X	BK064318	N	432	RVR-N/2e-77/92/35.78
						P	384	CBDaV-P/6e-07/52/24.63
						P3	183	RVR-P3/9e-13/70/27.91
						M	162	no hits
						L	2074	RVR-L/0.0/99/48.03
Kentia palm (*Howea forsteriana*)	Monocot/*Arecaceae*	Howea betacytorhabdovirus 1/ HowBCRV1	PRJNA244607/ [58]	13727/58.86X	BK064319	N	447	TiCRV1-N/7e-46/53/36.86
						P	301	TiCRV1-P/6e-10/95/24.76
						P3	173	TiCRV1-P3/5e-11/81/25.53
						M	211	TiCRV1-M/1e-10/91/24.37
						G	557	TiCRV1-G/1e-58/92/26.15
						P6	69	no hits
						L	2145	TiCRV1-L/0.0/99/40.22
Sweet potato (*Ipomoea batatas*)	Dicot/*Convolvulaceae*	Ipomoea betacytorhabdovirus 1/ IpoBCRV1	PRJNA626066/ Read, ARC, SouthAfrica, unpublished	12811/9.76X	BK064320	N	448	NCMV-N/2e-55/93/32.62
						P	327	RVR-P/0.001/25/26.76
						P3	196	RudV1-P3/1e-06/56/30
						M	210	RudV1-M/0.015/70/24.68
						G	533	RSMV-G/1e-22/86/22.76
						P6	101	no hits
						L	2071	TaEV1-1/0.0/99/41.46
Malabar nut (*Justicia adhatoda*)	Dicot/*Acanthaceae*	Justicia betacytorhabdovirus 1/ JusBCRV1	PRJNA842169/ [59]	15957/148.3X	BK064321	N	463	RaCV-N/3e-89/99/37.26
						X	180	no hits
						P	408	no hits
						P3	198	RaCV-P4/4e-31/95/31.94
						M	199	RaCV-M/1e-08/83/23.7
						G	574	RaCV-G/8e-96/89/32.44
						P7	150	RaCV-P7/1e-06/82/29.27
						P8	140	no hits
						L	2236	RaCV/0.0/96/45.34
Royle´s sedge (*Kobresia royleana*)	Monocot/*Cyperaceae*	Kobresia betacytorhabdovirus 1/ KobBCRV1	PRJNA588660/ Qu, G., Lhasa, China, unpublished	14255/34.61X	BK064322	N	542	RSMV-N/7e-91/97/36.17
						P	550	RSMV-P/8e-07/17/34.38
						M	175	RSMV-M/0.003/88/26.28
						G	545	RSMV-G/1e-124/94/39.24
						P5	89	no hits
						L	2098	RsMV-L/0.0/99/52.39
						P7	106	no hits
Plate-seed conebush (*Leucadendron platyspermum*)	Dicot/*Proteaceae*	Leucadendron betacytorhabdovirus 1/ LeuBCRV1	PRJEB45774/ [60]	12698/181.2X	BK064323	N	445	NCMV-N/1e-115/93/43.97
						P	408	NCMV-P/2e-32/72/31.76
						P3	192	NCMV-P3/3e-09/76/26.35
						M	188	TaEV1-M/8e-08/86/26.83
						G	533	RSMV-G/9e-34/94/26.07
						P6	63	no hits
						L	2081	RudV1-L/0.0/99/42.01
Black goji (*Lycium ruthenicum*)	Dicot/*Solanaceae*	Lycium betacytorhabdovirus 1/ LycBCRV1	PRJNA505629/ [61]	14855/75.64X	BK064324	N	504	YmVA-N/2e-78/92/35.16
						P	515	YmVA-P/1e-20/22/45.76
						P3	239	YmVA-P4/2e-37/95/34.06
						M	286	YmVA-M/0.003/67/23.35
						G	208	no hits
						L	2260	YmVA-L/0.0/98/41.21
Mango (*Mangifera indica*)	Dicot/*Anacardiaceae*	Mango betacytorhabdovirus 1/ ManBCRV1	PRJNA487154/ [62]	13826/421.1X	BK064325	N	477	YmVA-N/8e-35/84/26.25
						P	359	no hits
						P3	167	no hits
						M	195	no hits
						G	577	RVR-G/7e-20/78/22.41
						P6	95	no hits
						L	2148	RaCV-L/0.0/94/35.18
White mulberry (*Morus alba*)	Dicot/*Moraceae*	Morus betacytorhabdovirus 1/ MorBCRV1	PRJNA597172/ [63]	15904/125.4X	BK064326	N	502	YmVA-N/8e-99/91/36.15
						P	617	YmVA-P/5e-32/74/29.3
						P3	231	YmVA-P4/5e-39/84/37.44
						M	263	no hits
						G	215	no hits
						L	2260	YmVA-L/0.0/98/43.14
Nitre bush (*Nitraria tangutorum*)	Dicot/*Nitrariaceae*	Nitraria betacytorhabdovirus 1/ NitBCRV1	PRJNA686177 [64]	15520/48.23X	BK064327	N	433	RaCV-N/2e-121/99/42.96
						P	544	RaCV-P/2e-14/51/27.97
						P3	189	RaCV-P4/1e-38/95/37.57
						M	187	RaCV-M/2e-21/95/30.56
						G	583	RaCV-G/6e-98/86/34.9
						P6	153	RaCV-P7/3e-09/59/32.97
						L	2246	RaCV-L/0.0/99/44.89
Hall´s panicgrass (*Panicum hallii*)	Monocot/*Poaceae*	Panicum betacytorhabdovirus 1/ PanBCRV1	PRJNA306692/ [65]	12136/60.31X	BK064328	N	418	CBDaV-N/0.0/99/72.9
						P	280	CBDaV-P/2e-128/100/66.07
						P3	195	CBDaV-P3/3e-104/94/75.14
						M	172	CBDaV-M/1e-83/98/68.05
						G	505	CBDaV-G/0.0/100/66.14
						L	2068	CBDaV-L/0.0/100/75.87
Blue passionflower (*Passiflora caerulea*)	Dicot/*Passifloraceae*	Passiflora betacytorhabdovirus 1/ PasBCRV1	PRJEB21674/ 1000 Plant (1KP) Transcriptomes Initiative, Unpublished	13471/32.88X	BK064329	N	500	TiCRV1-N/8e-130/92/44.49
						P	317	TiCRV1-P/6e-35/89/32.77
						P3	188	TiCRV1-P3/1e-22/80/29.14
						P4	69	no hits
						M	209	TiCRV1-M/5e-48/85/47.19
						G	541	TiCRV1-G/4e-160/90/44.85
						P7	72	no hits
						L	2138	TiCRV1-L/0.0/99/62.66
Peat soil	-	Peat soil associated betacytorhabdovirus 1/ PSaBCRV1	PRJNA412438/ [66]	12663/58.67X	BK064330	N	436	MYSV-N/5e-96/99/38.79
						P	315	NCMV-P/2e-26/86/31.62
						P3	186	BYSMV-P3/1e-21/78/36.99
						M	169	MYSV-M/7e-09/94/24.07
						G	505	MaCyV-G/2e-75/95/31.43
						P6	56	no hits
						L	2083	MaCyV-L/0.0/99/53.75
Peat soil	-	Peat soil associated betacytorhabdovirus 2/ PSaBCRV2	PRJNA570134/ JGI, USA, unpublished	14865/74.65X	BK064331	N	457	RudV1-N/2e-65/88/33.41
						P	397	BYSMV-P/4e-09/26/35.51
						P3	194	RudV1-P3/9e-29/78/36.84
						M	199	RudV1-M/2e-21/88/31.64
						G	516	PpVe-G/8e-35/82/25.61
						P6	66	no hits
						P7	276	no hits
						P8	182	no hits
						L	2066	RudV1-L/0.0/99/50.63
*Pentaphragma spicatum*	Dicot/*Pentaphragmataceae*	Pentaphragma betacytorhabdovirus 1/ PenBCRV1	PRJNA636634/ [67]	12983/164.1X	BK064332	N	446	TiCRV1-N/2e-50/57/34.91
						P	288	TiCRV1-P/8e-17/87/27.21
						P3	178	no hits
						M	197	TiCRV1-M/0.008/84/25.44
						G	550	TiCRV1-G/3e-76/91/30.18
						P6	63	no hits
						L	2160	TiCRV1-L/0.0/99/41.12
Amur cork tree (*Phellodendron amurense*)	Dicot/*Rutaceae*	Phellodendron betacytorhabdovirus 1/ PheBCRV1	PRJNA817294/ [68]	14292/177.4X	BK064333	N	488	YmVA-N/1e-85/97/34.43
						P	541	YmVA-P/3e-40/61/34.47
						P3	241	YmVA-P4/7e-30/78/32.28
						M	294	no hits
						G	251	no hits
						L	2258	YmVA-L/0.0/98/41.42
Desert poplar (*Populus pruinosa*)	Dicot/*Salicaceae*	Populus betacytorhabdovirus 1/ PopBCRV1	PRJNA354971/ Yu, L., Lanzhou, China, unpublished	15094/59.43X	BK064334	N	432	RaCV-N/8e-118/99/42.73
						P	569	RaCV-P/6e-10/50/22.37
						P3	188	RaCV-P4/1e-33/90/34.71
						M	201	RaCV-M/1e-17/85/26.59
						G	586	RaCV-G/2e-102/92/32.84
						P6	150	RaCV-P7/2e-10/78/31.15
						L	2246	RaCV-L/0.0/99/45.75
Kudzu (*Pueraria montana*)	Dicot/*Fabaceae*	Pueraria betacytorhabdovirus 1/ PueBCRV1	PRJNA515956/ [69]	13614/145.6X	BK064335	N	481	YmVA-N/1e-69/97/33.74
						P	338	YmVA-P/8e-11/75/41.35/
						P3	230	YmVA-P4/3e-20/58/36.57
						M	255	no hits
						G	166	no hits
						L	2254	YmVA-L/0.0/98/39.88
Sesame (*Sesamum indicum*)	Dicot/*Pedaliaceae*	Sesamum betacytorhabdovirus 1_Ses/ SesBCRV1_Ses	PRJNA644139/ [70]	13565/178.3X	BK064336	N	439	CuCV1-N/5e-72/95/34.95
						P	340	YmCaV-P/9e-15/58/30.10
						P3	183	SbBMV-P3/2e-28/73/41.18
						P4	76	no hits
						M	224	CuCV1-M/1e-23/75/30.59
						G	575	YmCaV-G/2e-100/84/35.74
						L	2113	CuCV1-L/0.0/99/48.07
Madagascar periwinkle (*Catharanthus roseus*)	Dicot/*Apocynaceae*	Sesamum betacytorhabdovirus 1_Cat/ SesBCRV1_Cat	PRJNA246273/ [71]	13497/58.97X	BK064337	N	440	CuCV1-N/1e-71/95/35.33
						P	340	YmCaV-P/9e-15/58/30.10
						P3	183	SbBMV-P3/9e-28/73/41.18
						P4	76	no hits
						M	224	CuCV1-M/7e-24/75/30.59
						G	575	YmCaV-G/3e-100/84/35.95
						L	2113	CuCV1-L/0.0/99/48.02
*Schiedea pentandra*	Dicot/*Caryophyllaceae*	Schiedea betacytorhabdovirus 1/ SchBCRV1	PRJNA491458 [72]	12964/214.6X	BK064338	N	439	MYSV-N/3e-55/93/33.01
						P	379	NCMV-P/6e-95/98/42.89
						P3	206	RVR-P3/8e-10/62/30.47
						M	182	no hits
						G	524	RSMV-G/1e-32/95/24.86
						L	2062	RudV1-L/0.0/99/43.63
						P7	114	no hits
Japanese pagoda tree (*Sophora japonica*)	Dicot/*Fabaceae*	Sophora betacytorhabdovirus 1/ SopBCRV1	PRJNA797104/ [73]	13767/149.6X	BK064339	N	501	YmVA-N/3e-94/91/36.54
						P	493	YmVA-P/2e-37/72/31.27
						P3	241	YmVA-P4/1e-39/93/35.29
						M	283	YmVA-M/0.001/61/24.57
						G	137	no hits
						L	2255	YmVA-L/0.0/97/41.79
Red clover (*Trifolium pratense*)	Dicot/*Fabaceae*	Trifolium betacytorhabdovirus 1/ TriBCRV1	PRJNA561285/ [74]	13511/285.1X	BK064340	N	429	BYSMV-N/4e-46/90/34.43
						P	372	CBDaV-P/6e-06/30/26.55
						P3	218	PMuMaV-P3/5e-19/66/31.65
						M	176	AntAmV1-M/4e-04/66/25.42
						G	529	RSMV-G/4e-37/91/25.2
						P6	72	no hits
						L	2069	BYSMV-L/0.0/99/45.15
						P8	175	no hits
Broad bean (*Vicia faba*)	Dicot/*Fabaceae*	Vicia betacytorhabdovirus 1/ VicBCRV1	PRJNA591424/ [75]	12101/269.5X	BK064341	N	434	MaCyV-N/3e-60/95/31.13
						P	441	RVR-P/0.035/16/30.99
						P3	186	RVR-P3/8e-22/75/34.04
						M	164	no hits
						L	2099	CBDaV-L/0.0/98/44.27
Japanese prickly ash (*Zanthoxilum ailanthoides*)	Dicot/*Rutaceae*	Zanthoxilum betacytorhabdovirus 1/ ZanBCRV1	PRJNA656412/ [76]	16669/78.34X	BK064342	N	488	YmVA-N/1e-88/92/33.98
						P	627	YmVA-P/2e-25/22/47.18
						P3	243	YmVA-P4/9e-26/70/33.53
						M	276	no hits
						G	278	no hits
						L	2278	YmVA-L/0.0/98/41.48
Japanese prickly ash (*Zanthoxilum ailanthoides*)	Dicot/*Rutaceae*	Zanthoxilum betacytorhabdovirus 2/ ZanBCRV2	PRJNA656412/ [76]	15584/94.97X	BK064343	N	492	YmVA-N/8e-88/91/35.01
						P	579	YmVA-P/6e-37/61/33.33
						P3	242	YmVA-P4/8e-27/68/37.35
						M	270	no hits
						G	281	no hits
						L	2280	YmVA-L/0.0/98/40.79
Japanese prickly ash (*Zanthoxilum ailanthoides*)	Dicot/*Rutaceae*	Zanthoxilum betacytorhabdovirus 3/ ZanBCRV3	PRJNA656412/ [76]	16283/50.29X	BK064344	N	492	YmVA-N/3e-88/98/34.2
						P	578	YmVA-P/6e-27/17/57.84
						P3	242	YmVA-P4/2e-24/80/32.82
						M	272	no hits
						G	283	no hits
						L	2282	YmVA-L/0.0/99/41.24

**Table 3:** Summary of novel gammacytorhabdoviruses identified from plant RNA-seq data available on NCBI.

Plant Host	Taxa/ Family	Virus Name/ Abbreviation	Bioproject ID/ Data Citation	Length (nt)/Coverage	Accession Number	Protein ID	Length (aa)	Highest Scoring Virus-Protein/*E*-Value/Query Coverage %/Identity % (Blast P)
Teide marguerite (*Argyranthemum tenerifae*)	Dicot/*Asteraceae*	Argyranthemum gammacytorhabdovirus 1/ ArgGCRV1	PRJNA491458/ [72]	10801/20.11X	BK064345	N	450	GymDenV1-N/1e-98/94/41.31
						P	297	no hits
						P3	231	TrAV1-P3/7e-28/96/29.91
						M	176	GymDenV1-M/1e-24/88/33.97
						L	2068	GymDenV1-L/0.0/99/55.15
carrot (*Daucus carota*)	Dicot/*Apiaceae*	Daucus gammacytorhabdovirus 1/ DauGCRV1	PRJNA745346/ Chakrabarti, S., CSIR-IICB, unpublished	11730/9.31X	BK064346	N	459	TrAV1-N/2e-133/95/47.42
						P	328	TrAV1-P/2e-36/99/30.65
						P3	230	TrAV1-P3/4e-52/95/39.73
						M	201	GymDenV1-M/2e-23/91/33.33
						L	2069	TrAV1-L/0.0/99/64.34
celery (*Apium graveolens*)	Dicot/*Apiaceae*	Apium gammacytorhabdovirus1/ ApiGCRV1	PRJNA543957/ [77]	12008/165.3X	BK064347	N	455	TrAV1-N/4e-173/94/57.83
						P	325	TrAV1-P/2e-81/87/47.44
						P3	233	TrAV1-P3/4e-81/94/53-95
						M	197	TrAV1-M/4e-61/94/51.87
						L	2069	TrAV1-L/0.0/100/72.5
Chinese goldthread (*Coptis chinensis*)	Dicot/*Ranunculaceae*	Coptis gammacytorhabdovirus 1/ CopGCRV1	PRJNA361017/ [78]	11214/17.41X	BK064348	N	437	GynDenV1-N/5e-118/99/42.6
						P	286	TrAV1-P/2e-30/97/28.52
						P3	227	TrAV1-P3/1e-47/93/35.81
						M	187	GymDenV1-M/2e-40/93/41.95
						L	2069	TrAV1-L/0.0/99/61.74
Bigseed alfalfa dodder (*Cuscuta indecora*)	Dicot/*Convolvulaceae*	Cuscuta gammacytorhabdovirus 1/ CusGCRV1	PRJNA543296/ [79]	10772/35.14X	BK064349	N	429	TrAV1-N/7e-108/96/43.68
						P	301	GymDenV1-P/3e-21/92/29.87
						P3	220	TrAV1-P3/3e-16/97/25.23
						M	196	GymDenV1-M/6e-13/80/32.1
						L	2054	GymDenV1-L/0.0/99/50.17
Nevada dodder (*Cuscuta nevadensis*)	Dicot/*Convolvulaceae*	Cuscuta gammacytorhabdovirus 2/ CusGCRV2	PRJNA561399/ Frangione, E., Canada, unpublished	10700/33.29X	BK064350	N	429	TrAV1-N/1e-106/96/42.49
						P	302	GymDenV1-P/9e-20/83/27.97
						P3	220	TrAV1-P3/2e-17/95/27.78
						M	188	GymDenV1-M/6e-17/85/34.15
						L	2054	GymDenV1-L/0.0/99/50.85
Slipper orchid (*Cypripedium flavum*)	Monocot/*Orchidaceae*	Cypripedium gammacytorhabdovirus 1/ CypGCRV1	PRJNA479379/ [53]	10872/30.26X	BK064351	N	437	GymDenV1-N/2e-117/96/45.5
						P	283	GymDenV1-P/2e-46/96/35.1
						P3	228	TrAV1-P3/4e-36/94/34.86
						M	213	GymDenV1-M/4e-38/83/37.64
						L	2069	GymDenV1-L/0.0/99/60.34
Violet helleborine (*Epipactis purpurata*)	Monocot/*Orchidaceae*	Epipactis gammacytorhabdovirus 1/ EpiGCRV1	PRJNA450088/ [80]	11001/38.65X	BK064352	N	452	GymDenV1-N/2e-102/86/42.36
						P	300	GymDenV1-P/4e-21/93/26.51
						P3	225	TrAV1-P3/2e-26/64/34.72
						L	2064	GymDenV1-L/0.0/99/57.83
Common ash (*Fraxinus excelsior*)	Dicot/*Oleaceae*	Fraxinus gammacytorhabdovirus 1/ FraGCRV1	PRJEB4958/ [81]	11521/50.58X	BK064353	N	443	TrAV1-1e-96/94/41.96
						P	284	GymDenV1-P/1e-26/94/28.81
						P3	224	TrAV1-P3/7e-29/97/29.41
						M	184	GymDenV1-M/2e-30/85/38.22
						P5	65	no hits
						L	2068	GymDenV1-L0.0/99/55.78
Ash dieback (*Hymenoscyphus fraxineus*)	-	Fraxinus gammacytorhabdovirus 2/ FraGCRV2	PRJEB7998/ [82]	11737/44.16X	BK064354	N	439	GymDenV1-2e-101/89/40.61
						P	285	GymDenV1-P/8e-39/94/30.51
						P3	224	TrAV1-P3/6e-33/96/34.84
						M	187	GymDenV1-M/7e-31/86/36.65
						P5	55	no hits
						L	2068	GymDenV1-L0.0/99/56
Dwarf heliosperma (*Heliosperma pusillum*)	Dicot/*Caryophyllaceae*	Heliosperma gammacytorhabdovirus 1/ HelGCRV1	PRJNA760819/ [83]	11579/19.27X	BK064355	N	436	GymDenV1-N/3e-102/90/41.65
						P	308	GymDenV1-P/2e-30/84/31.9
						P3	221	TrAV1-P3/1e-27/95/31.63
						M	206	GymDenV1-M/5e-29/83/35.67
						L	2063	GymDenV1-L/0.0/99/58.31
Kenaf (*Hibiscus cannabinus*)	Dicot/*Malvaceae*	Hibiscus gammacytorhabdovirus 1/ HibGCRV1	PRJNA602109/ [84]	11079/23.52X	BK064356	N	458	GymDenV1-N/3e-77/88/35.39
						P	391	GymDenV1-P/6e-08/62/25.99
						P3	221	TrAV1-P3/2e-16/78/26.92
						M	194	GymDenV1-M/9e-12/79/26.45
						L	2063	TrAV1-L/0.0/99/53.86
Golden ageratum (*Lonas annua*)	Dicot/*Asteraceae*	Lonas gammacytorhabdovirus 1/ LonGCRV1	PRJNA371565/ [85]	11920/115.3X	BK064357	N	450	GymDenV1-N/5e-106/88/44.75
						P	297	GymDenV1-P/1e-20/94/26.56
						P3	231	TrAV1-P3/3e-24/95/27.6
						M	176	GymDenV1-M/5e-24/88/30.77
						L	2068	GymDenV1-L/0.0/99/55.40
Mantano river lupine (*Lupinus mantaroensis*)	Dicot/*Fabaceae*	Lupinus gammacytorhabdovirus 1/ LupGCRV1	PRJNA318864/ [86]	11196/44.85X	BK064358	N	430	TrAV1-N/8e-105/98/40.95
						P	314	GymDenV1-P/9e-20/85/25.91
						P3	221	TrAV1-P3/1e-16/76/30.41
						M	189	GymDenV1-M/1e-08/84/27.16
						L	2057	TrAV1-L/0.0/99/51.47
Stinkhorn clubhead (*Rhopalocnemis phalloides*)	Dicot/*Balanophoraceae*	Rhopalocnemis gammacytorhabdovirus 1/ RhoGCRV1	PRJNA737177/ [87]	11024/32.23X	BK064359	N	469	GymDenV1-N/6e-110/86/43.06
						P	305	GymDenV1-P/1e-17/84/26.16
						P3	231	TrAV1-P3/7e-25/92/29.17
						L	2071	GymDenV1-L/0.0/99/55.86
Bladder campion (*Silene vulgaris*)	Dicot/*Caryophyllaceae*	Silene gammacytorhabdovirus 1/ SilGCRV1	PRJNA104951/ [88]	11500/37.54X	BK064360	N	435	GymDenV1-N/6e-107/89/43.83
						P	311	GymDenV1-P/3e-35/88/29.14
						P3	221	TrAV1-P3/9e-33/97/31.96
						M	209	GymDenV1-M/5e-31/80/36.09
						L	2066	GymDenV1-L/0.0/99/58.04

**Table 4:** Summary of trirhaviruses identified from plant RNA-seq data available on NCBI, including the reannotation of Picris cytorhabdovirus 1 sequence.

Plant Host	Taxa/ Family	Virus Name/ Abbreviation	Bioproject ID/ Data Citation	RNA Segment/ Length (nt)/Coverage	Accession Number	Protein ID	Length (aa)	Highest Scoring Virus-Protein/*E*-Value/Query Coverage %/Identity % (Blast P)
Red alder (*Alnus rubra*)	*Dicot/Betulaceae*	Alnus trirhavirus 1/ AlTRV1	PRJNA691057/ Bell, C., NCGR, USA, unpublished	RNA1 6699/165.78X	BK064247	L	2043	PiCRV1-L/0.0/99/55
					BK064248	N	442	PiCRV1-N/6e-62/78/34.72
				RNA2 5289/302.95X		P2	341	PiCRV1-40kDa/2e-148/99/62.28
						P3	201	PiCRV1-21kDA/5e-21/89/29.61
						P4	72	PiCRV1-8kDa/1e-15/100/52.78
						P5	312	PCLSaV-P5/4e-26/53/34.94
					BK064249	P6	260	no hits
						P7	165	no hits
				RNA3 4586/211.42X		P8	515	no hits
						P11	289	no hits
Hardy garden mum (*Chrysanthemum morifolium*)	Dicot/*Asteraceae*	Chrysanthemum trirhavirus 1/ ChTRV1	PRJNA510496/ Shen R, China, unpublished	RNA1 6332/29.57X	BK064250	L	2047	PiCRV1-L/0.0/99/58.11
					BK064251	N	441	PiCRV1-N/8e-73/77/37.29
				RNA2 4222/105.57X		P2	348	PiCRV1-40kDa/7e-154/98/63.19
						P3	189	PiCRV1-21kDA/1e-31/90/35.84
						P4	72	PiCRV1-8kDa/3e-13/100/47.22
					BK064252	P6	265	no hits
						P7	194	no hits
				RNA3 5133/66.36X		P8	528	no hits
						P5	354	PCLSaV-P5/1e-25/47/37.43
Sierra Nevada wallflower (*Erysimum nevadense*)	Dicot/*Brassicaceae*	Erysimum trirhavirus1/ EryTRV1	PRJNA473238/ [89]	RNA1 6524/16.55X	BK064253	L	2039	PiCRV1-L/0.0/99/66.22
					BK064254	N	441	PiCRV1-N/4e-111/79/45.98
				RNA2 3989/22.23X		P2	346	PiCRV1-40kDa/2e-163/99/63.48
						P3	198	PiCRV1-21kDA/1e-36/85/39.18
						P4	94	PiCRV1-8kDa/3e-22/76/62.5
					BK064255	P6	316	no hits
						P7	199	no hits
				RNA3 4307/21.74X		P8	509	no hits
						P9	143	no hits
Lucerne (*Medicago sativa*)	Dicot/*Fabaceae*	Medicago trirhavirus 1/ MeTRV1	PRJNA667169/ [90] and PRJNA535257/ JGI, USA, unpublished	RNA1 6495/9.31X	BK064256	L	2040	PiCRV1-L/0.0/99/60.28
					BK064257	N	445	PiCRV1-N/2e-113/77/48.47
				RNA2 3851/25.36X		P2	343	PiCRV1-40kDa/3e-149/97/60.90
						P3	183	PiCRV1-21kDA/2e-26/96/32.78
						P4	72	PiCRV1-8kDa/1e-18/100/62.5
					BK064258	P6	274	no hits
						P7	189	no hits
				RNA3 4565/12.23X		P8	514	no hits
						P5	303	PCLSaV-P5/1e-14/52/33.33
Bristly ox-tongue (*Picris echioides*)	Dicot/*Asteraceae*	Picris trirhavirus 1/ PiTRV1	PRJNA772045/ [20]	RNA1 6530/65.27X	BK064259	L	2043	PiCRV1-L/0.0/100/100
					BK064269	N	495	PiCRV1-N/0.0/72/100
				RNA2 4091/87.26X		P2	345	PiCRV1-40kDa/0.0/100/100
						P3	184	PiCRV1-21kDA/5e-134/100/100
						P4	72	PiCRV1-8kDa/2e-42/100/100
					BK064261	P6	331	no hits
						P7	199	no hits
				RNA3 4259/93.17X		P8	505	no hits
						P10	148	no hits

**Table 5:** Consensus conserved plant rhabdovirus gene junction sequences.

Proposed Genus	Virus *	3′ End mRNA	Intergenic Spacer	5′ End mRNA
*Alphacytorhabdovirus*	ArcACRV1	AAUUAUUUU	GAU	CUU
	ArtACRV1	AAUUCUUUU	GA(U)_n_	CNN
	ArtACRV2	AAUUAUUUU	GA(U)_n_	CNN
	ArtACRV3	AAUUAUUUU	GA(U)_n_	CNU
	BacACRV1	AAUUCUUUU	GA(U)_n_	CNC
	CarACRV1	AAUUAUUUU	GAU	CUU
	CheACRV1	AAUUAUUUU	GAU	CUU
	ChrACRV1	AAAUAUUUU	GAU	CUU
	ConACRV1	AAUUCUUUU	GAU	CNC
	CynACRV1	AAUU(C/A)UUUU	GA(U)_n_	CNN
	EupACRV1	AAUUAUUUU	GAU	CUU
	FagACRV1	AAUUAUUUU	GAU	CNN
	FicACRV1	AAUUAUUUU	GAU	CNN
	GarACRV1	AAUUCUUUU	GN(U)_n_	CNN
	GeuACRV1	AAUUCUUUU	GAU	CNC
	HedACRV1	AAUUCUUUU	GNU	CNC
	IleACRV1	AAUUAUUUU	GA(U)_n_	CUG
	MedACRV1	AAUUAUUUU	GAU	CNN
	MenACRV1	AAUUAUUUU	GAU	CUU
	MorACRV1	AAUUCUUUU	GNU	CNN
	OakACRV1	AAUUAUUUU	GAU	CUU
	OciACRV1	AAUUAUUUU	GAU	CUU
	PelACRV1	AAUUAUUUU	GAU	CUN
	PhyACRV1	AAUUCUUUU	GAU	CUC
	PinACRV1	AAUUAUUUU	GN(U)_n_	CU(U/G)
	PogACRV1	AAUUCUUUU	G(N)_n_	CUC
	PogACRV2	AAUUAUUUU	GAU	CNN
	PogACRV3	AAUUAUUUU	GAU	CNG
	PriACRV1	AAUUCUUUU	GA(U)_n_	CUN
	PriACRV2	CAUUAUUUU	GAU	CUG
	RosACRV1	AAUUAUUUU	GAU	CUN
	RubACRV1	AAUUAUUUU	GNU	CNN
	ScuACRV1	AAUUAUUUU	G(N)_n_	CNN
	TolACRV1	AAUUCUUUU	GNU	CUC
	TriACRV1	AAUUAUUUU	GA(U)_n_	CU(G/U)
	UtrACRV1	AAUUAUUUU	GA(U)_n_	CNN
	WMaACRV1	AAUUCUUUU	GAU	CUU
	WurACRV1	AAUUAUUUU	GN(U)_n_	CNN
	ZeaACRV1	AUUUAUUUU	GA(U)_n_	CNN
	AcCV	AAUUAUUUU	GAU	CUG
	ADV	AAUUAUUUU	GAU	CUU
	AscSyV1	AAUUAUUUU	GNU	CNN
	BCRV2	AAUUCUUUU	GNU	CNN
	BmV1	AAUUAUUUU	GAN	CUG
	CCyV1	AAUUCUUUU	G(N)_n_	CUU
	ChYDaV	AAUUAUUUU	GAU	CUN
	CCRV1	AAUUAUUUU	GAU	CUU
	CnV2	AAUUAUUUU	GAU	CUN
	DV1	AAUUAUUUU	GAU	CUG
	GlLV1	AAUUAUUUU	GAU	CUU
	HpLV	AAUUAUUUU	GAU	CNN
	KePCyV	AAUUAUUUU	GAU	CUU
	LNYV	AAUUCUUUU	G(N)_n_	CUU
	LYMoV	AAUUCUUUU	G(N)_n_	CUN
	NymAV1	AUUAAUUUU	GAU	CUN
	PaCRV1	AAUUAUUUU	GAU	CUU
	PCaCV	AAUUAUUUU	GNU	CUN
	PeVA	AAUUAUUUU	G(N)_n_	CUN
	PNSaV	AAUUAUUUU	GAU	CUN
	RVCV	AUUUAUUUU	GAU	CUU
	SaV1	AUUUAUUUU	GAU	CNN
	SCV	AAUUAUUUU	GAU	CUU
	StrV1	AAUUAUUUU	GAU	CUU
	StrV2	AAUUCUUUU	GNU	CNN
	TCRV1	AAUUAUUUU	GAU	CNN
	TpVA	AAUUAUUUU	GAU	CUU
	TpVB	AAUUCUUUU	G(N)_n_	CUN
	TrARV1	AAUUAUUUU	GAU	CUU
	TYMaV	AAUUAUUUU	GAU	CUU
	WhIV4	AAUUAUUUU	GNU	CUU
	WhIV5	AAUUAUUUU	GAU	CNN
	WhIV6	AAUUAUUUU	GAU	CUN
*Betacytorhabdovirus*	ArtBCRV1	AUUCUUUUU	GUU	CUU
	BegBCRV1	AUAUUUUUU	GN	CUN
	BetBCRV1	AUUCUUUUU	GG(U)_n_	CUG
	BetBCRV2	AUUCUUUUU	GG(U)_n_	CUG/A
	BouBCRV1	AUUCUUUUU	GCU	CUG
	ChrBCRV1	AUUCUUUUU	GUU	CUU
	CorBCRV1	AUUCUUUUU	GGUU	CUG
	CucBCRV1	AUUCUUUUU	G(N)_n_	CUU
	CypBCRV1	UUCUUUUUU	GA	CUC
	DryBCRV1	AUUAUUUUU	GGU	CCU
	DurBCRV1	AUUCUUUUU	GA	CUC
	GleBCRV1	AUUAUUUUU	GG(U)_n_	CUN
	GlyBCRV1	AUUAUUUUU	GGU	CCU
	HepBCRV1	AUUAUUUUU	GA(U)_n_	CUU
	HowBCRV1	AUAUUUUUU	GA	CUN
	IpoBCRV1	AUUCUUUUU	GUU	CUN
	JusBCRV1	AUU(A/C)UUUUU	GGUU	CUN
	KobBCRV1	AUUCUUUUU	GGN	CUC
	Leu CRV1	AUUCUUUUU	GA	CUC
	LycBCRV1	AUUAUUUUU	GGU	CCU
	ManBCRV1	AUUAUUUUU	GG(U)_n_	CUN
	MorBCRV1	AUUAUUUUU	GGU	CCU
	NitBCRV1	AUUCUUUUU	GGUU	CUN
	PanBCRV1	AUUCUUUUU	G(G/A)	CUC
	PasBCRV1	AUAUUUUUU	GAUU	CUC
	PSaBCRV1	AUUUAUUUU	GA	CUC
	PSaBCRV2	AUUAUUUUU	GNU	CUN
	PenBCRV1	AUAUUUUUU	G(N)_n_	CUU
	PheBCRV1	AUUAUUUUU	GGUU	CUC
	PopBCRV1	AUUCUUUUU	GG(U)_n_	CUN
	PueBCRV1	AUUAUUUUU	GGU	CCU
	SesBCRV1	UUCUUUUUU	GA	CUN
	SchBCRV1	AUUCUUUUU	GA	CUC
	SopBCRV1	AUUAUUUUU	GGU	CCU
	TriBCRV1	AUUCUUUUU	GN	CUN
	VicBCRV1	AUUCUUUUU	GG	CUC
	ZanBCRV1	AUUAUUUUU	GGU	CCU
	ZanBCRV2	AUUAUUUUU	GGU	CCU
	ZanBCRV3	AUUAUUUUU	GGU	CCU
	AntAmV1	AUUAUUUUU	GCU	CUU
	AriACRV	UUAUUUUUU	GN(N)_n_	CNN
	BeTaV1	UUAUUUUUU	GA	CUC
	BYSMV	AUUAUUUUU	GA	CUC
	CBDaV	AUUCUUUUU	GG	CUC
	CuCV1	AUUAUUUUU	GA	CUC
	MaCyV	AUUCUUUUU	GA	CUC
	MYSV	AUUAUUUUU	GA	CUC
	NCMV	AUUCUUUUU	GA	CUC
	PMuMaV	AUUAUUUUU	G(N)_n_	CUA
	PpVE	AUUCUUUUU	GAC	CCU
	RaCV	AUUCUUUUU	G(N)_n_	CUN
	RVR	AUUUAUUUU	GA	CUC
	RSMV	AUUCUUUUU	GCU	CUG
	RudV1	AUUCUUUUU	GGUU(N)_n_	CUN
	SbBMV	UUAUUUUUU	GA	CAC
	TaEV1	AUUCUUUUU	GG(N)_n_	CUN
	TiCRV1	AUAUUUUUU	GA(N)_n_	CUC
	YmCaV	UUAUUUUUU	GA	CUC
	YmVA	AUUCUUUUU	GGU	CCU
*Gammacytorhabdovirus*	ArgGCRV1	AUUCUUUUU	AAU	CCU
	CarGCRV1	AUUCUUUUU	A(N)_n_	CCU
	CelGCRV1	AUUCUUUUU	A(N)_n_	CNU
	CopGCRV1	AUUCUUUUU	A(N)_n_	CCU
	CusGCRV1	AUUCUUUUU	A(N)_n_	CNN
	CusGCRV2	AUUCUUUUU	A(N)_n_	CCU
	CypGCRV1	AAUCUUUUU	A(N)_n_	CNN
	EpiGCRV1	AUUCUUUUU	AUGU	CCU
	FraGCRV1	AUUCUUUUU	A(N)_n_	CNU
	FraGCRV2	AUUCUUUUU	A(N)_n_	CCU
	HelGCRV1	AUUCUUUUU	A(N)_n_	CCU
	HibGCRV1	AUUCUUUUU	A(N)_n_	CNN
	LonGCRV1	AUUCUUUUU	A(N)_n_	CCU
	LupGCRV1	AUUCUUUUU	A(N)_n_	CCU
	Rh GCRV1	AUUUCUUUU	A(N)_n_	CCU
	SilGCRV1	AUUCUUUUU	A(N)_n_	CCU
	GymDenV1	AAUCUUUUU	A(N)_n_	CNN
	TrAV1	AUUCUUUUU	A(N)_n_	CNU
*Trirhavirus*	AlTRV1	AAUUCUUUU	GN(N)_n_	CUC
	ChTRV1	AAUUCUUUU	GN(N)_n_	CCU
	EryTRV1	AAUUCUUUU	GN(N)_n_	CUC
	MeTRV1	AAUUCUUUU	GN(N)_n_	CU (C/G)
	PiTRV1	AAUUCUUUU	GN(N)_n_	CUN

The consensus gene junction sequences of the viruses identified in this study are highlighted in light grey. * Names and abbreviations of newly identified viruses are listed in Table 1, Table 2, Table 3 and Table 4; while the names and abbreviations of known viruses are listed in Supplementary Table S1.

## Data Availability

Nucleotide sequence data reported are available in the Third Party Annotation Section of the DDBJ/ENA/GenBank databases under the accession numbers TPA: BK064247-BK064360.

## Supplementary Materials

The following supporting information can be downloaded at: https://www.mdpi.com/article/10.3390/v15122402/s1, Figure S1: Maximum-likelihood phylogenetic tree based on amino acid sequence alignments of the complete N protein of all tri-segmented rhabdoviruses and cytorhabdoviruses reported so far and in this study constructed with the WAG + G + F model. The scale bar indicates the number of substitutions per site. Bootstrap values following 1000 replicates are given at the nodes, but only the values above 50% are shown. The viruses identified in this study are noted with green, red, violet, and blue rectangles according to proposed genus membership. Alphanucleorhabdoviruses, gymnorhaviruses and varicosaviruses were used as outgroups. Table S1: Virus names and abbreviations of cytorhabdovirus sequences used in this study. Table S2: Amino acid identity of the complete L ORF.
