# Supplementary material for: Novel Tri-Segmented Rhabdoviruses: A Data Mining Expedition Unveils the Cryptic Diversity of Cytorhabdoviruses
Source: Viruses. 2023 Dec 10;15(12):2402. doi: 10.3390/v15122402 (PMC10747219; doi:10.3390/v15122402)
Supplement: Supplementary file 1 [file viruses-15-02402-s001.zip › viruses-2733329-supplementary/figures & tables/Figure 1.pdf]

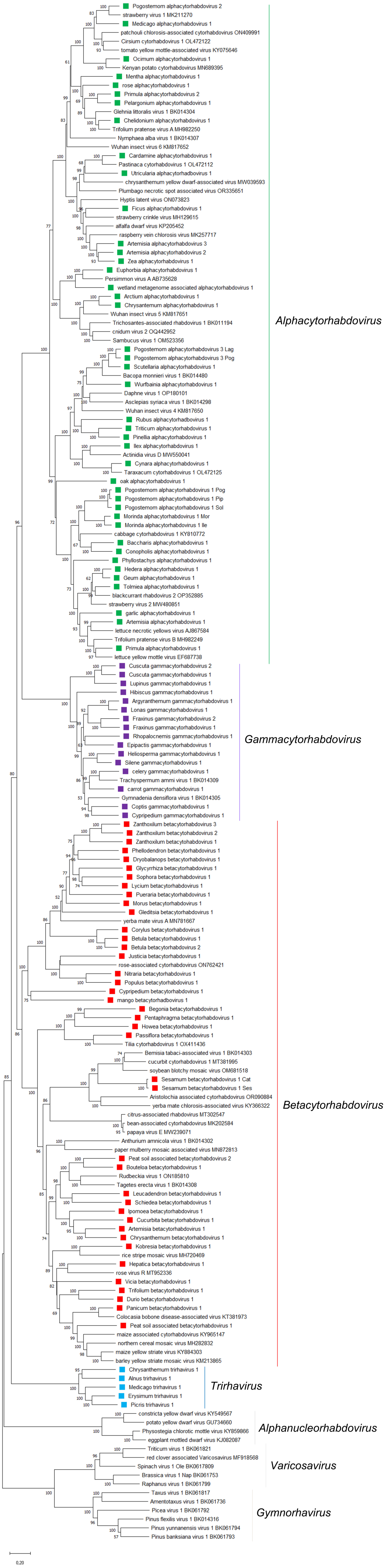
Pogostemom alphacytorhabdovirus 2
strawberry virus 1 MK211270
Medicago alphacytorhabdovirus 1
patchouli chlorosis-associated cytorhabdovirus ON409991
Cirsium cytorhabdovirus 1 OL472122
tomato yellow mottle-associated virus KY075646
Ocimum alphacytorhabdovirus 1
Kenyan potato cytorhabdovirus MN689395
Mentha alphacytorhabdovirus 1
rose alphacytorhabdovirus 1
Primula alphacytorhabdovirus 2
Pelargonium alphacytorhabdovirus 1
Glehnia littoralis virus 1 BK014304
Chelidonium alphacytorhabdovirus 1
Trifolium pratense virus A MH982250
Nymphaea alba virus 1 BK014307
Wuhan insect virus 6 KM817652
Cardamine alphacytorhabdovirus 1
Pastinaca cytorhabdovirus 1 OL472112
Utricularia alphacytorhabdovirus 1
chrysanthemum yellow dwarf-associated virus MW039593
Plumbago necrotic spot associated virus OR335651
Hyptis latent virus ON073823
Ficus alphacytorhabdovirus 1
strawberry crinkle virus MH129615
alfalfa dwarf virus KP205452
raspberry vein chlorosis virus MK257717
Artemisia alphacytorhabdovirus 3
Artemisia alphacytorhabdovirus 2
Zea alphacytorhabdovirus 1
Euphorbia alphacytorhabdovirus 1
Persimmon virus A AB735628
wetland metagenome associated alphacytorhabdovirus 1
Arctium alphacytorhabdovirus 1
Chrysantemum alphacytorhabdovirus 1
Wuhan insect virus 5 KM817651
Trichosantes-associated rhabdovirus 1 BK011194
cnidium virus 2 OQ442952
Sambucus virus 1 OM523356
Pogostemom alphacytorhabdovirus 3 Lag
Pogostemom alphacytorhabdovirus 3 Pog
Scutellaria alphacytorhabdovirus 1
Bacopa monnieri virus 1 BK014480
Wurfbainia alphacytorhabdovirus 1
Daphne virus 1 OP180101
Asclepias syriaca virus 1 BK014298
Wuhan insect virus 4 KM817650
Rubus alphacytorhabdovirus 1
Triticum alphacytorhabdovirus 1
Pinellia alphacytorhabdovirus 1
Ilex alphacytorhabdovirus 1
Actinidia virus D MW550041
Cynara alphacytorhabdovirus 1
Taraxacum cytorhabdovirus 1 OL472125
oak alphacytorhabdovirus 1
Pogostemom alphacytorhabdovirus 1 Pog
Pogostemom alphacytorhabdovirus 1 Pip
Pogostemom alphacytorhabdovirus 1 Sol
Morinda alphacytorhabdovirus 1 Mor
Morinda alphacytorhabdovirus 1 Ile
cabbage cytorhabdovirus 1 KY810772
Baccharis alphacytorhabdovirus 1
Conopholis alphacytorhabdovirus 1
Phyllostachys alphacytorhabdovirus 1
Hedera alphacytorhabdovirus 1
Geum alphacytorhabdovirus 1
Tolmiea alphacytorhabdovirus 1
blackcurrant rhabdovirus 2 OP352885
strawberry virus 2 MW480851
garlic alphacytorhabdovirus 1
Artemisia alphacytorhabdovirus 1
lettuce necrotic yellows virus AJ867584
Trifolium pratense virus B MH982249
Primula alphacytorhabdovirus 1
lettuce yellow mottle virus EF687738
Alphacytorhabdovirus
Cuscuta gammacytorhabdovirus 2
Cuscuta gammacytorhabdovirus 1
Lupinus gammacytorhabdovirus 1
Hibiscus gammacytorhabdovirus 1
Argyranthemum gammacytorhabdovirus 1
Lonas gammacytorhabdovirus 1
Fraxinus gammacytorhabdovirus 2
Fraxinus gammacytorhabdovirus 1
Rhopalocnemis gammacytorhabdovirus 1
Epipactis gammacytorhabdovirus 1
Heliosperma gammacytorhabdovirus 1
Silene gammacytorhabdovirus 1
celery gammacytorhabdovirus 1
Trachyspermum ammi virus 1 BK014309
carrot gammacytorhabdovirus 1
Gymnadenia densiflora virus 1 BK014305
Coptis gammacytorhabdovirus 1
Cypripedium gammacytorhabdovirus 1
Gammacytorhabdovirus
Zanthoxilum betacytorhabdovirus 3
Zanthoxilum betacytorhabdovirus 2
Zanthoxilum betacytorhabdovirus 1
Phellodendron betacytorhabdovirus 1
Dryobalanops betacytorhabdovirus 1
Glycyrrhiza betacytorhabdovirus 1
Sophora betacytorhabdovirus 1
Lycium betacytorhabdovirus 1
Pueraria betacytorhabdovirus 1
Morus betacytorhabdovirus 1
Gleditsia betacytorhabdovirus 1
yerba mate virus A MN781667
Corylus betacytorhabdovirus 1
Betula betacytorhabdovirus 1
Betula betacytorhabdovirus 2
Justicia betacytorhabdovirus 1
rose-associated cytorhabdovirus ON762421
Nitraria betacytorhabdovirus 1
Populus betacytorhabdovirus 1
Cypripedium betacytorhabdovirus 1
mango betacytorhadbovirus 1
Begonia betacytorhabdovirus 1
Pentaphragma betacytorhabdovirus 1
Howea betacytorhabdovirus 1
Passiflora betacytorhabdovirus 1
Tilia cytorhabdovirus 1 OX411436
Bemisia tabaci-associated virus 1 BK014303
cucurbit cytorhabdovirus 1 MT381995
soybean blotchy mosaic virus OM681518
Sesamum betacytorhabdovirus 1 Cat
Sesamum betacytorhabdovirus 1 Ses
Aristolochia associated cytorhabdovirus OR090884
yerba mate chlorosis-associated virus KY366322
citrus-associated rhabdovirus MT302547
bean-associated cytorhabdovirus MK202584
papaya virus E MW239071
Anthurium amnicola virus 1 BK014302
paper mulberry mosaic associated virus MN872813
Peat soil associated betacytorhabdovirus 2
Bouteloa betacytorhabdovirus 1
Rudbeckia virus 1 ON185810
Tagetes erecta virus 1 BK014308
Leucadendron betacytorhabdovirus 1
Schiedea betacytorhabdovirus 1
Ipomoea betacytorhabdovirus 1
Cucurbita betacytorhabdovirus 1
Artemisia betacytorhabdovirus 1
Chrysanthemum betacytorhabdovirus 1
Kobresia betacytorhabdovirus 1
rice stripe mosaic virus MH720469
Hepatica betacytorhabdovirus 1
rose virus R MT952336
Vicia betacytorhabdovirus 1
Trifolium betacytorhabdovirus 1
Durio betacytorhabdovirus 1
Panicum betacytorhabdovirus 1
Colocasia bobone disease-associated virus KT381973
Peat soil associated betacytorhabdovirus 1
maize associated cytorhabdovirus KY965147
northern cereal mosaic virus MH282832
maize yellow striate virus KY884303
barley yellow striate mosaic virus KM213865
Betacytorhabdovirus
Chrysanthemum trirhavirus 1
Alnus trirhavirus 1
Medicago trirhavirus 1
Erysimum trirhavirus 1
Picris trirhavirus 1
Trirhavirus
constricta yellow dwarf virus KY549567
potato yellow dwarf virus GU734660
Physostegia chlorotic mottle virus KY859866
eggplant mottled dwarf virus KJ082087
Alphanucleorhabdovirus
Triticum virus 1 BK061821
red clover associated Varicosavirus MF918568
Spinach virus 1 Ole BK0617809
Brassica virus 1 Nap BK061753
Raphanus virus 1 BK061799
Varicosavirus
Taxus virus 1 BK061817
Amentotaxus virus 1 BK061736
Picea virus 1 BK061792
Pinus flexilis virus 1 BK014316
Pinus yunnanensis virus 1 BK061794
Pinus banksiana virus 1 BK061793
Gymnorhavirus
0.20
