# Supplementary material for: Novel Tri-Segmented Rhabdoviruses: A Data Mining Expedition Unveils the Cryptic Diversity of Cytorhabdoviruses
Source: Viruses. 2023 Dec 10;15(12):2402. doi: 10.3390/v15122402 (PMC10747219; doi:10.3390/v15122402)
Supplement: Supplementary file 1 [file viruses-15-02402-s001.zip › viruses-2733329-supplementary/figures & tables/Figure 5.pdf]

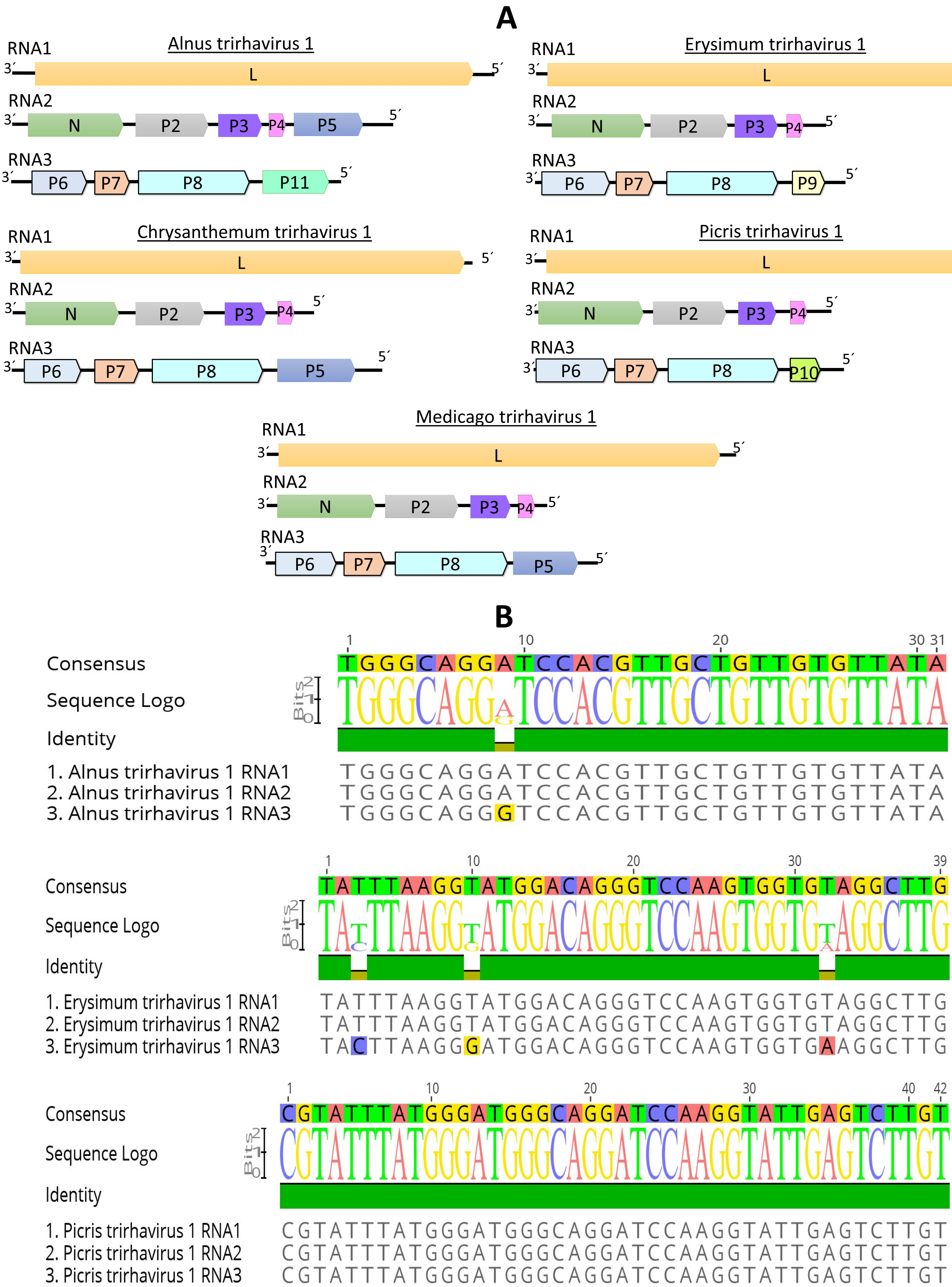
A
Alnus trirhavirus 1
RNA1
3´ L 5´
RNA2
3´ N P2 P3 P4 P5 5´
RNA3
3´ P6 P7 P8 P11 5´
Erysimum trirhavirus 1
RNA1
3´ L 5´
RNA2
3´ N P2 P3 P4 5´
RNA3
3´ P6 P7 P8 P9 5´
Chrysanthemum trirhavirus 1
RNA1
3´ L 5´
RNA2
3´ N P2 P3 P4 5´
RNA3
3´ P6 P7 P8 P5 5´
Picris trirhavirus 1
RNA1
3´ L 5´
RNA2
3´ N P2 P3 P4 5´
RNA3
3´ P6 P7 P8 P10 5´
Medicago trirhavirus 1
RNA1
3´ L 5´
RNA2
3´ N P2 P3 P4 5´
RNA3
3´ P6 P7 P8 P5 5´
B
1 10 20 30 31
Consensus TGGGCAGGATCCACGTTGCTGTTGTGTTATA
Sequence Logo
Bits 0 1 2
Identity
1. Alnus trirhavirus 1 RNA1 TGGGCAGGATCCACGTTGCTGTTGTGTTATA
2. Alnus trirhavirus 1 RNA2 TGGGCAGGATCCACGTTGCTGTTGTGTTATA
3. Alnus trirhavirus 1 RNA3 TGGGCAGGGTCCACGTTGCTGTTGTGTTATA
1 10 20 30 39
Consensus TATTTAAGGTATGGACAGGGTCCAAGTGGTGTAGGCTTG
Sequence Logo
Bits 0 1 2
Identity
1. Erysimum trirhavirus 1 RNA1 TATTTAAGGTATGGACAGGGTCCAAGTGGTGTAGGCTTG
2. Erysimum trirhavirus 1 RNA2 TATTTAAGGTATGGACAGGGTCCAAGTGGTGTAGGCTTG
3. Erysimum trirhavirus 1 RNA3 TACTTAAGGGATGGACAGGGTCCAAGTGGTGAAGGCTTG
1 10 20 30 40 42
Consensus CGTATTTATGGGATGGGCAGGATCCAAGGTATTGAGTCTTGT
Sequence Logo
Bits 0 1 2
Identity
1. Picris trirhavirus 1 RNA1 CGTATTTATGGGATGGGCAGGATCCAAGGTATTGAGTCTTGT
2. Picris trirhavirus 1 RNA2 CGTATTTATGGGATGGGCAGGATCCAAGGTATTGAGTCTTGT
3. Picris trirhavirus 1 RNA3 CGTATTTATGGGATGGGCAGGATCCAAGGTATTGAGTCTTGT
