# Supplementary material for: Novel Tri-Segmented Rhabdoviruses: A Data Mining Expedition Unveils the Cryptic Diversity of Cytorhabdoviruses
Source: Viruses. 2023 Dec 10;15(12):2402. doi: 10.3390/v15122402 (PMC10747219; doi:10.3390/v15122402)
Supplement: Supplementary file 1 [file viruses-15-02402-s001.zip › viruses-2733329-supplementary/figures & tables/Table 1.pdf]

Table 1. Summary of novel alphacytorhabdoviruses identified from plant RNA-seq data available on NCBI.

Plant host	Taxa/ family	Virus name/ Abbreviation	Bioproject ID/ Data citation	Length (nt)	Accession number	Protein ID	Length (aa)	Highest scoring virus-protein/*E*-value/query coverage%/identity% (Blast P)
Greater burdock (*Arctium lappa*)	Dicot/*Asteraceae*	Arctium alphacytorhabdovirus 1/ ArcACRV1	PRJNA598011/ [41]	12768	BK064262	N	450	WhIV5-N/0.0/91/63.07
						P	300	SaV1-P/1e-100/100/50.67
						P′	115	no hits
						P3	225	TrARV1-P3/6e-93/97/59.91
						M	182	CnV2-M/2e-54/98/47.49
						G	552	TrARV1-G/0.0/98/55.09
						L	2097	WhIV5-L/0.0/99/64.42
Silvery wormwood (*Artemisia argyi*)	Dicot/*Asteraceae*	Artemisia alphacytorhabdovirus 1/ ArtACRV1	PRJNA397671/ [42]	12978	BK064263	N	454	LNYV-N/0.0/88/60.30
						P	315	LNYV-P/9e-91/93/50.68
						P3	307	LNYV-P3/2e-119/96/54.52
						M	184	LNYV-M/2e-48/96/45.76
						G	552	LNYV-G/0.0/99/51.18
						L	2074	LNYV-L/0.0/99/68.25
Common wormwood (*Artemisia montana*)	Dicot/*Asteraceae*	Artemisia alphacytorhabdovirus 2/ ArtACRV2	PRJDB8414/ Kyoto University, Japan, unpublished	14344	BK064264	N	479	RVCV-N/1e-175/98/51.95
						P	343	RCVC-P/1e-84/92/44.55
						P′	88	RVCV-P′/2e-10/64/49.12
						P3	241	RVCV-P3/2e-74/78/56.08
						M	182	RVCV-M/1e-49/83/50.66
						G	569	RVCV-G/0.0/92/55.51
						P6	64	RVCV-P6/3e-11/90/44.83
						L	2086	RVCV-L/0.0/99/64.72
Sievers wormwood (*Artemisia sieversiana*)	Dicot/*Asteraceae*	Artemisia alphacytorhabdovirus 3/ ArtACRV3	PRJNA834888/ [43]	14339	BK064265	N	476	RVCV-N/2e-171/98/50.62
						P	342	RCVC-P/6e-82/93/43.69
						P′	103	RVCV-P′/3e-13/66/54.93
						P3	240	RVCV-P3/3e-75/82/52.40
						M	185	RVCV-M/1e-52/82/51.97
						G	571	RVCV-G/0.0/91/56.76
						P6	64	RVCV-P6/7e-14/100/48.44
						L	2089	RVCV-L/0.0/99/63.43
Desert broom (*Baccharis sarothroides*)	Dicot/*Asteraceae*	Baccharis alphacytorhabdovirus 1/	PRJNA716650/	13581	BK064266	N	470	CCyV1-N/1e-153/87/52.68
						P	297	CCyV1-P/2e-56/98/40.74

		BacACRV1	Romero, M.,<br>UNAM,<br>Mexico,<br>unpublished			P′<br>P3<br>M<br>G<br>L	85<br>337<br>168<br>550<br>2074	no hits<br>StrV2-P3/4e-120/93/56.78<br>CCyV1-M/5e-34/95/38.65<br>CCyV1-G/1e-160/92/43.14<br>CCyV1-L/0.0/99/59.24
Large bittercress<br>(*Cardamine amara*)	Dicot/*Brassicaceae*	Cardamine<br>alphacytorhabdovirus 1/<br>CarACRV1	PRJDB4989/<br>[44]	13209	BK064267	N<br>P<br>P′<br>P3<br>M<br>G<br>P6<br>L	457<br>316<br>109<br>224<br>164<br>569<br>70<br>2092	PaCRV1-N/0.0/99/72.35<br>PaCRV1-P/5e-134/63.26<br>PaCRV1-P′/1e-21/48.11<br>PaCRV1-P3/8e-122/98/75.57<br>PaCRV1-M/1e-89/100/73.78<br>PaCRV1-G/0.0/95/75.64<br>PaCRV1-P6/5e-26/97/63.24<br>PaCRV1-L/0.0/100/78.30
Greater celandine<br>(*Chelidonium majus*)	Dicot/*Papaveraceae*	Chelidonium<br>alphacytorhabdovirus 1/<br>CheACRV1	PRJNA376854/<br>Zhao, L., Jinlin,<br>China,<br>unpublished	12148	BK064268	N<br>P<br>P′<br>P3<br>M<br>G<br>P6<br>L	415<br>325<br>71<br>200<br>169<br>552<br>66<br>2072	TpVA-N/0.0/100/70.19<br>TpVA-P/2e-146/100/63.38<br>no hits<br>TpVA-P3/8e-117/98/80.71<br>TpVA-M/2e-73/92/69.43<br>TpVA-G/0.0/98/71.72<br>GlLV1-P6/2e-14/100/57.58<br>TpVA-L/0.0/100/80.41
Indian chrysanthemum<br>(*Chrysanthemum indicum*)	Dicot/*Asteraceae*	Chrysanthemum<br>alphacytorhabdovirus 1/<br>ChrACRV1	PRJNA361213/<br>[45]	12715	BK064269	N<br>P<br>P′<br>P3<br>M<br>G<br>L	448<br>301<br>140<br>225<br>19<br>549<br>2097	WhIV5-N/0.0/99/60.22<br>SaV1-P/4e-100/99/51<br>no hits<br>TrARV1-P3/1e-91/97/57.92<br>CnV2-M/6e-50/91/45.60<br>SaV1-G/0.0/100/53.42<br>WhIV5-L/0.0/99/63.89
Bear corn<br>(*Conopholis americana*)	Dicot/*Orobanchaceae*	Conopholis<br>alphacytorhabdovirus 1/<br>ConACRV1	PRJEB21674/<br>1000 Plant<br>(1KP)<br>Transcriptomes<br>Initiative,<br>Unpublished	13083	BK064270	N<br>P<br>P′<br>P3<br>M<br>G<br>L	467<br>299<br>87<br>328<br>166<br>546<br>2076	CCyV1-N/1e-157/94/49.1<br>CCyV1-P/4e-70/100/42.35<br>no hits<br>StrV2-P3/3e-119/83/60.58<br>BCRV2-M/7e-32/93/38.06<br>CCyV1-G/8e-151/99/40.26<br>CCyV1-L/0.0/98/58.75

Cardoon (*Cynara cardunculus*)	Dicot/*Asteraceae*	Cynara alphacytorhabdovirus 1/ CynACRV1	PRJNA590905/ [46]	13726	BK064271	N	472	TCRV1/0.0/100/80.08
						P	311	TCRV1-P/3e-153/100/69.97
						P´	132	TCRV1-P´/1e-23/75/53.54
						P3	350	TCRV1-P3/0.0/99/80
						M	179	TCRV1-M/3e-100/100/78.77
						G	562	TCRV1-G/0.0/98/70.40
						L	2144	TCRV1-L/0.0/98/83.25
Fischer´s spurge (*Euphorbia fischeriana*)	Dicot/*Euphorbiaceae*	Euphorbia alphacytorhabdovirus 1/ EupACRV1	PRJNA693977/ [47]	13713	BK064272	N	451	PeVA-N/0.0/95/68.41
						P	330	PeVA-P/2e-100/99/53.62
						P3	223	PeVA-P3/1e-84/100/55.61
						P4	130	no hits
						M	184	PeVA-M/5e-57/96/48.88
						G	559	PeVA-G/0.0/95/68.35
						P7	41	no hits
						L	2089	PeVA-L/0.0/99/69.3
Tikoua fig (*Ficus tikoua*)	Dicot/*Moraceae*	Ficus alphacytorhabdovirus 1/ FicACRV1	PRJNA432314/ Bai, Y., Guiyang University, China, unpublished	13839	BK064274	N	458	SCV-N/1e-140/97/48.80
						P	316	SCV-P/3e-52/88/36.51
						P´	84	no hits
						P3	228	SCV-P3/2e-75/100/50.43
						M	182	SCV-M/7e-43/82/48.67
						G	563	SCV-G/0.0/94/50.46
						P6	69	ADV-P6/3e-09/98/42.03
						L	2097	SCV-L/0.0/99/60.22
Garlic (Allium sativum)	Monocot/*Amaryllidaceae*	Garlic alphacytorhabdovirus 1/ GarACRV1	PRJNA772184/ Liu, T., IBFC, China, unpublished	13400	BK064275	N	468	LNYV-N/3e-160/93/53.17
						P	298	TpVB-P/4e-53/94/34.80
						P3	329	StrV2-P3/5e-126/98/58.28
						M	174	LNYV-M/3e-35/97/40.94
						G	554	TpVB-G/5e-175/98/44.97
						L	2075	LYMV-L/0.0/99/58.43
Herb bennet (*Geum urbanum*)	Dicot/*Rosaceae*	Geum alphacytorhabdovirus 1/ GeuACRV1	PRJEB23354/ [48]	12756	BK064276	N	459	StrV2-N/0.0/98/73.57
						P	295	StrV2-P/7e-128/100/60.34
						P´	98	StrV2-P´/3e-22/98/55.67
						P3	319	StrV2-P3/1e-171/100/74.22
						M	177	BCRV2-M/7e-78/92/68.29
						G	546	BCRV2-G/0.0/96/69.57
						L	2093	BCRV2-L/0.0/99/72.89

English ivy<br>(*Hedera helix*)	Dicot/*Araliaceae*	Hedera alphacytorhabdovirus 1/<br>HedACRV1	PRJEB21674/<br>1000 Plant (1KP) Transcriptomes Initiative, Unpublished	12588	BK064277	N<br>P<br>P´<br>P3<br>M<br>G<br>L	459<br>295<br>98<br>319<br>172<br>546<br>2100	StrV2-N/0.0/97/71.27<br>StrV2-P/3e-128/100/61.02<br>StrV2-P´/2e-22/98/53.61<br>StrV2-P3/2e-171/100/74.30<br>BCRV2-M/2e-78/99/65.50<br>BCRV2-G/0.0/99/68.19<br>BCRV2-L/0.0/98/72.95
Plum-leaved holly<br>(*Ilex asprella*)	Dicot/*Aquifoliaceae*	Ilex alphacytorhabdovirus 1/<br>IleACRV1	PRJNA736810/<br>[49]	14540	BK064278	N<br>P<br>P´<br>P3<br>M<br>G<br>P6<br>L<br>P8	452<br>333<br>93<br>370<br>184<br>553<br>54<br>2137<br>134	AcCV-N/0.0/99/56.87<br>AcCV-P/1e-67/96/39.76<br>no hits<br>AcCV-P3/1e-130/99/52.49<br>AcCV-1e-33/99/37.30<br>AcCV-G/0.0/96/49.06<br>no hits<br>AcCV-L/0.0/98/60.77<br>no hits
Lucerne<br>(*Medicago sativa*)	Dicot/*Fabaceae*	Medicago alphacytorhabdovirus 1/<br>MedACRV1	PRJNA644634/<br>[50]	13586	BK064279	N<br>P<br>P´<br>P3<br>M<br>G<br>P6<br>L	431<br>360<br>64<br>224<br>191<br>547<br>80<br>2085	StrV1-N/0.0/97/60.48<br>StrV1-1e-73/98/39.12<br>StrV1-P´/2e-13/100/54.69<br>StrV1-P3/7e-81/99/52.47<br>StrV1-M/5e-43/83/44.65<br>StrV1-G/0.0/99/60.44<br>StrV1-P6/6e-11/76/45.9<br>StrV1-L/0.0/99/68.21
Horse mint<br>(*Mentha longifolia*)	Dicot/*Lamiaceae*	Mentha alphacytorhabdovirus 1/<br>MenACRV1	PRJNA779119/<br>Wang, B., Shaoguan University, unpublished	12387	BK064280	N<br>P<br>P´<br>P3<br>M<br>G<br>P6<br>L	422<br>316<br>101<br>195<br>163<br>551<br>67<br>2072	TpVA-N/9e-163/100/54.14<br>TpVA-/7e-45/96/32.18<br>no hits<br>TpVA-P3/6e-68/97/55.38<br>GlLV1-M/6e38/95/45.16<br>TpVA-G0.0/97/55.84<br>GlLV1-P6/4e-07/100/50.75<br>TpVA-L/0.0/99/62.64
Indian mulberry<br>(*Morinda officinalis*)	Dicot/*Rubiaceae*	Morinda alphacytorhabdovirus 1_Mor/MorACRV1_Mor	PRJNA717096/<br>[51]	13023	BK064281	N<br>P<br>P´<br>P3	463<br>300<br>81<br>346	CCyV1-N/0.0/99/56.87<br>CCyV1-P/7e-68/98/42.71<br>no hits<br>BCRV2-P3/1e-115/76/59.7

						M<br>G<br>L	172<br>548<br>2075	CCyV1-M/7e-29/92/37.58<br>CCyV1-G/6e-171/93/45.14<br>CCyV1-L/0.0/99/59.16
Chinese holly<br>(*Ilex cornuta*)	Dicot/*Aquifoliaceae*	Morindaalphacytorhabdovir us 1_Ile/MorACRV1_Ile	PRJNA399054/<br>[52]	12876	BK064282	N<br>P<br>P′<br>P3<br>M<br>G<br>L	463<br>300<br>85<br>346<br>184<br>549<br>2075	CCyV1-N/0.0/99/56.29<br>CCyV1-P/1e-66/98/43.39<br>no hits<br>BCRV2-P3/2e-116/76/60.08<br>CCyV1-M/2e-27/88/36.31<br>CCyV1-G/7e-167/98/43.57<br>CCyV1-L/0.0/99/59.16
Oak<br>(*Quercus robur*)	Dicot/*Fagaceae*	Oak alphacytorhabdovirus 1/<br>OakACRV1	PRJNA322128/<br>[53]	12817	BK064283	N<br>P<br>P′<br>P3<br>M<br>G<br>L	453<br>304<br>104<br>376<br>176<br>543<br>2078	LYMV-N/2e-109/93/40.47<br>LYMV-P/1e-20/88/29.58<br>no hits<br>AscSyV1-P3/2e-46/66/35.97<br>CCyV1-M/3e-06/59/28.57<br>TrARV1-G/3e-7/98/30.16<br>BCRV2-L/0.0/99/45.57
Holy basil<br>(*Ocimum tenuiflorum*)	Dicot/*Lamiaceae*	Ocimum alphacytorhabdovirus 1/<br>OciACRV1	PRJNA251328/<br>[54]	12478	BK064284	N<br>P<br>P′<br>P3<br>M<br>G<br>P6<br>L	425<br>327<br>93<br>205<br>155<br>563<br>60<br>2078	KePCyV-N/0.0/99/76.65<br>KePCyV-P/1e-116/99/54.41<br>no hits<br>KePCyV-P3/1e-105/99/70.53<br>KePCyV-M/5e-58/98/56.21<br>KePCyV-G/0.0/96/70.46<br>StrV1-P6/2e-08/83/50<br>KePCyV-L/0.0/99/77.36
Scented pelargonium<br>(*Pelargonium* X *hybrid*)	Dicot/*Geraniaceae*	Pelargonium alphacytorhabdovirus 1/<br>PelACRV1	PRJNA883637/<br>Saint-Marcoux, D., Lyon University, France, unpublished	12332	BK064285	N<br>P<br>P′<br>P3<br>M<br>G<br>P6<br>L	413<br>328<br>84<br>203<br>170<br>552<br>58<br>2073	TpVA-N/1e-164/98/56.23<br>TpVA-P/2e-53/97/36.25<br>no hits<br>TpVA-P3/2e-91/96/68.02<br>TpVA.M/5e-37/90/44.81<br>TpVA-G/0.0/95/60.71<br>no hits<br>TpVA-L/0.0/99/63.7
Moso bamboo<br>(*Phyllostachys edulis*)	Monocot/*Poaceae*	Phyllostachys alphacytorhabdovirus 1/<br>PhyACRV1	PRJNA350353/<br>[55]	12947	BK064286	N<br>P<br>P′	455<br>296<br>83	LNYV-N/2e-138/97/46.55<br>LNYV-P/2e-44/93/37.28<br>no hits

						P3<br>M<br>G<br>L	328<br>193<br>544<br>2070	StrV2-P3/7e-140/96/59.81<br>LNYV-M/6e-28/90/34.27<br>StrV2-G/8e-144/90/40.93<br>BCRV2-L/0.0/99/57.21
Peltate green dragon (*Pinellia peltata*)	Monocot/*Araceae*	Pinellia alphacytorhabdovirus 1/ PinACRV1	PRJNA623739/ [56]	13438	BK064287	N<br>P<br>P′<br>P3<br>M<br>G<br>P6<br>L	479<br>305<br>87<br>373<br>170<br>568<br>61<br>2106	AscSyV1-N/4e-131/90/44.24<br>DV1-P/8e-47/98/36.75<br>no hits<br>AscSyV1-P3/9e-105/89/47.51<br>TCRV1-M/1e-05/88/31.21<br>WhIV4/2e-134/95/36.73<br>no hits<br>WhIV4/0.0/99/48.13
Patchouli (*Pogostemom cablin*)	Dicot/*Lamiaceae*	Pogostemom alphacytorhabdovirus 1_Pog/ PogACRV1_Pog	PRJNA660501/ [57]	13171	BK064288	N<br>P<br>P′<br>P3<br>M<br>G<br>L	462<br>300<br>81<br>347<br>179<br>557<br>2070	CCyV1-N/0.0/99/55.17<br>CCyV1-P/1e-69/96/40.79<br>no hits<br>StrV2-P3/3e-116/77/61.94<br>CCyV1-M/3e-38/92/36.14<br>CCyV1-G/1e-160/93/40.92<br>CCyV1-L/0.0/58.03
Black pepper (*Piper nigrum*)	Dicot/*Piperaceae*	Pogostemom alphacytorhabdovirus 1_ Pip/ PogACRV1_Pip	PRJNA580359/ [58]	13063	BK064289	N<br>P<br>P′<br>P3<br>M<br>G<br>L	462<br>300<br>81<br>347<br>179<br>557<br>2070	CCyV1-N/0.0/99/54.96<br>CCyV1-P/3e-67/99/40.79<br>no hits<br>StrV2-P3/3e-116/77/62.31<br>CCyV1-M/5e-39/87/39.74<br>CCyV1-G/7e-164/91/42.88<br>CCyV1-L/0.0/58.75
Tropical soda apple (*Solanum viarum*)	Dicot/*Solanaceae*	Pogostemom alphacytorhabdovirus 1_Sol/ PogACRV1_Sol	PRJNA666394/ [59]	13138	BK064290	N<br>P<br>P′<br>P3<br>M<br>G<br>L	462<br>300<br>116<br>351<br>179<br>558<br>2070	CCyV1-N/3e-179/99/54.84<br>CCyV1-P/5e-63/99/39.79<br>no hits<br>StrV2-P3/4e-115/76/61.57<br>CCyV1-M/4e-40/87/40.38<br>CCyV1-G/2e-157/95/40.98<br>CCyV1-L/0.0/58.31
Patchouli (*Pogostemom cablin*)	Dicot/*Lamiaceae*	Pogostemom alphacytorhabdovirus 2/ PogACRV2	PRJNA660501/ [57]	13209	BK064291	N<br>P<br>P′	421<br>359<br>64	StrV1-N/0.0/98/78.71<br>StrV1-P/8e-164/100/66.12<br>StrV1-P′/9e-25/100/73.44

						P3	224	StrV1-P3/4e-134/100/81.7
						M	179	StrV1-M/2e-82/99/65.36
						G	549	StrV1-G/0.0/100/74.05
						P6	69	StrV1-P6/2e-31/100/72.46
						L	2083	StrV1-L/0.0/99/82.4
Patchouli (*Pogostemom cablin*)	Dicot/*Lamiaceae*	Pogostemom alphacytorhabdovirus 3_Pog/ PogACRV3_Pog	PRJNA511937/ [60]	13252	BK064292	N	449	BmV1/0.0/99/71.05
						P	293	BmV1-P/1e-127/100/64.85
						P′	86	BmV1-P′/5e-20/100/52.33
						P3	353	BmV1-P3/0.0/96/72.14
						M	165	BmV1-M/1e-53/93/51.3
						G	544	BmV1-G/0.0/95/71.43
						P6	71	no hits
						L	2110	BmV1-L/0.0/99/72.52
Crepe myrtle (*Lagerstroemia indica*)	Dicot/*Lythraceae*	Pogostemom alphacytorhabdovirus 3_Lag/ PogACRV3_Lag	PRJNA32094/ [61]	13149	BK064293	N	449	BmV1/0.0/99/72.20
						P	294	BmV1-P/1e-128/100/63.61
						P′	86	BmV1-P′/3e-23/100/55.81
						P3	353	BmV1-P3/0.0/96/72.14
						M	180	BmV1-M/4e-55/83/55.63
						G	544	BmV1-G/0.0/95/70.10
						P6	71	no hits
						L	2108	BmV1-L/0.0/99/72.04
Candelabra primrose (*Primula chungensis*)	Dicot/*Primulaceae*	Primula alphacytorhabdovrus1/ PriACRV1	PRJNA616180/ Wang, X., BI, Kunming, China, unpublished	12953	BK064294	N	450	LYMV-N/0.0/99/72.1
						P	307	LYMV-P/2e-127/98/61.26
						P′	103	no hits
						P3	311	LYMV-P3/2e-161/100/70.74
						M	174	LYMV-M/2e-68/98/56.4
						G	549	LYMV-G/0.0/98/60.19
						L	2066	LYMV-L/0.0/100/73.91
Glory primrose (*Primula oreodoxa*)	Dicot/*Primulaceae*	Primula alphacytorhabdovirus 2/ PriACRV2	PRJNA544868/ [62]	12146	BK064295	N	414	TpVA-N/1e-160/98/54.57
						P	327	GlLV1-P/2e-51/99/33.63
						P′	82	no hits
						P3	201	TpVA-P3/4e-86/98/64.5
						M	167	GlLV1-M/5e-42/91/43.79
						G	559	GlLV1-G/0.0/94/58.87
						L	2072	TpVA-L0.0/99/64.58
Beach rose	Dicot/*Rosaceae*	Rose	PRJNA498442/	12601	BK064296	N	425	TpVA-N/7e-152/98/51.78

(*Rosa rugosa*)		alphacytorhabdovirus 1/ RosACRV1	[63]			P	313	TpVA-P/3e-56/95/37.38
						P′	80	no hits
						P3	167	GlLV1-P3/4e-67/97/57.83
						M	172	TpVA-M/5e-27/100/34.48
						G	593	GlLV1-G/0.0/94/50.45
						P6	67	GlLV1-P6/1e-04/100/41.79
						L	2068	TpVA-L/0.0/99/64.85
Korean bramble (*Rubus coreanus*)	Dicot/*Rosaceae*	Rubus alphacytorhabdovirus 1/ RubACRV1	PRJNA401210/ [64]	14682	BK064297	N	474	DV1-N/91/3e-108/40.14
						P	297	DV1-P/2e-38/98/33.22
						P′	93	DV1-P′/0.029/88/32.56
						P3	366	BmV1-P3/2e-106/93/45.45
						M	186	DV1-M/5e-10/81/28.1
						G	573	WhIV4-G/6e-154/93/41.2
						P6	64	no hits
						L	2109	WhIV4-L/0.0/99/47.44
Barbed skullcap (*Scutellaria barbata*)	*Dicot/Lamiaceae*	Scutellaria alphacytorhabdovirus 1/ ScuACRV1	PRJNA653305/ [65]	13187	BK064298	N	447	BmV1-N/0.0/99/68.6
						P	295	BmV1-P/4e-121/100/61.36
						P′	86	BmV1-P′/3e-24/100/53.49
						P3	350	BmV1-P3/0.0/98/72.46
						M	165	BmV1-M/9e-52/93/50.65
						G	547	BmV1-G/0.0/95/70.86
						P6	75	no hits
						L	2103	BmV1-0.0/99/71.44
Piggyback plant (*Tolmiea menziesii*)	Dicot/*Saxifragaceae*	Tolmiea alphacytorhabdovirus 1/ TolACRV1	PRJNA507776/ [66]	12746	BK064299	N	460	StrV2-N/0.0/97/67.11
						P	295	BCRV2-P/4e-130/100/62.03
						P′	102	StrV2-P′/3e-19/96/43.88
						P3	333	BCRV2-P3/7e-166/94/72.01
						M	182	BCRV2-M/3e-78/92/67.86
						G	545	BCRV2-G/0.0/96/73.14
						L	2091	BCRV2-L/0.0/99/77.05
Wheat (*Triticum aestivum*)	Monocot/*Poaceae*	Triticum alphacytorhabdovirus 1/ TriACRV1	PRJNA577739/ Li, Y., Hebei, China, unpublished		BK064300	N	474	AscSyV1-N/1e-134/93/44.02
						P	315	DV1-P/2e-47/97/36.81
						P′	87	DV1-P′/0.021/97/34.12
						P3	345	AscSyV1-P3/5e-109/90/48.96
						M	190	BmV1-M/3e-07/98/27.15
						G	561	DV1-G/6e-130/86/39.64

						P6<br>L	55<br>2106	no hits<br>WhIV4-L/0.0/98/48.4
Long-leaved bladderwort<br>(*Utricularia longifolia*)	Dicot/*Lentibulariaceae*	Utricularia alphacytorhabdovirus 1/<br>UtrACRV1	PRJNA354080/<br>Tang, C., Nanjing University, China, unpublished	13017	BK064301	N<br>P<br>P3<br>M<br>G<br>P6<br>L	454<br>324<br>217<br>203<br>571<br>63<br>2089	PaCRV1-N/4e-157/98/49.89<br>PaCRV1-P/6e-57/100/35.17<br>PaCRV1-P3/2e73/98/52.47<br>PaCRV1-M/1e-46/80/41.1<br>PaCRV1-G/0.0/98/48.23<br>no hits<br>PaCRV1-L/0.0/98/59.1
Wetland metagenome	-	Wetland metagenome associated alphacytorhabdovirus 1/<br>WMaACRV1	PRJNA338276/<br>[67]	12726	BK064302	N<br>P<br>P′<br>P3<br>M<br>G<br>P6<br>L	445<br>301<br>99<br>219<br>172<br>551<br>52<br>2093	PeVA-N/4e-174/89/57.39<br>PeVA-P/1e-71/99/43.93<br>no hits<br>PeVA-P3/1e-60/98/44.39<br>PeVA-M/2e-45/99/43.93<br>PeVA-G/0.0/95/53.86<br>no hits<br>PeVA-L/0.0/99/62.45
Malabar cardamon<br>(*Wurfbainia villosa*)	Monocot/*Zingiberaceae*	Wurfbainia alphacytorhabdovirus 1/<br>WurACRV1	PRJNA471573/<br>Wang, H., Guangzhou, China, unpublished	13348	BK064303	N<br>P<br>P′<br>P3<br>M<br>G<br>P6<br>L	465<br>297<br>93<br>356<br>187<br>546<br>85<br>2116	BmV1-N/0.0/98/60.87<br>BmV1-P/6e-81/99/46.49<br>BmV1-P′/1e-09/89/43.37<br>BmV1-P3/8e-143/97/56.32<br>BmV1-M/1e-19/81/32.68<br>BmV1-G/0.0/96/57.47<br>no hits<br>BmV1-L/0.0/99/56.5
Maize<br>(*Zea mays*)	Monocot/*Poaceae*	Zea alphacytorhabdovirus 1/<br>ZeaACRV1	PRJNA543910/<br>Wang, J., Anhui, China, unpublished	14358	BK064304	N<br>P<br>P′<br>P3<br>M<br>G<br>P6<br>L	477<br>329<br>93<br>242<br>181<br>573<br>64<br>2085	RVCV-N/0.0/97/53.45<br>RVCV-P/3e-90/96/46.5<br>RVCV-P′/1e-05/50/48.94<br>RVCV-P36e-78/82/54.5<br>RVCV-M/6e-48/96/48.28<br>RVCV-G/0.0/95/55.21<br>RVCV-P6/5e-11/100/42.19<br>RVCV-L/0.0/99/65.42

* Acronyms of best hits are listed in Supp. Table S1.
