# Supplementary material for: Novel Tri-Segmented Rhabdoviruses: A Data Mining Expedition Unveils the Cryptic Diversity of Cytorhabdoviruses
Source: Viruses. 2023 Dec 10;15(12):2402. doi: 10.3390/v15122402 (PMC10747219; doi:10.3390/v15122402)
Supplement: Supplementary file 1 [file viruses-15-02402-s001.zip › viruses-2733329-supplementary/figures & tables/Table 2.pdf]

Table 2. Summary of novel betacytorhabdoviruses identified in plant RNA-seq data available on NCBI.

Plant host	Taxa/ family	Virus name/ Abbreviation	Bioproject ID/ Data citation	Length (nt)	Accession number	Protein ID	Length (aa)	Highest scoring virus-protein/*E*-value/query coverage%/identity% (Blast P)
Rock wormwood (*Artemisia rupestris*)	Dicot/*Asteraceae*	Artemisia betacytorhabdovirus 1/ ArtBCRV1	PRJNA730219/ [68]	13426	BK064305	N	484	NCMV-N/2e-41/94/28.1
						P	340	no hits
						P3	201	RudV1-P3/3e-09/62/26.72
						M	202	no hits
						G	524	PpVE-G/7e-24/89/22.67
						P6	84	no hits
						L	2076	RudV1-L/0.0/99/42.91
Zip begonia (*Begonia conchifolia*)	Dicot/*Begoniaceae*	Begonia betacytorhabdovirus 1/ BegBCRV1	PRJEB26711/ [69]	13838	BK064306	N	447	TiCRV1-N/5e-52/62/34.21
						P	299	TiCRV1-P/2e-19/97/28.04
						P3	181	no hits
						M	206	TiCRV1-M/7e-10/83/23.12
						G	572	TiCRV1-G/2e-70/85/27.93
						P6	68	no hits
						L	2161	TiCRV1-L/0.0/99/41.72
White birch (*Betula pendula*)	Dicot/*Betulaceae*	Betula betacytorhabdovirus 1/ BetBCRV1	PRJEB29260/ [70]	14744	BK064307	N	449	RaCV-N/8e-69/90/34.24
						P	485	no hits
						P3	195	RaCV-P4/6e-19/91/23.46
						M	196	RaCV-M/1e-04/83/23.78
						G	556	RaCV-G/3e-59/92/27.08
						P6	138	no hits
						L	2242	RaCV-L/0.0/92/40.01
Himalayan birch (*Betula utilis*)	Dicot/*Betulaceae*	Betula betacytorhabdovirus 2/ BetBCRV2	PRJNA638802/ Kumar, N., CSIR, India, unpublished	15147	BK064308	N	443	RaCV-N/2e-78/92/36.01
						P	470	no hits
						P3	196	RaCV-P4/3e-22/89/28.41
						M	193	RaCV-M/4e-08/86/25.68
						G	551	RaCV-G/2e-53/87/27.44
						P6	138	no hits
						L	2246	RaCV-L/0.0/94/39.58
Buffalo grass (*Bouteloa dactyloides*)	Monocot/*Poaceae*	Bouteloa betacytorhabdovirus 1/ BouBCRV1	PRJNA297834/ [71]	14127	BK064309	N	452	RudV1-N/2e-65/95/32.07
						P	381	RVR-P/1e-07/17/33.33
						P3	195	RudV1-P3/9e-36/89/39.13

						M<br>G<br>P6<br>P7<br>P8<br>L	199<br>508<br>72<br>255<br>190<br>2070	RudV1-M/5e-28/84/34.32<br>NCMV-G/5e-21/94/23.43<br>no hits<br>no hits<br>no hits<br>RudV1-L/0.0/99/49.04
Hardy garden mum (*Chrysanthemum morifolium*)	Dicot/*Asteraceae*	Chrysanthemum betacytorhabdovirus 1/ ChrBCRV1	PRJNA397042/ [72]	13309	BK064310	N<br>P<br>P3<br>M<br>G<br>P6<br>L	450<br>333<br>198<br>206<br>511<br>86<br>2075	MaCyV-N/1e-45/92/30.07<br>RVR-P/0.007/26/29.55<br>TaEV1-P3/3e-11/67/29.93<br>no hits<br>PpVe-G/7e-22/69/24.66<br>no hits<br>RudV1-0.0/99/42.21
Siberian hazelnut (*Corylus heterophylla*)	Dicot/*Betulaceae*	Corylus betacytorhabdovirus 1/ CorBCRV1	PRJNA899668/ Sun, J, Lianoning, China, unpublished	15228	BK064311	N<br>P<br>P3<br>M<br>G<br>P6<br>L	461<br>479<br>197<br>201<br>555<br>141<br>2257	RaCV/7e-64/93/31.96<br>no hits<br>YmVA-P4/4e-14/71/28.97<br>RaCV-M/4e-04/87/21.59<br>PpVE-G/3e-73/94/27.58<br>no hits<br>RaCV-L/0.0/89/40.21
Buffalo gourd (*Cucurbita foetidissima*)	Dicot/*Cucurbitaceae*	Cucurbita betacytorhabdovirus 1/ CucBCRV1	PRJNA473174/ Sun, University of California, USA, unpublished	12969	BK064312	N<br>P<br>P3<br>M<br>G<br>P6<br>L	450<br>302<br>195<br>209<br>514<br>79<br>2079	MaCyV-N/1e-47/94/29.88<br>no hits<br>RudV1-P3/3e-08/59/31.03<br>RudV1-M/2e-06/71/27.81<br>RSMV-G/5e-22/93/23.35<br>no hits<br>RudV1-L/0-0/99/41.7
Slipper orchid (*Cypripedium flavum*)	Monocot/*Orchidaceae*	Cypripedium betacytorhabdovirus 1/ CypBCRV1	PRJNA479379/ [73]	9958	BK064313	N<br>P<br>P3<br>L	430<br>280<br>198<br>2114	MYSV-N/1e-38/56/36<br>no hits<br>no hits<br>RaCV-L/0.0/91/33.81
Keladan (*Dryobalanops oblongifolia*)	Dicot/*Dipterocarpaceae*	Dryobalanops betacytorhabdovirus 1/ DryBCRV1	PRJDB8182/ [74]	14393	BK064314	N<br>P<br>P3<br>M<br>G	495<br>598<br>234<br>273<br>261	YmVA-N/1e-95/98/35.8<br>YmVA-P/6e-07/23/32<br>YmVa-P4/1e-29/68/38.04<br>no hits<br>no hits

						L	2259	YmVA-L/0.0/98/42.34
Durian<br>(*Durio zibethinus*)	Dicot/*Malvaceae*	Durio<br>betacytorhabdovirus 1/<br>DurBCRV1	PRJNA400310/<br>[75]	12791	BK064315	N<br>P<br>P3<br>M<br>G<br>P6<br>L	434<br>318<br>193<br>174<br>539<br>62<br>2057	NCMV-N/6e-52/97/33.03<br>no hits<br>PMuMaV-P3/8e-13/68/28.79<br>no hits<br>RSMV-G/6e-34/84/24.74<br>no hits<br>MYSV-L/0.0/98/45.16
Littleleaf honey locust<br>(*Gleditsia microphylla*)	Dicot/*Fabaceae*	Gleditsia<br>betacytorhabdovirus 1/<br>GleBCRV1	PRJNA848854/<br>[76]	13339	BK064316	N<br>P<br>P3<br>M<br>G<br>L	483<br>454<br>240<br>251<br>162<br>2240	YmVA-N/9e86/99/33.61<br>no hits<br>YmVA-P4/6e-37/69/37.35<br>no hits<br>no hits<br>YmVA-L/0.0/99/40.15
Chinese licorice<br>(*Glycyrrhiza inflata*)	Dicot/ *Fabaceae*	Glycyrrhiza<br>betacytorhabdovirus 1/<br>GlyBCRV1	PRJNA574093/<br>[77]	14755	BK064317	N<br>P<br>P3<br>M<br>G<br>L	493<br>500<br>238<br>280<br>169<br>2264	YmVA-N/8e-95/96/37.89<br>YmVA-P/3e-25/19/57.14<br>YmVA-P4/2e-39/76/37.57<br>no hits<br>no hits<br>YmVA-L/0.0/98/42.05
Pennywort<br>(*Hepatica nobilis*)	Dicot/*Ranunculaceae*	Hepatica<br>betacytorhabdovirus 1/<br>HepBCRV1	PRJDB6630/<br>Nodai Genome<br>Research Center,<br>Japan, unpublished	10440	BK064318	N<br>P<br>P3<br>M<br>L	432<br>384<br>183<br>162<br>2074	RVR-N/2e-77/92/35.78<br>CBDaV-P/6e-07/52/24.63<br>RVR-P3/9e-13/70/27.91<br>no hits<br>RVR-L/0.0/99/48.03
Kentia palm<br>(*Howea forsteriana*)	Monocot/*Arecaceae*	Howea<br>betacytorhabdovirus 1/<br>HowBCRV1	PRJNA244607/<br>[78]	13727	BK064319	N<br>P<br>P3<br>M<br>G<br>P6<br>L	447<br>301<br>173<br>211<br>557<br>69<br>2145	TiCRV1-N/7e-46/53/36.86<br>TiCRV1-P/6e-10/95/24.76<br>TiCRV1-P3/5e-11/81/25.53<br>TiCRV1-M/1e-10/91/24.37<br>TiCRV1-G/1e-58/92/26.15<br>no hits<br>TiCRV1-L/0.0/99/40.22
Sweet potato<br>(*Ipomoea batatas*)	Dicot/*Convolvulaceae*	Ipomoea<br>betacytorhabdovirus 1/<br>IpoBCRV1	PRJNA626066/<br>Read, ARC,<br>SouthAfrica,<br>unpublished	12811	BK064320	N<br>P<br>P3<br>M	448<br>327<br>196<br>210	NCMV-N/2e-55/93/32.62<br>RVR-P/0.001/25/26.76<br>RudV1-P3/1e-06/56/30<br>RudV1-M/0.015/70/24.68

						G<br>P6<br>L	533<br>101<br>2071	RSMV-G/1e-22/86/22.76<br>no hits<br>TaEV1-1/0.0/99/41.46
Malabar nut<br>(*Justicia adhatoda*)	Dicot/*Acanthaceae*	Justicia betacytorhabdovirus 1/ JusBCRV1	PRJNA842169/ [79]	15957	BK064321	N<br>X<br>P<br>P3<br>M<br>G<br>P7<br>P8<br>L	463<br>180<br>408<br>198<br>199<br>574<br>150<br>140<br>2236	RaCV-N/3e-89/99/37.26<br>no hits<br>no hits<br>RaCV-P4/4e-31/95/31.94<br>RaCV-M/1e-08/83/23.7<br>RaCV-G/8e-96/89/32.44<br>RaCV-P7/1e-06/82/29.27<br>no hits<br>RaCV/0.0/96/45.34
Royle´s sedge<br>(*Kobresia royleana*)	Monocot/*Cyperaceae*	Kobresia betacytorhabdovirus 1/ KobBCRV1	PRJNA588660/ Qu, G., Lhasa, China, unpublished	14255	BK064322	N<br>P<br>M<br>G<br>P5<br>L<br>P7	542<br>550<br>175<br>545<br>89<br>2098<br>106	RSMV-N/7e-91/97/36.17<br>RSMV-P/8e-07/17/34.38<br>RSMV-M/0.003/88/26.28<br>RSMV-G/1e-124/94/39.24<br>no hits<br>RsMV-L/0.0/99/52.39<br>no hits
Plate-seed conebush<br>(*Leucadendron platyspermum*)	Dicot/*Proteaceae*	Leucadendron betacytorhabdovirus 1/ LeuBCRV1	PRJEB45774/ [80]	12698	BK064323	N<br>P<br>P3<br>M<br>G<br>P6<br>L	445<br>408<br>192<br>188<br>533<br>63<br>2081	NCMV-N/1e-115/93/43.97<br>NCMV-P/2e-32/72/31.76<br>NCMV-P3/3e-09/76/26.35<br>TaEV1-M/8e-08/86/26.83<br>RSMV-G/9e-34/94/26.07<br>no hits<br>RudV1-L/0.0/99/42.01
Black goji<br>(*Lycium ruthenicum*)	Dicot/*Solanaceae*	Lycium betacytorhabdovirus 1/ LycBCRV1	PRJNA505629/ [81]	14855	BK064324	N<br>P<br>P3<br>M<br>G<br>L	504<br>515<br>239<br>286<br>208<br>2260	YmVA-N/2e-78/92/35.16<br>YmVA-P/1e-20/22/45.76<br>YmVA-P4/2e-37/95/34.06<br>YmVA-M/0.003/67/23.35<br>no hits<br>YmVA-L/0.0/98/41.21
Mango<br>(*Mangifera indica*)	Dicot/*Anacardiaceae*	Mango betacytorhabdovirus 1/ ManBCRV1	PRJNA487154/ [82]	13826	BK064325	N<br>P<br>P3<br>M	477<br>359<br>167<br>195	YmVA-N/8e-35/84/26.25<br>no hits<br>no hits<br>no hits

						G<br>P6<br>L	577<br>95<br>2148	RVR-G/7e-20/78/22.41<br>no hits<br>RaCV-L/0.0/94/35.18
White mulberry<br>(*Morus alba*)	Dicot/*Moraceae*	Morus<br>betacytorhabdovirus 1/<br>MorBCRV1	PRJNA597172/<br>[83]	15904	BK064326	N<br>P<br>P3<br>M<br>G<br>L	502<br>617<br>231<br>263<br>215<br>2260	YmVA-N/8e-99/91/36.15<br>YmVA-P/5e-32/74/29.3<br>YmVA-P4/5e-39/84/37.44<br>no hits<br>no hits<br>YmVA-L/0.0/98/43.14
Nitre bush<br>(*Nitraria tangutorum*)	Dicot/*Nitrariaceae*	Nitraria<br>betacytorhabdovirus 1/<br>NitBCRV1	PRJNA686177/<br>[84]	15520	BK064327	N<br>P<br>P3<br>M<br>G<br>P6<br>L	433<br>544<br>189<br>187<br>583<br>153<br>2246	RaCV-N/2e-121/99/42.96<br>RaCV-P/2e-14/51/27.97<br>RaCV-P4/1e-38/95/37.57<br>RaCV-M/2e-21/95/30.56<br>RaCV-G/6e-98/86/34.9<br>RaCV-P7/3e-09/59/32.97<br>RaCV-L/0.0/99/44.89
Hall´s panicgrass<br>(*Panicum hallii*)	Monocot/*Poaceae*	Panicum<br>betacytorhabdovirus 1/<br>PanBCRV1	PRJNA306692/<br>[85]	12136	BK064328	N<br>P<br>P3<br>M<br>G<br>L	418<br>280<br>195<br>172<br>505<br>2068	CBDaV-N/0.0/99/72.9<br>CBDaV-P/2e-128/100/66.07<br>CBDaV-P3/3e-104/94/75.14<br>CBDaV-M/1e-83/98/68.05<br>CBDaV-G/0.0/100/66.14<br>CBDaV-L/0.0/100/75.87
Blue passionflower<br>(*Passiflora caerulea*)	Dicot/*Passifloraceae*	Passiflora<br>betacytorhabdovirus 1/<br>PasBCRV1	PRJEB21674/<br>1000 Plant (1KP)<br>Transcriptomes<br>Initiative,<br>Unpublished	13471	BK064329	N<br>P<br>P3<br>P4<br>M<br>G<br>P7<br>L	500<br>317<br>188<br>69<br>209<br>541<br>72<br>2138	TiCRV1-N/8e-130/92/44.49<br>TiCRV1-P/6e-35/89/32.77<br>TiCRV1-P3/1e-22/80/29.14<br>no hits<br>TiCRV1-M/5e-48/85/47.19<br>TiCRV1-G/4e-160/90/44.85<br>no hits<br>TiCRV1-L/0.0/99/62.66
Peat soil	-	Peat soil associated<br>betacytorhabdovirus 1/<br>PSaBCRV1	PRJNA412438/<br>[86]	12663	BK064330	N<br>P<br>P3<br>M<br>G<br>P6	436<br>315<br>186<br>169<br>505<br>56	MYSV-N/5e-96/99/38.79<br>NCMV-P/2e-26/86/31.62<br>BYSMV-P3/1e-21/78/36.99<br>MYSV-M/7e-09/94/24.07<br>MaCyV-G/2e-75/95/31.43<br>no hits

						L	2083	MaCyV-L/0.0/99/53.75
Peat soil	-	Peat soil associated betacytorhabdovirus 2/ PSaBCRV2	PRJNA570134/ JGI, USA, unpublished	14865	BK064331	N<br>P<br>P3<br>M<br>G<br>P6<br>P7<br>P8<br>L	457<br>397<br>194<br>199<br>516<br>66<br>276<br>182<br>2066	RudV1-N/2e-65/88/33.41<br>BYSMV-P/4e-09/26/35.51<br>RudV1-P3/9e-29/78/36.84<br>RudV1-M/2e-21/88/31.64<br>PpVe-G/8e-35/82/25.61<br>no hits<br>no hits<br>no hits<br>RudV1-L/0.0/99/50.63
Pentaphragma spicatum	Dicot/ *Pentaphragmataceae*	Pentaphragma betacytorhabdovirus 1/ PenBCRV1	PRJNA636634/ [87]	12983	BK064332	N<br>P<br>P3<br>M<br>G<br>P6<br>L	446<br>288<br>178<br>197<br>550<br>63<br>2160	TiCRV1-N/2e-50/57/34.91<br>TiCRV1-P/8e-17/87/27.21<br>no hits<br>TiCRV1-M/0.008/84/25.44<br>TiCRV1-G/3e-76/91/30.18<br>no hits<br>TiCRV1-L/0.0/99/41.12
Amur cork tree (*Phellodendron amurense*)	Dicot/*Rutaceae*	Phellodendron betacytorhabdovirus 1/ PheBCRV1	PRJNA817294/ [88]	14292	BK064333	N<br>P<br>P3<br>M<br>G<br>L	488<br>541<br>241<br>294<br>251<br>2258	YmVA-N/1e-85/97/34.43<br>YmVA-P/3e-40/61/34.47<br>YmVA-P4/7e-30/78/32.28<br>no hits<br>no hits<br>YmVA-L/0.0/98/41.42
Desert poplar (*Populus pruinosa*)	Dicot/*Salicaceae*	Populus betacytorhabdovirus 1/ PopBCRV1	PRJNA354971/ Yu, L., Lanzhou, China, unpublished	15094	BK064334	N<br>P<br>P3<br>M<br>G<br>P6<br>L	432<br>569<br>188<br>201<br>586<br>150<br>2246	RaCV-N/8e-118/99/42.73<br>RaCV-P/6e-10/50/22.37<br>RaCV-P4/1e-33/90/34.71<br>RaCV-M/1e-17/85/26.59<br>RaCV-G/2e-102/92/32.84<br>RaCV-P7/2e-10/78/31.15<br>RaCV-L/0.0/99/45.75
Kudzu (*Pueraria montana*)	Dicot/*Fabaceae*	Pueraria betacytorhabdovirus 1/ PueBCRV1	PRJNA515956/ [89]	13614	BK064335	N<br>P<br>P3<br>M<br>G<br>L	481<br>338<br>230<br>255<br>166<br>2254	YmVA-N/1e-69/97/33.74<br>YmVA-P/8e-11/75/41.35/<br>YmVA-P4/3e-20/58/36.57<br>no hits<br>no hits<br>YmVA-L/0.0/98/39.88

Sesame (*Sesamum indicum*)	Dicot/*Pedaliaceae*	Sesamum betacytorhabdovirus 1_Ses/ SesBCRV1_Ses	PRJNA644139/ [90]	13565	BK064336	N	439	CuCV1-N/5e-72/95/34.95
						P	340	YmCaV-P/9e-15/58/30.10
						P3	183	SbBMV-P3/2e-28/73/41.18
						P4	76	no hits
						M	224	CuCV1-M/1e-23/75/30.59
						G	575	YmCaV-G/2e-100/84/35.74
						L	2113	CuCV1-L/0.0/99/48.07
Madagascar periwinkle (*Catharanthus roseus*)	Dicot/*Apocynaceae*	Sesamum betacytorhabdovirus 1_Cat/ SesBCRV1_Cat	PRJNA246273/ [91]	13497	BK064337	N	440	CuCV1-N/1e-71/95/35.33
						P	340	YmCaV-P/9e-15/58/30.10
						P3	183	SbBMV-P3/9e-28/73/41.18
						P4	76	no hits
						M	224	CuCV1-M/7e-24/75/30.59
						G	575	YmCaV-G/3e-100/84/35.95
						L	2113	CuCV1-L/0.0/99/48.02
Schiedea pentandra	Dicot/*Caryophyllaceae*	Schiedea betacytorhabdovirus 1/ SchBCRV1	PRJNA491458 [92]	12964	BK064338	N	439	MYSV-N/3e-55/93/33.01
						P	379	NCMV-P/6e-95/98/42.89
						P3	206	RVR-P3/8e-10/62/30.47
						M	182	no hits
						G	524	RSMV-G/1e-32/95/24.86
						L	2062	RudV1-L/0.0/99/43.63
						P7	114	no hits
Japanese pagoda tree (*Sophora japonica*)	Dicot/*Fabaceae*	Sophora betacytorhabdovirus 1/ SopBCRV1	PRJNA797104/ [93]	13767	BK064339	N	501	YmVA-N/3e-94/91/36.54
						P	493	YmVA-P/2e-37/72/31.27
						P3	241	YmVA-P4/1e-39/93/35.29
						M	283	YmVA-M/0.001/61/24.57
						G	137	no hits
						L	2255	YmVA-L/0.0/97/41.79
Red clover (*Trifolium pratense*)	Dicot/*Fabaceae*	Trifolium betacytorhabdovirus 1/ TriBCRV1	PRJNA561285/ [94]	13511	BK064340	N	429	BYSMV-N/4e-46/90/34.43
						P	372	CBDaV-P/6e-06/30/26.55
						P3	218	PMuMaV-P3/5e-19/66/31.65
						M	176	AntAmV1-M/4e-04/66/25.42
						G	529	RSMV-G/4e-37/91/25.2
						P6	72	no hits
						L	2069	BYSMV-L/0.0/99/45.15
						P8	175	no hits

Broad bean (*Vicia faba*)	Dicot/ *Fabaceae*	Vicia betacytorhabdovirus 1/ VicBCRV1	PRJNA591424/ [95]	12101	BK064341	N	434	MaCyV-N/3e-60/95/31.13
						P	441	RVR-P/0.035/16/30.99
						P3	186	RVR-P3/8e-22/75/34.04
						M	164	no hits
						L	2099	CBDaV-L/0.0/98/44.27
Japanese prickly ash (*Zanthoxilum ailanthoides*)	Dicot/*Rutaceae*	Zanthoxilum betacytorhabdovirus 1/ ZanBCRV1	PRJNA656412/ [96]	16669	BK064342	N	488	YmVA-N/1e-88/92/33.98
						P	627	YmVA-P/2e-25/22/47.18
						P3	243	YmVA-P4/9e-26/70/33.53
						M	276	no hits
						G	278	no hits
						L	2278	YmVA-L/0.0/98/41.48
Japanese prickly ash (*Zanthoxilum ailanthoides*)	Dicot/*Rutaceae*	Zanthoxilum betacytorhabdovirus 2/ ZanBCRV2	PRJNA656412/ [96]	15584	BK064343	N	492	YmVA-N/8e-88/91/35.01
						P	579	YmVA-P/6e-37/61/33.33
						P3	242	YmVA-P4/8e-27/68/37.35
						M	270	no hits
						G	281	no hits
						L	2280	YmVA-L/0.0/98/40.79
Japanese prickly ash (*Zanthoxilum ailanthoides*)	Dicot/*Rutaceae*	Zanthoxilum betacytorhabdovirus 3/ ZanBCRV3	PRJNA656412/ [96]	16283	BK064344	N	492	YmVA-N/3e-88/98/34.2
						P	578	YmVA-P/6e-27/17/57.84
						P3	242	YmVA-P4/2e-24/80/32.82
						M	272	no hits
						G	283	no hits
						L	2282	YmVA-L/0.0/99/41.24

* Acronyms of best hits are listed in Supp. Table S1.
