# Supplementary material for: Novel Tri-Segmented Rhabdoviruses: A Data Mining Expedition Unveils the Cryptic Diversity of Cytorhabdoviruses
Source: Viruses. 2023 Dec 10;15(12):2402. doi: 10.3390/v15122402 (PMC10747219; doi:10.3390/v15122402)
Supplement: Supplementary file 1 [file viruses-15-02402-s001.zip › viruses-2733329-supplementary/figures & tables/Table 3.pdf]

Table 3. Summary of novel gammacytorhabdoviruses identified from plant RNA-seq data available on NCBI.

Plant host	Taxa/ family	Virus name/ Abbreviation	Bioproject ID/ Data citation	Length (nt)	Accession number	Protein ID	Length (aa)	Highest scoring virus-protein/*E*-value/query coverage%/identity% (Blast P)
Teide marguerite (*Argyranthemum tenerifae*)	Dicot/*Asteraceae*	Argyranthemum gammacytorhabdovirus 1/ ArgGCRV1	PRJNA491458/ [92]	10801	BK064345	N<br>P<br>P3<br>M<br>L	450<br>297<br>231<br>176<br>2068	GymDenV1-N/1e-98/94/41.31<br>no hits<br>TrAV1-P3/7e-28/96/29.91<br>GymDenV1-M/1e-24/88/33.97<br>GymDenV1-L/0.0/99/55.15
carrot (*Daucus carota*)	Dicot/*Apiaceae*	Daucus gammacytorhabdovirus 1/ DauGCRV1	PRJNA745346/ Chakrabarti, S., CSIR-IICB, unpublished	11730	BK064346	N<br>P<br>P3<br>M<br>L	459<br>328<br>230<br>201<br>2069	TrAV1-N/2e-133/95/47.42<br>TrAV1-P/2e-36/99/30.65<br>TrAV1-P3/4e-52/95/39.73<br>GymDenV1-M/2e-23/91/33.33<br>TrAV1-L/0.0/99/64.34
celery (*Apium graveolens*)	Dicot/*Apiaceae*	Apium gammacytorhabdovirus1/ ApiGCRV1	PRJNA543957/ [97]	12008	BK064347	N<br>P<br>P3<br>M<br>L	455<br>325<br>233<br>197<br>2069	TrAV1-N/4e-173/94/57.83<br>TrAV1-P/2e-81/87/47.44<br>TrAV1-P3/4e-81/94/53-95<br>TrAV1-M/4e-61/94/51.87<br>TrAV1-L/0.0/100/72.5
Chinese goldthread (*Coptis chinensis*)	Dicot/*Ranunculaceae*	Coptis gammacytorhabdovirus 1/ CopGCRV1	PRJNA361017/ [98]	11214	BK064348	N<br>P<br>P3<br>M<br>L	437<br>286<br>227<br>187<br>2069	GynDenV1-N/5e-118/99/42.6<br>TrAV1-P/2e-30/97/28.52<br>TrAV1-P3/1e-47/93/35.81<br>GymDenV1-M/2e-40/93/41.95<br>TrAV1-L/0.0/99/61.74
Bigseed alfalfa dodder (*Cuscuta indecora*)	Dicot/*Convolvulaceae*	Cuscuta gammacytorhabdovirus 1/ CusGCRV1	PRJNA543296/ [99]	10772	BK064349	N<br>P<br>P3<br>M<br>L	429<br>301<br>220<br>196<br>2054	TrAV1-N/7e-108/96/43.68<br>GymDenV1-P/3e-21/92/29.87<br>TrAV1-P3/3e-16/97/25.23<br>GymDenV1-M/6e-13/80/32.1<br>GymDenV1-L/0.0/99/50.17
Nevada dodder (*Cuscuta nevadensis*)	Dicot/*Convolvulaceae*	Cuscuta gammacytorhabdovirus 2/ CusGCRV2	PRJNA561399/ Frangione, E., Canada, unpublished	10700	BK064350	N<br>P<br>P3<br>M<br>L	429<br>302<br>220<br>188<br>2054	TrAV1-N/1e-106/96/42.49<br>GymDenV1-P/9e-20/83/27.97<br>TrAV1-P3/2e-17/95/27.78<br>GymDenV1-M/6e-17/85/34.15<br>GymDenV1-L/0.0/99/50.85

Slipper orchid (*Cypripedium flavum*)	Monocot/*Orchidaceae*	Cypripedium gammacytorhabdovirus 1/ CypGCRV1	PRJNA479379/ [73]	10872	BK064351	N<br>P<br>P3<br>M<br>L	437<br>283<br>228<br>213<br>2069	GymDenV1-N/2e-117/96/45.5<br>GymDenV1-P/2e-46/96/35.1<br>TrAV1-P3/4e-36/94/34.86<br>GymDenV1-M/4e-38/83/37.64<br>GymDenV1-L/0.0/99/60.34
Violet helleborine (*Epipactis purpurata*)	Monocot/*Orchidaceae*	Epipactis gammacytorhabdovirus 1/ EpiGCRV1	PRJNA450088/ [100]	11001	BK064352	N<br>P<br>P3<br>L	452<br>300<br>225<br>2064	GymDenV1-N/2e-102/86/42.36<br>GymDenV1-P/4e-21/93/26.51<br>TrAV1-P3/2e-26/64/34.72<br>GymDenV1-L/0.0/99/57.83
Common ash (*Fraxinus excelsior*)	Dicot/*Oleaceae*	Fraxinus gammacytorhabdovirus 1/ FraGCRV1	PRJEB4958/ [101]	11521	BK064353	N<br>P<br>P3<br>M<br>P5<br>L	443<br>284<br>224<br>184<br>65<br>2068	TrAV1-1e-96/94/41.96<br>GymDenV1-P/1e-26/94/28.81<br>TrAV1-P3/7e-29/97/29.41<br>GymDenV1-M/2e-30/85/38.22<br>no hits<br>GymDenV1-L0.0/99/55.78
Ash dieback (*Hymenoscyphus fraxineus*)	-	Fraxinus gammacytorhabdovirus 2/ FraGCRV2	PRJEB7998/ [102]	11737	BK064354	N<br>P<br>P3<br>M<br>P5<br>L	439<br>285<br>224<br>187<br>55<br>2068	GymDenV1-2e-101/89/40.61<br>GymDenV1-P/8e-39/94/30.51<br>TrAV1-P3/6e-33/96/34.84<br>GymDenV1-M/7e-31/86/36.65<br>no hits<br>GymDenV1-L0.0/99/56
Dwarf heliosperma (*Heliosperma pusillum*)	Dicot/*Caryophyllaceae*	Heliosperma gammacytorhabdovirus 1/ HelGCRV1	PRJNA760819/ [103]	11579	BK064355	N<br>P<br>P3<br>M<br>L	436<br>308<br>221<br>206<br>2063	GymDenV1-N/3e-102/90/41.65<br>GymDenV1-P/2e-30/84/31.9<br>TrAV1-P3/1e-27/95/31.63<br>GymDenV1-M/5e-29/83/35.67<br>GymDenV1-L/0.0/99/58.31
Kenaf (*Hibiscus cannabinus*)	Dicot/*Malvaceae*	Hibiscus gammacytorhabdovirus 1/ HibGCRV1	PRJNA602109/ [104]	11079	BK064356	N<br>P<br>P3<br>M<br>L	458<br>391<br>221<br>194<br>2063	GymDenV1-N/3e-77/88/35.39<br>GymDenV1-P/6e-08/62/25.99<br>TrAV1-P3/2e-16/78/26.92<br>GymDenV1-M/9e-12/79/26.45<br>TrAV1-L/0.0/99/53.86
Golden ageratum (*Lonas annua*)	Dicot/*Asteraceae*	Lonas gammacytorhabdovirus 1/ LonGCRV1	PRJNA371565/ [105]	11920	BK064357	N<br>P<br>P3<br>M<br>L	450<br>297<br>231<br>176<br>2068	GymDenV1-N/5e-106/88/44.75<br>GymDenV1-P/1e-20/94/26.56<br>TrAV1-P3/3e-24/95/27.6<br>GymDenV1-M/5e-24/88/30.77<br>GymDenV1-L/0.0/99/55.40

Mantano river lupine (*Lupinus mantaroensis*)	Dicot/*Fabaceae*	Lupinus gammacytorhabdovirus 1/ LupGCRV1	PRJNA318864/ [106]	11196	BK064358	N<br>P<br>P3<br>M<br>L	430<br>314<br>221<br>189<br>2057	TrAV1-N/8e-105/98/40.95<br>GymDenV1-P/9e-20/85/25.91<br>TrAV1-P3/1e-16/76/30.41<br>GymDenV1-M/1e-08/84/27.16<br>TrAV1-L/0.0/99/51.47
Stinkhorn clubhead (*Rhopalocnemis phalloides*)	Dicot/*Balanophoraceae*	Rhopalocnemis gammacytorhabdovirus 1/ RhoGCRV1	PRJNA737177/ [107]	11024	BK064359	N<br>P<br>P3<br>L	469<br>305<br>231<br>2071	GymDenV1-N/6e-110/86/43.06<br>GymDenV1-P/1e-17/84/26.16<br>TrAV1-P3/7e-25/92/29.17<br>GymDenV1-L/0.0/99/55.86
Bladder campion (*Silene vulgaris*)	Dicot/*Caryophyllaceae*	Silene gammacytorhabdovirus 1/ SilGCRV1	PRJNA104951/ [108]	11500	BK064360	N<br>P<br>P3<br>M<br>L	435<br>311<br>221<br>209<br>2066	GymDenV1-N/6e-107/89/43.83<br>GymDenV1-P/3e-35/88/29.14<br>TrAV1-P3/9e-33/97/31.96<br>GymDenV1-M/5e-31/80/36.09<br>GymDenV1-L/0.0/99/58.04

* Acronyms of best hits are listed in Supp. Table S1.
