# Supplementary material for: Novel Tri-Segmented Rhabdoviruses: A Data Mining Expedition Unveils the Cryptic Diversity of Cytorhabdoviruses
Source: Viruses. 2023 Dec 10;15(12):2402. doi: 10.3390/v15122402 (PMC10747219; doi:10.3390/v15122402)
Supplement: Supplementary file 1 [file viruses-15-02402-s001.zip › viruses-2733329-supplementary/figures & tables/Table 4.pdf]

Table 4. Summary of trirhaviruses identified from plant RNA-seq data available on NCBI, including the reannotation of Picris cytorhabdovirus 1 sequence.

Plant host	Taxa/ family	Virus name/ Abbreviation	Bioproject ID/ Data citation	RNA segment/ Length (nt)	Accession number	Protein ID	Length (aa)	Highest scoring virus-protein/*E*-value/query coverage%/identity% (Blast P)
Red alder (*Alnus rubra*)	*Dicot/Betulaceae*	Alnus trirhavirus 1/ AlTRV1	PRJNA691057/ Bell, C., NCGR, USA,unpublished	RNA1 6699	BK064247	L	2043	PiCRV1-L/0.0/99/55
				RNA2 5289	BK064248	N	442	PiCRV1-N/6e-62/78/34.72
						P2	341	PiCRV1-40kDa/2e-148/99/62.28
						P3	201	PiCRV1-21kDA/5e-21/89/29.61
						P4	72	PiCRV1-8kDa/1e-15/100/52.78
						P5	312	PCLSaV-P5/4e-26/53/34.94
				RNA3 4586	BK064249	P6	260	no hits
						P7	165	no hits
						P8	515	no hits
						P11	289	no hits
Hardy garden mum (*Chrysanthemum morifolium*)	Dicot/*Asteraceae*	Chrysanthemum trirhavirus 1/ ChTRV1	PRJNA510496/ Shen R, China, unpublished	RNA1 6332	BK064250	L	2047	PiCRV1-L/0.0/99/58.11
				RNA2 4222	BK064251	N	441	PiCRV1-N/8e-73/77/37.29
						P2	348	PiCRV1-40kDa/7e-154/98/63.19
						P3	189	PiCRV1-21kDA/1e-31/90/35.84
						P4	72	PiCRV1-8kDa/3e-13/100/47.22
				RNA3 5133	BK064252	P6	265	no hits
						P7	194	no hits
						P8	528	no hits
						P5	354	PCLSaV-P5/1e-25/47/37.43
Sierra Nevada wallflower (*Erysimum nevadense*)	Dicot/*Brassicaceae*	Erysimum trirhavirus1/ EryTRV1	PRJNA473238/ [109]	RNA1 6524	BK064253	L	2039	PiCRV1-L/0.0/99/66.22
				RNA2 3989	BK064254	N	441	PiCRV1-N/4e-111/79/45.98
						P2	346	PiCRV1-40kDa/2e-163/99/63.48
						P3	198	PiCRV1-21kDA/1e-36/85/39.18
						P4	94	PiCRV1-8kDa/3e-22/76/62.5
				RNA3 4307	BK064255	P6	316	no hits
						P7	199	no hits
						P8	509	no hits
						P9	143	no hits
Lucerne (*Medicago sativa*)	Dicot/*Fabaceae*	Medicago trirhavirus 1/ MeTRV1	PRJNA667169/ [110] and	RNA1 6495	BK064256	L	2040	PiCRV1-L/0.0/99/60.28
				RNA2 3851	BK064257	N	445	PiCRV1-N/2e-113/77/48.47
						P2	343	PiCRV1-40kDa/3e-149/97/60.90

			PRJNA535257/			P3	183	PiCRV1-21kDA/2e-26/96/32.78
			JGI, USA,			P4	72	PiCRV1-8kDa/1e-18/100/62.5
			unpublished	RNA3 4565	BK064258	P6	274	no hits
						P7	189	no hits
						P8	514	no hits
						P5	303	PCLSaV-P5/1e-14/52/33.33
Bristly ox-tongue	Dicot/*Asteraceae*	Picris trirhavirus	PRJNA772045/	RNA1 6530	BK064259	L	2043	PiCRV1-L/0.0/100/100
(*Picris echioides*)		1/	[20]	RNA2 4091	BK064269	N	495	PiCRV1-N/0.0/72/100
		PiTRV1				P2	345	PiCRV1-40kDa/0.0/100/100
						P3	184	PiCRV1-21kDA/5e-134/100/100
						P4	72	PiCRV1-8kDa/2e-42/100/100
				RNA3 4259	BK064261	P6	331	no hits
						P7	199	no hits
						P8	505	no hits
						P10	148	no hits

* Acronyms of best hits are listed in Supp. Table S1.
