# Supplementary material for: Novel Tri-Segmented Rhabdoviruses: A Data Mining Expedition Unveils the Cryptic Diversity of Cytorhabdoviruses
Source: Viruses. 2023 Dec 10;15(12):2402. doi: 10.3390/v15122402 (PMC10747219; doi:10.3390/v15122402)
Supplement: Supplementary file 1 [file viruses-15-02402-s001.zip › viruses-2733329-supplementary/figures & tables/Table 5.pdf]

Table 5. Consensus conserved plant rhabdovirus gene junction sequences

Proposed Genus	Virus*	3´end mRNA	intergenic spacer	5´end mRNA
Alphacytorhabdovirus	ArcACRV1	AAUUAUUUU	GAU	CUU
	ArtACRV1	AAUUCUUUU	$GA(U)_n$	CNN
	ArtACRV2	AAUUAUUUU	$GA(U)_n$	CNN
	ArtACRV3	AAUUAUUUU	$GA(U)_n$	CNU
	BacACRV1	AAUUCUUUU	$GA(U)_n$	CNC
	CarACRV1	AAUUAUUUU	GAU	CUU
	CheACRV1	AAUUAUUUU	GAU	CUU
	ChrACRV1	AAAUAUUUU	GAU	CUU
	ConACRV1	AAUUCUUUU	GAU	CNC
	CynACRV1	AAUU(C/A)UUUU	$GA(U)_n$	CNN
	EupACRV1	AAUUAUUUU	GAU	CUU
	FagACRV1	AAUUAUUUU	GAU	CNN
	FicACRV1	AAUUAUUUU	GAU	CNN
	GarACRV1	AAUUCUUUU	$GN(U)_n$	CNN
	GeuACRV1	AAUUCUUUU	GAU	CNC
	HedACRV1	AAUUCUUUU	GNU	CNC
	IleACRV1	AAUUAUUUU	$GA(U)_n$	CUG
	MedACRV1	AAUUAUUUU	GAU	CNN
	MenACRV1	AAUUAUUUU	GAU	CUU
	MorACRV1	AAUUCUUUU	GNU	CNN
	OakACRV1	AAUUAUUUU	GAU	CUU
	OciACRV1	AAUUAUUUU	GAU	CUU
	PelACRV1	AAUUAUUUU	GAU	CUN
	PhyACRV1	AAUUCUUUU	GAU	CUC
	PinACRV1	AAUUAUUUU	$GN(U)_n$	CU(U/G)
	PogACRV1	AAUUCUUUU	$G(N)_n$	CUC
	PogACRV2	AAUUAUUUU	GAU	CNN
	PogACRV3	AAUUAUUUU	GAU	CNG
	PriACRV1	AAUUCUUUU	$GA(U)_n$	CUN
	PriACRV2	CAUUAUUUU	GAU	CUG
	RosACRV1	AAUUAUUUU	GAU	CUN
	RubACRV1	AAUUAUUUU	GNU	CNN
	ScuACRV1	AAUUAUUUU	$G(N)_n$	CNN
	TolACRV1	AAUUCUUUU	GNU	CUC
	TriACRV1	AAUUAUUUU	$GA(U)_n$	CU(G/U)
	UtrACRV1	AAUUAUUUU	$GA(U)_n$	CNN
	WMaACRV1	AAUUCUUUU	GAU	CUU
	WurACRV1	AAUUAUUUU	$GN(U)_n$	CNN
	ZeaACRV1	AUUUAUUUU	$GA(U)_n$	CNN
	AcCV	AAUUAUUUU	GAU	CUG
	ADV	AAUUAUUUU	GAU	CUU
	AscSyV1	AAUUAUUUU	GNU	CNN
	BCRV2	AAUUCUUUU	GNU	CNN
	BmV1	AAUUAUUUU	GAN	CUG
	CCyV1	AAUUCUUUU	$G(N)_n$	CUU
	ChYDaV	AAUUAUUUU	GAU	CUN
	CCRV1	AAUUAUUUU	GAU	CUU
	CnV2	AAUUAUUUU	GAU	CUN
	DV1	AAUUAUUUU	GAU	CUG
	GlLV1	AAUUAUUUU	GAU	CUU
	HpLV	AAUUAUUUU	GAU	CNN

	KePCyV	AAUUAUUUU	GAU	CUU
	LNYV	AAUUCUUUU	$G(N)_n$	CUU
	LYMoV	AAUUCUUUU	$G(N)_n$	CUN
	NymAV1	AUUAAUUUU	GAU	CUN
	PaCRV1	AAUUAUUUU	GAU	CUU
	PCaCV	AAUUAUUUU	GNU	CUN
	PeVA	AAUUAUUUU	$G(N)_n$	CUN
	PNSaV	AAUUAUUUU	GAU	CUN
	RVCV	AUUUAUUUU	GAU	CUU
	SaV1	AUUUAUUUU	GAU	CNN
	SCV	AAUUAUUUU	GAU	CUU
	StrV1	AAUUAUUUU	GAU	CUU
	StrV2	AAUUCUUUU	GNU	CNN
	TCRV1	AAUUAUUUU	GAU	CNN
	TpVA	AAUUAUUUU	GAU	CUU
	TpVB	AAUUCUUUU	$G(N)_n$	CUN
	TrARV1	AAUUAUUUU	GAU	CUU
	TYMaV	AAUUAUUUU	GAU	CUU
	WhIV4	AAUUAUUUU	GNU	CUU
	WhIV5	AAUUAUUUU	GAU	CNN
	WhIV6	AAUUAUUUU	GAU	CUN
Betacytorhabdovirus	ArtBCRV1	AUUCUUUUU	GUU	CUU
	BegBCRV1	AUAUUUUUU	GN	CUN
	BetBCRV1	AUUCUUUUU	$GG(U)_n$	CUG
	BetBCRV2	AUUCUUUUU	$GG(U)_n$	CUG/A
	BouBCRV1	AUUCUUUUU	GCU	CUG
	ChrBCRV1	AUUCUUUUU	GUU	CUU
	CorBCRV1	AUUCUUUUU	GGUU	CUG
	CucBCRV1	AUUCUUUUU	$G(N)_n$	CUU
	CypBCRV1	UUCUUUUUU	GA	CUC
	DryBCRV1	AUUAUUUUU	GGU	CCU
	DurBCRV1	AUUCUUUUU	GA	CUC
	GleBCRV1	AUUAUUUUU	$GG(U)_n$	CUN
	GlyBCRV1	AUUAUUUUU	GGU	CCU
	HepBCRV1	AUUAUUUUU	$GA(U)_n$	CUU
	HowBCRV1	AUAUUUUUU	GA	CUN
	IpoBCRV1	AUUCUUUUU	GUU	CUN
	JusBCRV1	AUU(A/C)UUUUU	GGUU	CUN
	KobBCRV1	AUUCUUUUU	GGN	CUC
	Leu CRV1	AUUCUUUUU	GA	CUC
	LycBCRV1	AUUAUUUUU	GGU	CCU
	ManBCRV1	AUUAUUUUU	$GG(U)_n$	CUN
	MorBCRV1	AUUAUUUUU	GGU	CCU
	NitBCRV1	AUUCUUUUU	GGUU	CUN
	PanBCRV1	AUUCUUUUU	G(G/A)	CUC
	PasBCRV1	AUAUUUUUU	GAUU	CUC
	PSaBCRV1	AUUUAUUUU	GA	CUC
	PSaBCRV2	AUUAUUUUU	GNU	CUN
	PenBCRV1	AUAUUUUUU	$G(N)_n$	CUU
	PheBCRV1	AUUAUUUUU	GGUU	CUC
	PopBCRV1	AUUCUUUUU	$GG(U)_n$	CUN
	PueBCRV1	AUUAUUUUU	GGU	CCU
	SesBCRV1	UUCUUUUUU	GA	CUN
	SchBCRV1	AUUCUUUUU	GA	CUC

	SopBCRV1	AUUAUUUUU	GGU	CCU
	TriBCRV1	AUUCUUUUU	GN	CUN
	VicBCRV1	AUUCUUUUU	GG	CUC
	ZanBCRV1	AUUAUUUUU	GGU	CCU
	ZanBCRV2	AUUAUUUUU	GGU	CCU
	ZanBCRV3	AUUAUUUUU	GGU	CCU
	AntAmV1	AUUAUUUUU	GCU	CUU
	AriACRV	UUAUUUUUU	GN(N)$_n$	CNN
	BeTaV1	UUAUUUUUU	GA	CUC
	BYSMV	AUUAUUUUU	GA	CUC
	CBDaV	AUUCUUUUU	GG	CUC
	CuCV1	AUUAUUUUU	GA	CUC
	MaCyV	AUUCUUUUU	GA	CUC
	MYSV	AUUAUUUUU	GA	CUC
	NCMV	AUUCUUUUU	GA	CUC
	PMuMaV	AUUAUUUUU	G(N)$_n$	CUA
	PpVE	AUUCUUUUU	GAC	CCU
	RaCV	AUUCUUUUU	G(N)$_n$	CUN
	RVR	AUUUAUUUU	GA	CUC
	RSMV	AUUCUUUUU	GCU	CUG
	RudV1	AUUCUUUUU	GGUU(N)$_n$	CUN
	SbBMV	UUAUUUUUU	GA	CAC
	TaEV1	AUUCUUUUU	GG(N)$_n$	CUN
	TiCRV1	AUAUUUUUU	GA(N)$_n$	CUC
	YmCaV	UUAUUUUUU	GA	CUC
	YmVA	AUUCUUUUU	GGU	CCU
Gammacytorhabdovirus	ArgGCRV1	AUUCUUUUU	AAU	CCU
	CarGCRV1	AUUCUUUUU	A(N)$_n$	CCU
	CelGCRV1	AUUCUUUUU	A(N)$_n$	CNU
	CopGCRV1	AUUCUUUUU	A(N)$_n$	CCU
	CusGCRV1	AUUCUUUUU	A(N)$_n$	CNN
	CusGCRV2	AUUCUUUUU	A(N)$_n$	CCU
	CypGCRV1	AAUCUUUUU	A(N)$_n$	CNN
	EpiGCRV1	AUUCUUUUU	AUGU	CCU
	FraGCRV1	AUUCUUUUU	A(N)$_n$	CNU
	FraGCRV2	AUUCUUUUU	A(N)$_n$	CCU
	HelGCRV1	AUUCUUUUU	A(N)$_n$	CCU
	HibGCRV1	AUUCUUUUU	A(N)$_n$	CNN
	LonGCRV1	AUUCUUUUU	A(N)$_n$	CCU
	LupGCRV1	AUUCUUUUU	A(N)$_n$	CCU
	Rh GCRV1	AUUUCUUUU	A(N)$_n$	CCU
	SilGCRV1	AUUCUUUUU	A(N)$_n$	CCU
	GymDenV1	AAUCUUUUU	A(N)$_n$	CNN
	TrAV1	AUUCUUUUU	A(N)$_n$	CNU
Trirhavirus	AlTRV1	AAUUCUUUU	GN(N)$_n$	CUC
	ChTRV1	AAUUCUUUU	GN(N)$_n$	CCU
	EryTRV1	AAUUCUUUU	GN(N)$_n$	CUC
	MeTRV1	AAUUCUUUU	GN(N)$_n$	CU (C/G)
	PiTRV1	AAUUCUUUU	GN(N)$_n$	CUN

The consensus gene junction sequences of the viruses identified in this study are highlighted in light grey. * Names and abbreviations of newly identified viruses are listed in Tables 1-4; while the names and abbreviations of known viruses are listed in Supp Table 1.
