# Supplementary material for: Novel Tri-Segmented Rhabdoviruses: A Data Mining Expedition Unveils the Cryptic Diversity of Cytorhabdoviruses
Source: Viruses. 2023 Dec 10;15(12):2402. doi: 10.3390/v15122402 (PMC10747219; doi:10.3390/v15122402)
Supplement: Supplementary file 1 [file viruses-15-02402-s001.zip › viruses-2733329-supplementary/supp files/Fig. S1.pdf]

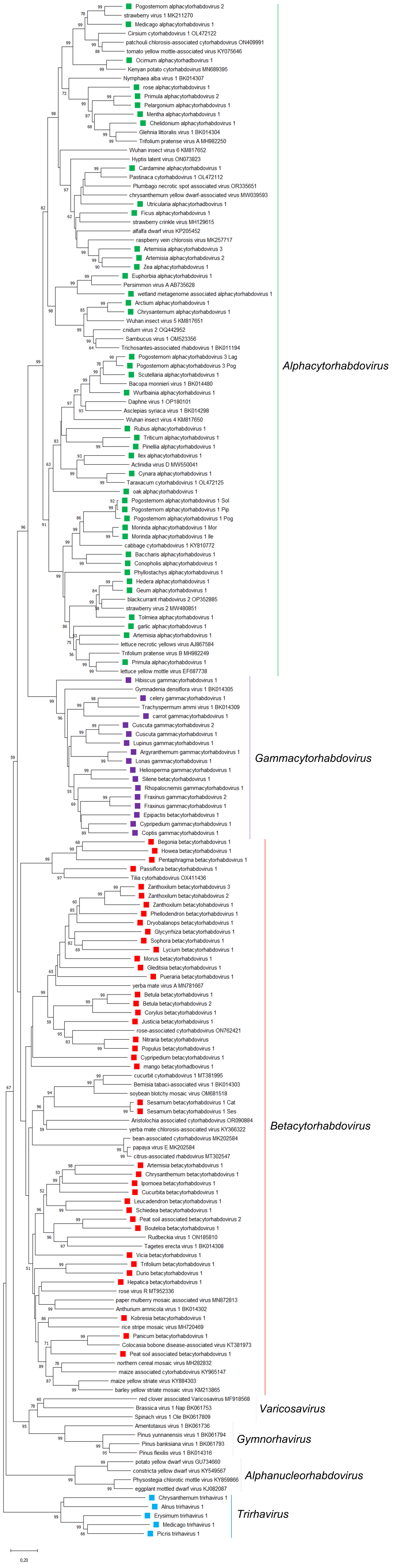
Pogostemom alphacytorhabdovirus 2
strawberry virus 1 MK211270
Medicago alphacytorhabdovirus 1
Cirsium cytorhabdovirus 1 OL472122
patchouli chlorosis-associated cytorhabdovirus ON409991
tomato yellow mottle-associated virus KY075646
Ocimum alphacytorhabdovirus 1
Kenyan potato cytorhabdovirus MN689395
Nymphaea alba virus 1 BK014307
rose alphacytorhabdovirus 1
Primula alphacytorhabdovirus 2
Pelargonium alphacytorhabdovirus 1
Mentha alphacytorhabdovirus 1
Chelidonium alphacytorhabdovirus 1
Glehnia littoralis virus 1 BK014304
Trifolium pratense virus A MH982250
Wuhan insect virus 6 KM817652
Hyptis latent virus ON073823
Cardamine alphacytorhabdovirus 1
Pastinaca cytorhabdovirus 1 OL472112
Plumbago necrotic spot associated virus OR335651
chrysanthemum yellow dwarf-associated virus MW039593
Utricularia alphacytorhabdovirus 1
Ficus alphacytorhabdovirus 1
strawberry crinkle virus MH129615
alfalfa dwarf virus KP205452
raspberry vein chlorosis virus MK257717
Artemisia alphacytorhabdovirus 3
Artemisia alphacytorhabdovirus 2
Zea alphacytorhabdovirus 1
Euphorbia alphacytorhabdovirus 1
Persimmon virus A AB735628
wetland metagenome associated alphacytorhabdovirus 1
Arctium alphacytorhabdovirus 1
Chrysantemum alphacytorhabdovirus 1
Wuhan insect virus 5 KM817651
cnidium virus 2 OQ442952
Sambucus virus 1 OM523356
Trichosantes-associated rhabdovirus 1 BK011194
Pogostemom alphacytorhabdovirus 3 Lag
Pogostemom alphacytorhabdovirus 3 Pog
Scutellaria alphacytorhabdovirus 1
Bacopa monnieri virus 1 BK014480
Wurfbainia alphacytorhabdovirus 1
Daphne virus 1 OP180101
Asclepias syriaca virus 1 BK014298
Wuhan insect virus 4 KM817650
Rubus alphacytorhabdovirus 1
Triticum alphacytorhabdovirus 1
Pinellia alphacytorhabdovirus 1
Ilex alphacytorhabdovirus 1
Actinidia virus D MW550041
Cynara alphacytorhabdovirus 1
Taraxacum cytorhabdovirus 1 OL472125
oak alphacytorhabdovirus 1
Pogostemom alphacytorhabdovirus 1 Sol
Pogostemom alphacytorhabdovirus 1 Pip
Pogostemom alphacytorhabdovirus 1 Pog
Morinda alphacytorhabdovirus 1 Mor
Morinda alphacytorhabdovirus 1 Ile
cabbage cytorhabdovirus 1 KY810772
Baccharis alphacytorhabdovirus 1
Conopholis alphacytorhabdovirus 1
Phyllostachys alphacytorhabdovirus 1
Hedera alphacytorhabdovirus 1
Geum alphacytorhabdovirus 1
blackcurrant rhabdovirus 2 OP352885
strawberry virus 2 MW480851
Tolmiea alphacytorhabdovirus 1
garlic alphacytorhabdovirus 1
Artemisia alphacytorhabdovirus 1
lettuce necrotic yellows virus AJ867584
Trifolium pratense virus B MH982249
Primula alphacytorhabdovirus 1
lettuce yellow mottle virus EF687738
Alphacytorhabdovirus
Hibiscus gammacytorhabdovirus 1
Gymnadenia densiflora virus 1 BK014305
celery gammacytorhabdovirus 1
Trachyspermum ammi virus 1 BK014309
carrot gammacytorhabdovirus 1
Cuscuta gammacytorhabdovirus 2
Cuscuta gammacytorhabdovirus 1
Lupinus gammacytorhabdovirus 1
Argyranthemum gammacytorhabdovirus 1
Lonas gammacytorhabdovirus 1
Heliosperma gammacytorhabdovirus 1
Silene betacytorhabdovirus 1
Rhopalocnemis gammacytorhabdovirus 1
Fraxinus gammacytorhabdovirus 2
Fraxinus gammacytorhabdovirus 1
Epipactis betacytorhabdovirus 1
Cypripedium gammacytorhabdovirus 1
Coptis gammacytorhabdovirus 1
Gammacytorhabdovirus
Begonia betacytorhabdovirus 1
Howea betacytorhabdovirus 1
Pentaphragma betacytorhabdovirus 1
Passiflora betacytorhabdovirus 1
Tilia cytorhabdovirus OX411436
Zanthoxilum betacytorhabdovirus 3
Zanthoxilum betacytohabdovirus 2
Zanthoxilum betacytorhabdovirus 1
Phellodendron betacytorhabdovirus 1
Dryobalanops betacytorhabdovirus 1
Glycyrrhiza betacytorhabdovirus 1
Sophora betacytorhabdovirus 1
Lycium betacytorhabdovirus 1
Morus betacytorhabdovirus 1
Gleditsia betacytorhabdovirus 1
Pueraria betacytorhabdovirus 1
yerba mate virus A MN781667
Betula betacytorhabdovirus 1
Betula betacytorhabdovirus 2
Corylus betacytorhabdovirus 1
Justicia betacytorhabdovirus 1
rose-associated cytorhabdovirus ON762421
Nitraria betacytorhabdovirus
Populus betacytorhabdovirus 1
Cypripedium betacytorhabdovirus 1
mango betacytorhabdovirus 1
cucurbit cytorhabdovirus 1 MT381995
Bemisia tabaci-associated virus 1 BK014303
soybean blotchy mosaic virus OM681518
Sesamum betacytorhabdovirus 1 Cat
Sesamum betacytorhabdovirus 1 Ses
Aristolochia associated cytorhabdovirus OR090884
yerba mate chlorosis-associated virus KY366322
bean-associated cytorhabdovirus MK202584
papaya virus E MK202584
citrus-associated rhabdovirus MT302547
Artemisia betacytorhabdovirus 1
Chrysanthemum betacytorhabdovirus 1
Ipomoea betacytorhabdovirus 1
Cucurbita betacytorhabdovirus 1
Leucadendron betacytorhabdovirus 1
Schiedea betacytorhabdovirus 1
Peat soil associated betacytorhabdovirus 2
Bouteloa betacytorhabdovirus 1
Rudbeckia virus 1 ON185810
Tagetes erecta virus 1 BK014308
Vicia betacytorhabdovirus 1
Trifolium betacytorhabdovirus 1
Durio betacytorhabdovirus 1
Hepatica betacytorhabdovirus 1
rose virus R MT952336
paper mulberry mosaic associated virus MN872813
Anthurium amnicola virus 1 BK014302
Kobresia betacytorhabdovirus 1
rice stripe mosaic virus MH720469
Panicum betacytorhabdovirus 1
Colocasia bobone disease-associated virus KT381973
Peat soil associated betacytorhabdovirus 1
northern cereal mosaic virus MH282832
maize associated cytorhabdovirus KY965147
maize yellow striate virus KY884303
barley yellow striate mosaic virus KM213865
Betacytorhabdovirus
red clover associated Varicosavirus MF918568
Brassica virus 1 Nap BK061753
Spinach virus 1 Ole BK061809
Varicosavirus
Amentotaxus virus 1 BK061736
Pinus yunnanensis virus 1 BK061794
Pinus banksiana virus 1 BK061793
Pinus flexilis virus 1 BK014316
Gymnorhavirus
potato yellow dwarf virus GU734660
constricta yellow dwarf virus KY549567
Physostegia chlorotic mottle virus KY859866
eggplant mottled dwarf virus KJ082087
Alphanucleorhabdovirus
Chrysanthemum trirhavirus 1
Alnus trirhavirus 1
Erysimum trirhavirus 1
Medicago trirhavirus 1
Picris trirhavirus 1
Trirhavirus
0.20
