# Supplementary figures and images for: Novel Tri-Segmented Rhabdoviruses: A Data Mining Expedition Unveils the Cryptic Diversity of Cytorhabdoviruses
Source: Viruses. 2023 Dec 10;15(12):2402. doi: 10.3390/v15122402 (PMC10747219; doi:10.3390/v15122402)

A

B

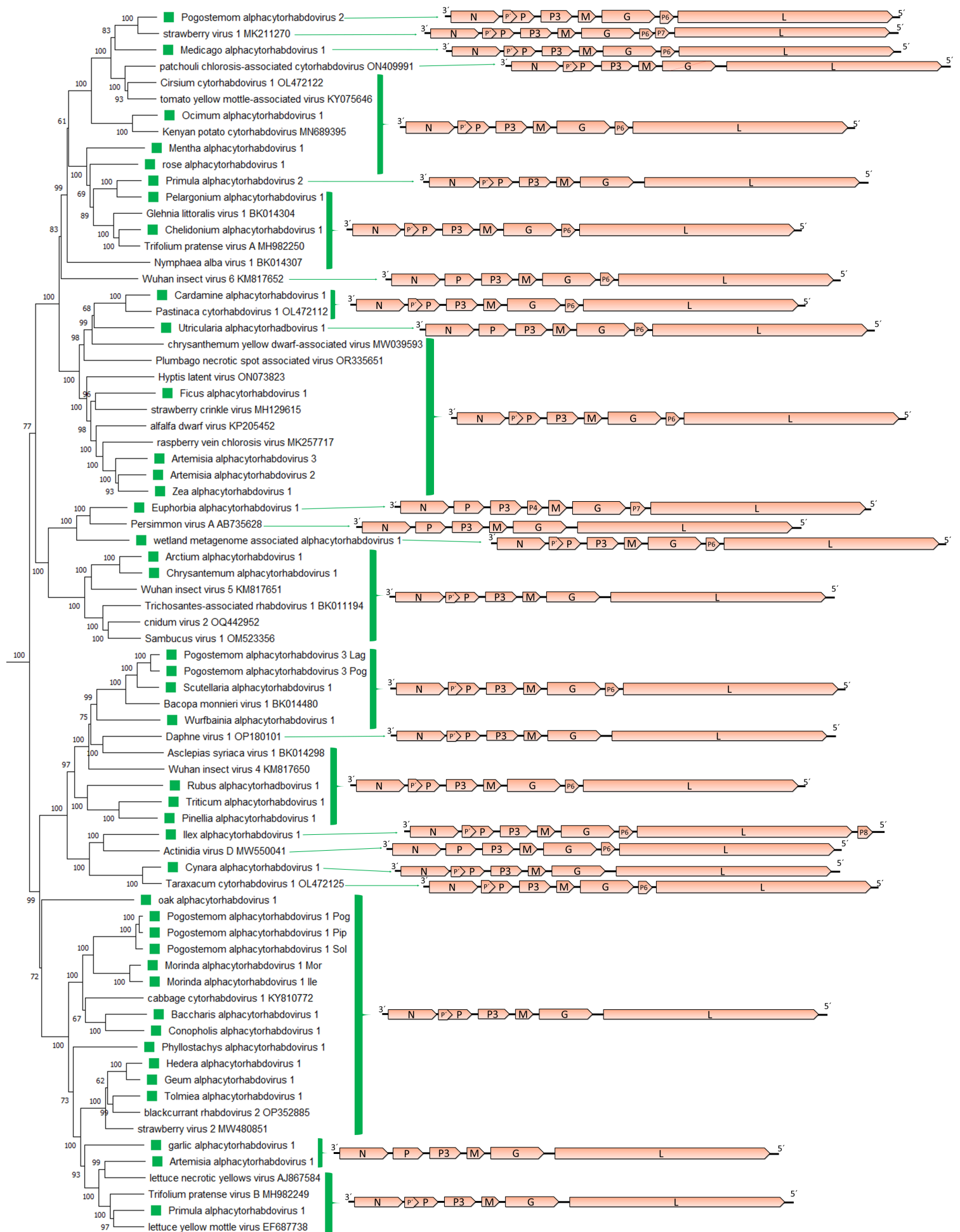

Supplement: Supplementary file 1 [file viruses-15-02402-s001.zip › viruses-2733329-supplementary/figures & tables/Figure 2.pdf]

A

B

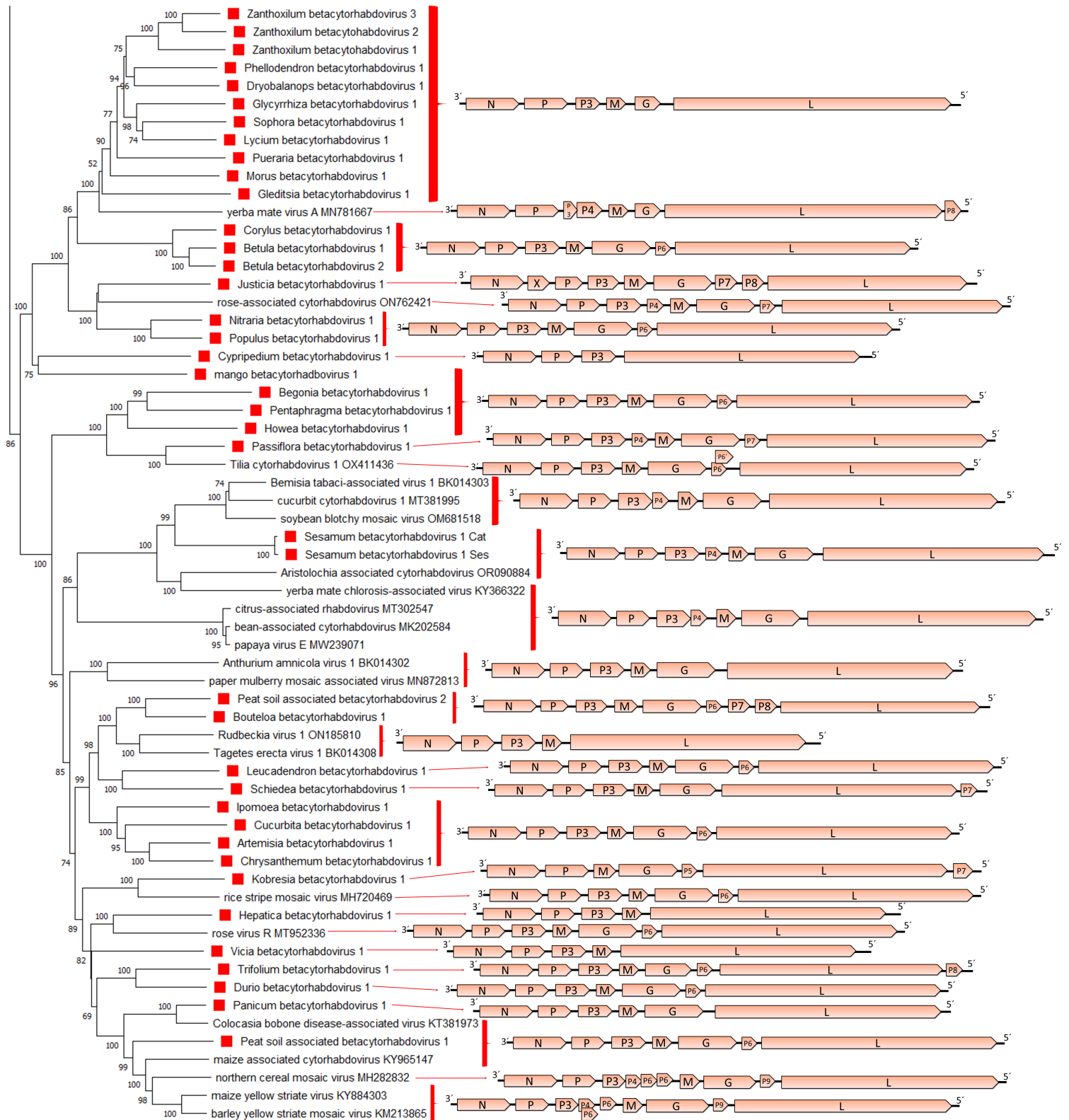

Supplement: Supplementary file 1 [file viruses-15-02402-s001.zip › viruses-2733329-supplementary/figures & tables/Figure 3.pdf]

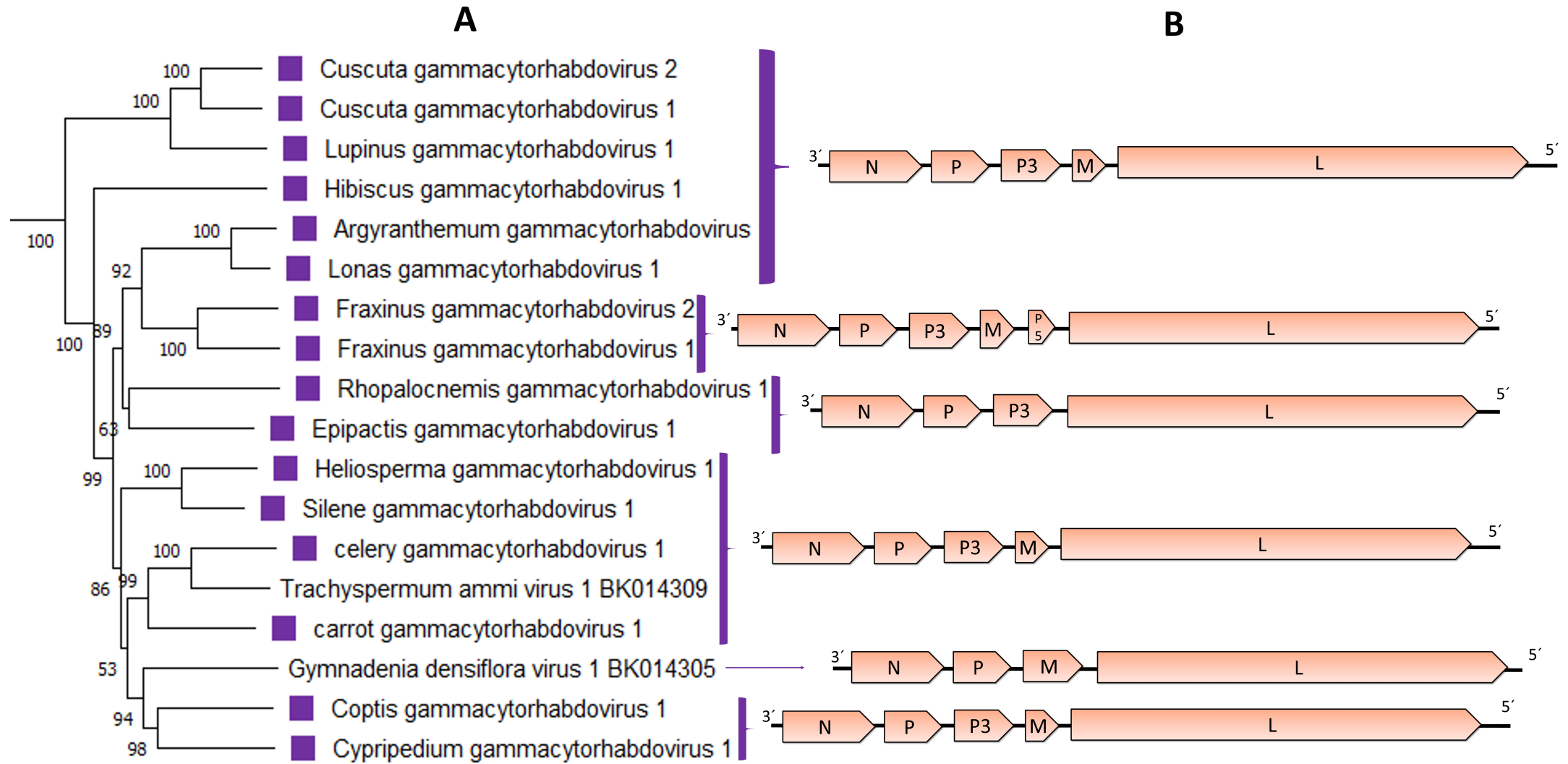

Supplement: Supplementary file 1 [file viruses-15-02402-s001.zip › viruses-2733329-supplementary/figures & tables/Figure 4.pdf]
